# Evaluating Protein Enrichment Methods to Improve Biomarker Discovery in Equine Cerebrospinal Fluid

**DOI:** 10.1002/vms3.70933

**Published:** 2026-04-10

**Authors:** Foti Federico, Wilson Amie, Chabronova Alzbeta, Jensen Anders, Lunn Paul David, Peffers Jayne Mandy

**Affiliations:** ^1^ Institute of Infection, Veterinary & Ecological Sciences University of Liverpool Neston UK; ^2^ Institute of Life Course and Medical Sciences, Faculty of Health & Life Sciences University of Liverpool Liverpool UK

**Keywords:** cerebrospinal fluid (CSF), liquid chromatography with tandem mass spectrometry (LC–MS/MS), native digestion, proteomics, ProteoMiner Small‐Capacity Enrichment Kit: PreOmics Enrich‐iST

## Abstract

**Background:**

Cerebrospinal fluid (CSF) is a valuable source of biomarkers for neurological diseases, but detection of low‐abundance proteins is often masked by highly abundant proteins. Enrichment strategies can enhance proteomic coverage and improve biomarker discovery, yet comparative evaluations of such methods in equine CSF are limited.

**Objectives:**

This study compared the ProteoMiner Small‐Capacity Enrichment Kit and the PreOmics Enrich‐iST Kit for their ability to deplete high‐abundance proteins and enhance detection of low‐abundance proteins relevant to neuropathology.

**Methods:**

Equine CSF samples were processed with either a native in‐solution trypsin digestion without further enrichment, ProteoMiner Small‐Capacity Enrichment Kit, or PreOmics enrichment. Samples were analysed by label‐free liquid chromatography–tandem mass spectrometry. Proteins were identified and quantified using emPAI scores, and gene ontology pathway analyses were performed to evaluate enrichment efficiency and biological relevance.

**Results:**

The PreOmics Enrich‐iST Kit identified the highest number of proteins overall, including neurobiology‐relevant low‐abundance proteins not detected by other methods, and achieved superior depletion of high‐abundance proteins. Gene ontology pathway analysis revealed broader enrichment of neuropathology‐relevant pathways.

**Conclusions:**

The PreOmics Enrich‐iST Kit outperformed the ProteoMiner Small‐Capacity Enrichment Kit and native digestion in equine CSF proteomics, providing greater depletion of high‐abundance proteins and enhanced detection of neurobiology‐relevant low‐abundance proteins. This method offers a robust tool for comprehensive proteomic profiling and may facilitate the discovery of novel biomarkers for equine neurological disorders.

## Graphical abstract

Here, we compared two protein enrichment methods—ProteoMiner Small‐Capacity and PreOmics Enrich‐iST—for proteomic analysis of equine cerebrospinal fluid (CSF). PreOmics Enrich‐iST achieved superior depletion of high‐abundance proteins and identified a greater number of low‐abundance, neuropathology‐relevant proteins than ProteoMiner or native digestion. These findings demonstrate that PreOmics Enrich‐iST enables deeper CSF proteome coverage and supports its use as an effective tool for biomarker discovery in equine neurological disease research.

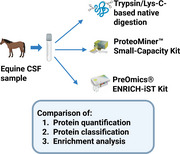

## 1 Introduction

Cerebrospinal fluid (CSF) is an ultrafiltrate of plasma that circulates within the brain ventricles and the subarachnoid spaces of the cranium and spine. It is produced primarily through the ultrafiltration of blood plasma by choroidal capillaries, with ∼20% actively secreted by ependymal cells lining the brain ventricles (Jankovska et al. 2019). As CSF is in direct contact with the central nervous system (CNS; Jankovska et al. 2019), its protein composition reflects its physiological and pathological state. An accurate analysis of CSF protein profiles, including low‐abundance proteins (LAPs), can help us understand the biochemical changes associated with neurological diseases such as equine herpesvirus myeloencephalopathy (EHM; Lunn et al. 2024). Currently, there are no reliable biomarkers to detect or differentiate EHM from other equine neurological diseases, and the validation of novel methods to identify biomarkers could enable earlier and more accurate diagnoses.

While CSF from a healthy horse typically has a much lower protein content than plasma, the protein analysis of CSF is hindered by the wide dynamic range of protein concentrations and by the presence of highly abundant proteins (HAPs), constituting over 80% of the total protein content of CSF (Jankovska et al. 2019; Seeliger et al. 2023; Boschetti and Righetti 2023; Anderson and Ng 2002).

Direct analysis of the native CSF sample without prior depletion of HAPs or enrichment of LAPs has been previously undertaken (Broccardo et al. 2014; Nunez Galindo et al. 2015). While relatively simple and cost‐effective, this approach has only limited ability to detect LAPs. The depletion (at least partial) of HAPs prior to liquid chromatography–tandem mass spectrometry (LC–MS/MS) analysis is necessary to identify a greater dynamic range of proteins in CSF (Wu et al. 2016). To address this, enrichment techniques and kits have been developed, including ProteoMiner Enrichment Kits by Bio‐Rad and PreOmics Enrich‐iST by PreOmics.

Briefly, the ProteoMiner Small‐Capacity Enrichment Kit (Bio‐Rad) uses a combinatorial hexapeptide library bound to chromatographic beads to reduce HAPs in samples (Li 2015), whereby HAPs saturate their ligands and excess protein is washed away. We have successfully used this approach to analyse equine peritoneal fluid, plasma and synovial fluid (Anderson et al. 2020; Bardell et al. 2019). The PreOmics ENRICH‐iST Kit (PreOmics) employs paramagnetic beads with hydrophobic and charge‐based interactions to capture peptides following digestion, enabling efficient removal of contaminants and HAPs and enrichment of LAPs (Corporation B 2023).

This study was designed as a pilot, exploratory comparison of protein enrichment strategies in equine CSF with the primary aim of evaluating technical performance and proteome coverage. While originally designed for blood serum and plasma samples, this study was the first to test whether PreOmics Enrich‐iST could be used with equine CSF. Here, we compared three different approaches to prepare CSF samples for proteomic LC–MS/MS analysis: our laboratory protocol for in‐solution trypsin digestion (native digestion; Anderson et al. 2020), Bio‐Rad's ProteoMiner Small‐Capacity Enrichment Kit, and the PreOmics’ PreOmics Enrich‐iST Kit. Our aim was to determine which approach identified the greatest number of proteins, effectively depleting HAPs, while also enriching LAPs including any neuropathology‐relevant proteins.

## 2 Materials and Methods

### 2.1 Sample Details and Processing

CSF was obtained immediately post‐mortem from a 13‐year‐old mare euthanised due to orthopaedic injury with no clinical history of neurological disease, minimising proteolytic and biochemical changes. The sample was collected via centesis via the atlanto‐occipital approach, centrifuged at 13,000 × *g* for 10 min to remove any particulates and debris, and stored at −80°C until analysis. Each sample was processed in duplicate. Ethics approval was obtained through the University of Liverpool Veterinary Ethics Committee (RETH000553).

### 2.2 Sample Processing: Native Digestion, ProteoMiner Small‐Capacity Kit, and PreOmics Enrich‐iST Kit

The protein concentration of the CSF samples was determined using the bicinchoninic acid (BCA) assay following the manufacturer's protocol (ThermoScientific Pierce BCA Protein Assay Kit, Cat. No. 23225). Native in‐solution trypsin digestion (*n* = 2, technical replicates) was performed with 100 µg of protein as previously reported (Anderson et al. 2020; native). The ProteoMiner enrichment (Bio‐Rad ProteoMiner Small‐Capacity Kit no. 163‐3006) was carried out using 1 mg of protein (*n* = 2, technical replicates). Our adapted protocol (Anderson et al. 2020) was used for column preparation, sample binding, and wash. Subsequent on‐beads digestion was performed as per the native digestion protocol described above. The PreOmics enrichment method (PreOmics ENRICH‐iST Kit 8x P.O.00163) was performed following the manufacturer's instructions using 20 µg of protein (*n* = 2, technical replicates).

### 2.3 Liquid Chromatography–Tandem Mass Spectrometry

LC–MS/MS spectral acquisition was performed as described previously (Johnson et al. 2024). A Q‐Exactive HF‐X Hybrid Quadropole‐Orbitrap mass spectrometer (ThermoScientific) was used, and data‐dependent acquisition (DDA) consisted of a 60,000‐resolution full‐scan MS scan in the orbitrap (AGC set to 3e6 ions with a maximum fill time of 100 ms). Each sample was separated on a 1‐h gradient with 30 min blanks between, as previously described (Peffers et al. 2014).

### 2.4 Data Analysis

The raw data files were processed via an in‐house MASCOT server (Matrix Science, London, UK). The Unihorse database was used with the following search parameters: peptide mass tolerance, 10.0 ppm; fragment mass tolerance, 0.01 Da; enzyme, trypsin; missed cleavages allowed, one; fixed modifications, carbamidomethylation (cysteine) and variable modifications; and oxidation (methionine). Only proteins identified in both replicates per method were considered.

The exponentially modified protein abundance index (emPAI) score was employed as a quantitative measure of protein abundance (Ishihama et al. 2005). Each protein's emPAI score was averaged between the technical duplicates and compared between each technique. Enrichment was indicated by an increase in the emPAI score, whereas depletion was indicated by a decrease in the emPAI score, both of which are expressed as log2‐fold change (log2FC). To determine protein classification and biological pathways from each sample protein identification list, we performed the analyses using the gene name provided by MASCOT against the *Equus caballus* genes on the Protein Analysis Through Evolutionary Relationships (PANTHER) Classification System (Mi et al. 2013) database after applying the false discovery rate (FDR) correction. Pathway analysis was performed using PANTHER and the enrichment analysis based on gene ontology (GO) Biological Process. Pathways with FDR < 0.05 were considered to be significantly enriched. Graphs were plotted using the ggplot2 package (Wickham 2016; v3.3.5) and ggVennDiagram package (Gao 2024; v1.5.2) in R Studio (Team RC 2021; v4.1.2).

## 3 Results

### 3.1 Comparison of Protein Profiles for Native Digestion, ProteoMiner Small‐Capacity Enrichment Kit and PreOmics Enrich‐iST Kit

**FIGURE 1:**
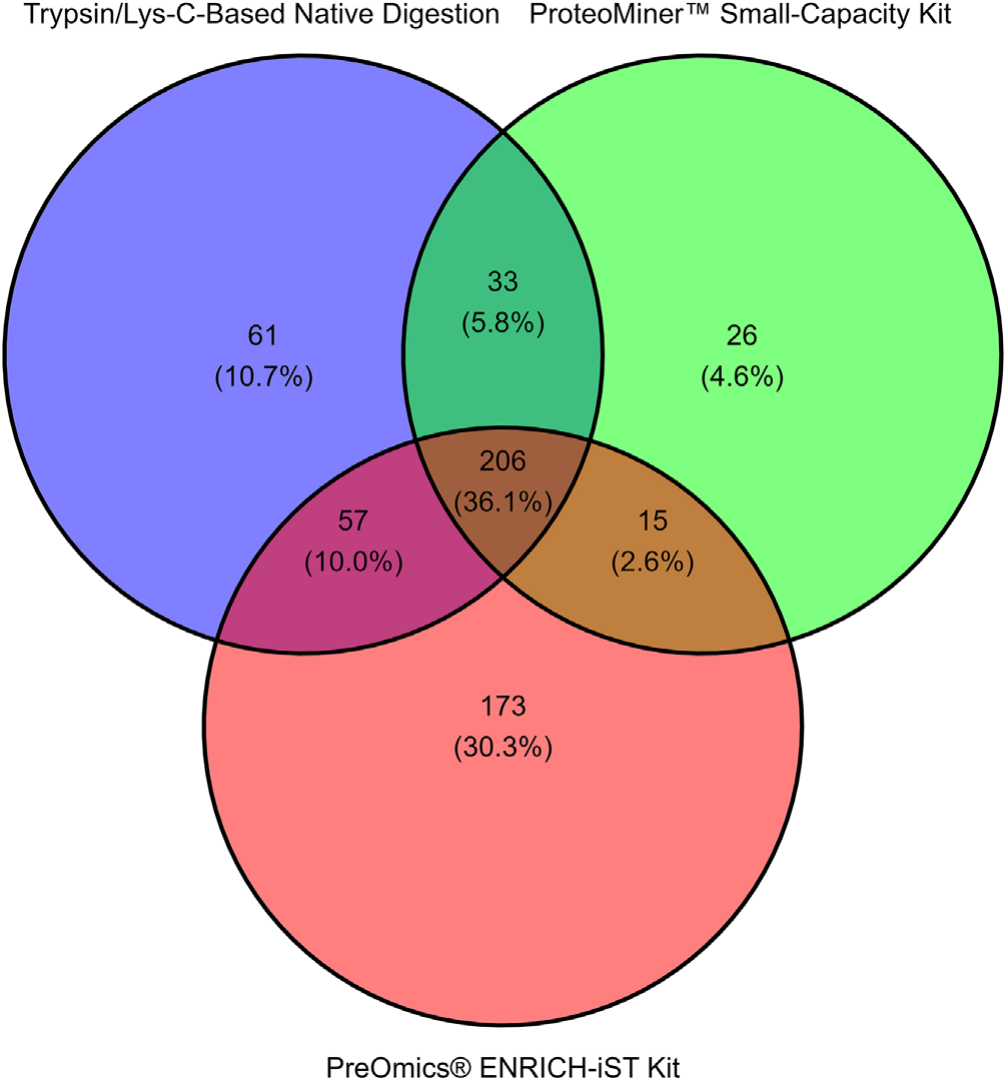
Overlapping and unique proteins identified by native digestion, ProteoMiner Small‐Capacity Enrichment Kit, and PreOmics Enrich‐iST Kit. CSF samples were processed using these three methods, followed by LC–MS/MS, DDA, and MASCOT identification. The graph shows the overlap of proteins identified by MASCOT in both replicates for given method. The Venn diagram was generated using the ggVennDiagram package15 (v1.5.2) in R Studio16 (v4.1.2).

We compared three CSF preparation approaches for LC–MS/MS analysis: in‐solution trypsin digestion (native, used as control; Anderson et al. 2020), ProteoMiner Small‐Capacity Enrichment Kit, and PreOmics Enrich‐iST, aiming to evaluate HAPs depletion, overall protein identification, and enrichment of neuropathology‐relevant LAPs. In total, we identified 382 proteins in the samples processed with native digestion, 301 proteins in samples processed with the ProteoMiner Small‐Capacity Enrichment Kit, and 487 proteins in samples processed with the PreOmics Enrich‐iST Kit, with the latter yielding the highest number overall. The number of proteins identified by each technique was visualised using a Venn diagram to highlight overlapping and unique proteins identified in each method (Figure 1), with 571 proteins identified across all techniques (100%). Of these, 206 (36.1%) were common to all methods, 61 (10.7%) were unique to native digestion, 26 (4.6%) to The ProteoMiner Small‐Capacity Enrichment Kit, and 173 (30.3%) to the PreOmics Enrich‐iST Kit. Comprehensive tables listing all proteins identified by each method can be found in Supporting Information Tables 1.1–1.3, and their overlaps in Supporting Information Table 1.4.

Among shared proteins (*n* = 206), some were well‐documented HAPs within CSF (Broccardo et al. 2014; Ahmed et al. 2022; Donnelly et al. 2023; Kroksveen et al. 2011) such as prostaglandin‐H2 D‐isomerase (PGD2), as well as apolipoprotein E (APOE), and Cystatin C (CST3; Martins‐De‐Souza et al. 2010). Neuropathology‐relevant LAPs included neurofascin (NFASC; Ogata et al. 2015), neuronal cell adhesion molecule (NRCAM; Leshchyns'ka and Sytnyk 2016), semaphorin 7A (SEMA7A; Gutierrez‐Franco et al. 2017) and brevican (BCAN; Frischknecht and Seidenbecher 2012). Within the 15 proteins identified by both the ProteoMiner Small‐Capacity Enrichment Kit and PreOmics Enrich iST Kit, several LAPs in CSF linked to brain health and blood–brain barrier (BBB) disruption were observed, including peroxiredoxin 2 (PRDX2; Lu et al. 2018), fibrinogen beta chain (FGB; Wen and Zhang 2023), nidogen‐2 (NID2; Xu et al. 2019) and actin beta (ACTB; Li et al. 2023). Among the 26 proteins unique to the ProteoMiner Small‐Capacity Enrichment Kit, LAPs associated with neuropathologies included Dickkopf WNT signalling pathway inhibitor 3 (DKK3; Podpolny et al. 2024) and Cyclin‐dependent kinase 1 (CDK1; Lukasik et al. 2021).

The 173 proteins unique to the PreOmics Enrich‐iST Kit included Pyruvate kinase (PKM; de Geus et al. 2025), Meteorin like, glial cell differentiation regulator (METRNL; Berghoff et al. 2021), Peroxiredoxin‐1 (PRDX1; Szeliga 2020), Semaphorin 4D (SEMA4D; Smith et al. 2015), lemur tyrosine kinase 3 (LMTK3; Larose et al. 2024) and adenylate cyclase type 5 (ADCY5; Kozon et al. 2024).

### 3.2 The PreOmics Enrich‐iST Kit Outperformed the ProteoMiner Small‐Capacity Enrichment Kit in Depleting HAPs

We also assessed the capacity of the ProteoMiner Small‐Capacity Enrichment Kit and PreOmics Enrich‐iST Kit to deplete HAPs in CSF. emPAI scores of known HAPs (e.g., ALB or serotransferrin [TF]) served as a proxy. emPAI scores of proteins identified in the native digest samples were considered baseline against which emPAI scores of proteins identified by the ProteoMiner Small‐Capacity Enrichment Kit and the PreOmics ENRICH‐iST Kit were compared. A lower emPAI score and lower log_2_FC were indicative of more efficient HAP depletion.

**FIGURE 2:**
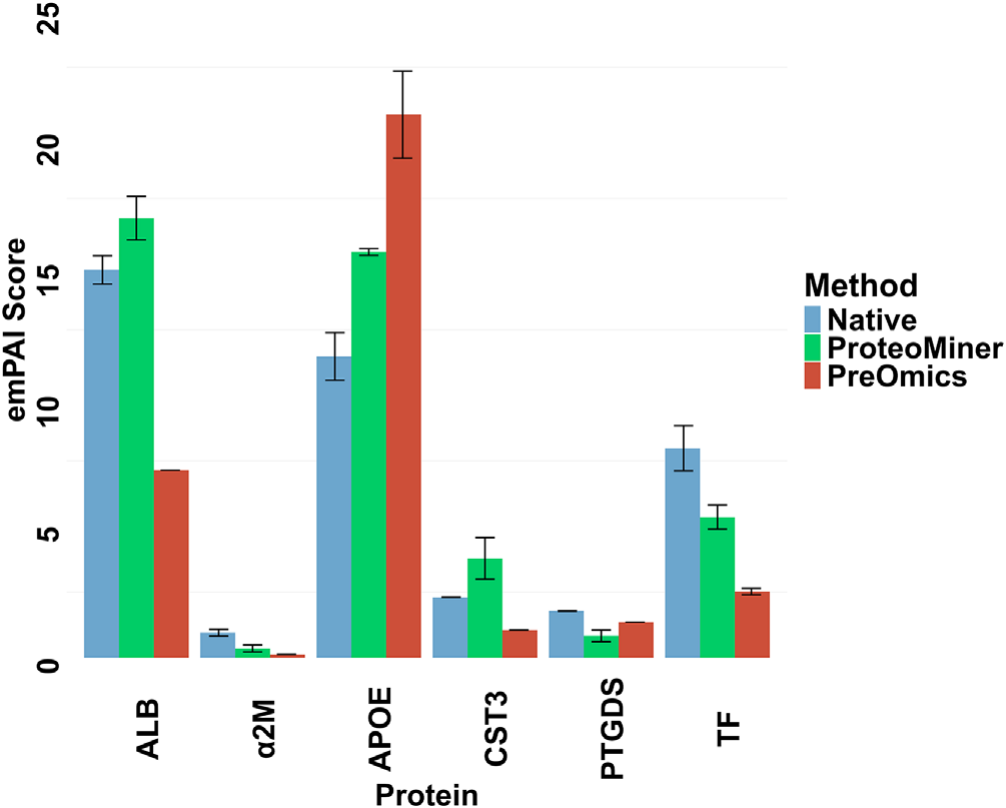
The PreOmics Enrich‐iST Kit outperformed ProteoMiner in depleting HAPs. The bar chart shows the emPAI scores of known HAPs measured using three techniques. Each protein is represented by three bars corresponding to the three techniques: native digestion (‘Native’, blue), ProteoMiner Small‐Capacity Enrichment Kit (‘ProteoMiner’, green) and PreOmics ENRICH‐iST Kit (‘PreOmics’, red). The *y*‐axis represents the emPAI score, and the *x*‐axis shows the proteins analysed. In most cases, the sample processed with the PreOmics ENRICH‐iST Kit exhibited lower emPAI scores across known HAPs. Values (*n* = 2) are expressed as means and ± SD. Image made using ggplot2 (version 3.5.2).

When compared to the native digestion, the abundance of ALB was not reduced in the sample processed with the ProteoMiner Small‐Capacity Enrichment Kit. However, it decreased substantially (log_2_FC = −1.05) in samples processed with the PreOmics Enrich‐iST Kit (Table 1). TF was slightly depleted by the ProteoMiner Small‐Capacity Enrichment Kit (log2FC = −0.58) and greatly depleted by PreOmics Enrich‐iST Kit (log_2_FC = −1.66). Interestingly, the abundance of APOE was increased by both the ProteoMiner Small‐Capacity Enrichment Kit (log_2_FC = 0.43) and PreOmics Enrich‐iST (log_2_FC = 0.85). CST3 was enriched by the ProteoMiner Small‐Capacity Kit (log_2_FC = 0.72) but substantially depleted by PreOmics Enrich‐iST (log_2_FC = −1.13; Figure 2). Table 1 summarises the emPAI scores of selected HAPs identified using the three different methods.

**TABLE 1:** The PreOmics ENRICH‐iST Kit outperformed the ProteoMiner Small‐Capacity Enrichment Kit in HAPs depletion and neuropathology‐relevant LAPs enrichment.

Depletion of HAPs					
	Native Digestion	ProteoMiner Small‐Capacity Enrichment Kit		PreOmics ENRICH‐iST Kit	
Protein	emPAI	emPAI	log2FC	emPAI	log2FC
Albumin (ALB)	14.78	16.75	0.18	7.14	−1.05
Alpha‐2‐macroglobulin (α2M)	0.95	0.35	−1.44	0.12	−2.98
Apolipoprotein E (APOE)	11.48	15.46	0.43	20.7	0.85
Cystatin 3 (CST3)	2.3	3.78	0.72	1.05	−1.13
Prostaglandin 2 (PTGDS)	1.78	0.83	−1.1	1.35	−0.4
Serotransferrin (TF)	7.98	5.35	−0.58	2.52	−1.66

Enrichment of proteins identified by the enrichment methods			
	ProteoMiner Small‐Capacity Enrichment Kit	PreOmics ENRICH‐iST Kit	
Protein	emPAI	emPAI	log2FC
Actin beta (ACTB)	0.39	1.06	1.44
ADP‐ribosylarginine hydrolase (ADPRH)	0.14	0.19	0.44
Extracellular matrix protein 2 (ECM2)	0.08	0.05	−0.68
Family with sequence similarity 91 member A1 (FAM91A1)	0.04	0.04	0.00
Fibrinogen beta chain (FGB)	4.69	3.63	−0.37
Fibronectin (FN1)	1.42	0.75	−0.92
Fibulin 2 (FBLN2)	0.16	0.06	−1.42
Follistatin‐like 5 (FSTL5)	0.08	0.04	−1.00
Inter‐alpha‐trypsin inhibitor heavy chain 2 (ITIH2)	1.20	0.77	−0.64
Keratin 3 (KRT3)	0.53	1.55	1.55
Keratin 77 (KRT77)	0.2	0.62	1.63
Nidogen 2 (NID2)	0.03	0.14	2.22
Olfactomedin‐like 3 (OLFML3)	0.65	0.54	−0.27
Peroxiredoxin 2 (PRDX2)	0.53	0.4	−0.41
Plexin B2 (PLXNB2)	0.02	0.02	0.00
Prenylcysteine oxidase 1 (PCYOX1)	0.11	0.07	−0.65

The top half of the table compares the depletion of HAPs by both kits against the native digestion control. Lower emPAI scores for HAPs, and respective lower log2FC, indicated greater depletion. The emPAI scores are expressed as means, with respective log2FC on their right. The second half of the table compares protein enrichment by ProteoMiner Small‐Capacity Enrichment Kit and PreOmics ENRICH‐iST kit. In this comparison, positive log_2_FC values indicated greater enrichment by the PreOmics ENRICH‐iST Kit, while negative log_2_FC values indicated greater enrichment by the ProteoMiner Small‐Capacity Enrichment Kit.

Abbreviations: emPAI, exponentially modified protein abundance index; HAPs, high‐abundance proteins; log2FC, log2 fold change.

### 3.3 Distinct Protein Enrichment Biases Introduced by the ProteoMiner Small‐Capacity Enrichment Kit and the PreOmics ENRICH‐iST Kit

Next, we compared the enrichment of proteins uniquely identified in samples processed by the two enrichment kits, and absent in the native digestion sample. We assessed the enrichment using the emPAI scores from the ProteoMiner Small‐Capacity Enrichment Kit as a baseline. A higher emPAI score and positive log_2_FC indicated greater protein enrichment by the PreOmics ENRICH‐iST, while higher emPAI score and negative log_2_FC indicated greater protein enrichment by the ProteoMiner Small‐Capacity Enrichment Kit.

The ProteoMiner Small‐Capacity Enrichment Kit preferentially enriched proteins associated with extracellular matrix (ECM) structure and remodelling, including fibulin‐2 (FBLN2; log_2_FC = −1.42) and extracellular matrix protein‐2 (ECM2; log_2_FC = −0.68). Additional ECM‐related proteins enriched by this method included inter‐alpha‐trypsin inhibitor heavy chain‐2 (ITIH2; log_2_FC = −0.64) and follistatin‐like 5 (FSTL5; log_2_FC = −1.00). The ProteoMiner Small‐Capacity Enrichment Kit also showed higher relative enrichment of fibronectin (FN1; log_2_FC = −0.92) and FGB (log_2_FC = −0.37). In contrast, the PreOmics ENRICH‐iST Kit preferentially enriched intracellular and cytoskeletal proteins, including actin beta (ACTB; log_2_FC = 1.44) and nidogen‐2 (NID2; log_2_FC = 2.22), alongside epithelial structural proteins such as keratin‐3 (KRT3; log_2_FC = 1.55) and keratin‐77 (KRT77; log_2_FC = 1.63).

### 3.4 PANTHER Biological Process Pathway Analysis Reveals Broad Cellular and Neuronal Pathway Enrichment Using the PreOmics ENRICH‐iST Kit

In total, 332 pathways were significantly enriched in native digest samples, 284 pathways in samples processed by the ProteoMiner Small‐Capacity Enrichment Kit, and finally, 454 pathways in samples processed by the PreOmics ENRICH‐iST Kit. Of these, 34 pathways were uniquely identified in the native digest samples, 19 pathways in ProteoMiner Small‐Capacity Enrichment samples and 143 in the PreOmics ENRICH‐iST samples. All pathways identified by each method are listed in Supporting Information Table 2.1.

**FIGURE 3:**
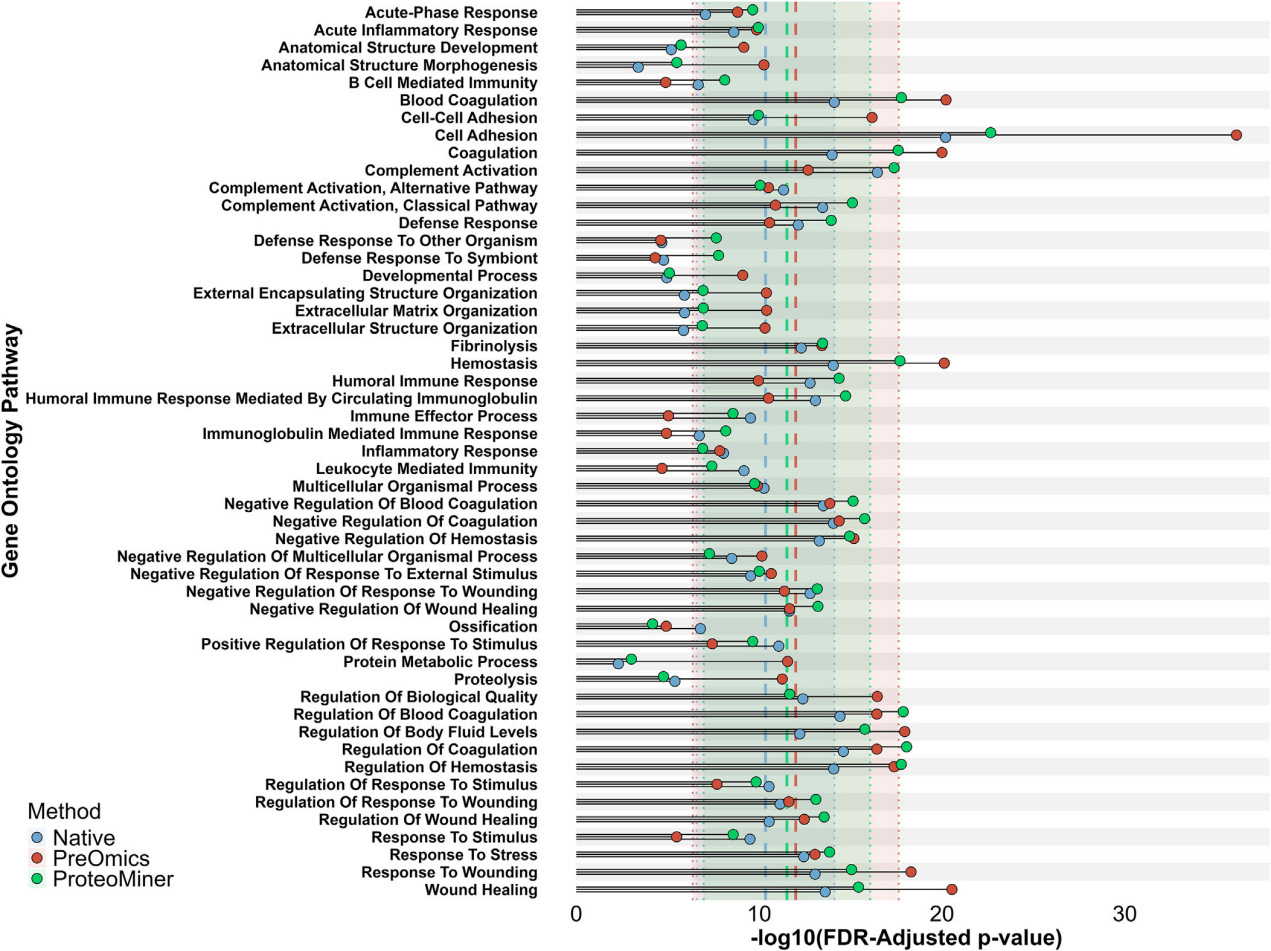
PreOmics ENRICH‐iST demonstrated superior enrichment efficiency, identifying more pathways at greater statistical significance as well as pathways relevant to neuropathologies. Comparison of FDR‐adjusted *p* values (−log_10_ scale) showed the PreOmics ENRICH‐iST Kit (‘PreOmics’, red) generally achieved more significant values across the analysed GO pathways compared to the native digestion (‘Native’, blue) and ProteoMiner Small‐Capacity Enrichment Kit (‘ProteoMiner’, green). Out of the 50 pathways analysed, 22 were identified at a lower FDR by PreOmics ENRICH‐iST. Dashed vertical lines indicate the mean –log_10_(FDR) for each technique (Native: 10.3 ± 3.76; PreOmics: 12.0 ± 5.63; ProteoMiner: 11.5 ± 4.55), with shaded regions representing ± 1 standard deviation and dotted lines marking the boundaries of each distribution. Image made using ggplot2 (version 3.5.2).

Comparing the 50 most significant pathways (ranked by the lowest FDR) from each of the three techniques, PreOmics ENRICH‐iST samples yielded the most significant enrichment with 22 of these 50 pathways enriched at a lower FDR compared to the other methods (Figure 3). Among the most significantly enriched pathways in the PreOmics ENRICH‐iST samples were cell adhesion (FDR = 6.72e^−17^), wound healing (FDR = 2.86e^−21^), hemostasis (FDR = 7.55e^−21^) and blood coagulation (FDR = 6.04e^−21^).

Finally, we compared the unique pathways identified by the ProteoMiner Small‐Capacity Enrichment Kit with the ones uniquely identified by the PreOmics ENRICH‐iST Kit. The ProteoMiner Small‐Capacity Enrichment Kit identified a smaller set of pathways, enriching for synapse‐related and neuronal signalling processes. The most significant neurobiology‐relevant pathways included synapse maturation (FDR = 2.63 × 10e^−^^4^), glutamate receptor clustering (FDR = 2.85 × 10e^−^^2^), AMPA glutamate receptor clustering (FDR = 2.81 × 10e^−^^2^), postsynaptic membrane organisation (FDR = 3.92 × 10e^−^^2^) and postsynapse organisation (FDR = 4.58 × 10e^−^^2^). In addition, pathways related to tissue regeneration (FDR = 3.13 × 10e^−^^2^) and regulation of smooth muscle cell proliferation (FDR = 2.39 × 10e^−^^2^) were identified.

In contrast, the PreOmics ENRICH‐iST Kit identified a substantially larger number of unique pathways. These included pathways associated with neuronal biology, including neuroblast proliferation (FDR = 3.58 × 10e^−^^2^), motor neuron axon guidance (FDR = 3.38 × 10e^−^^2^), positive regulation of nervous system development (FDR = 3.56 × 10e^−^^2^) and brain development (FDR = 6.46 × 10e^−^^3^). Other pathways were regulation of postsynaptic density organisation (FDR = 1.99 × 10e^−^^2^), regulation of postsynaptic membrane neurotransmitter receptor levels (FDR = 2.23 × 10^−^^2^), negative regulation of long‐term synaptic potentiation (FDR = 5.22 × 10e^−^^3^), positive regulation of Rho protein signal transduction (FDR = 2 × 10e^−^^2^) and chaperone‐mediated autophagy (FDR = 4.27 × 10^−^^2^). Pathways uniquely identified by each technique and their FDR values are summarised in Supporting Information Tables 2.2–2.4.

## 4 Discussion

Our pilot study compared the efficiency of the ProteoMiner Small‐Capacity Enrichment Kit and PreOmics ENRICH‐iST Kit in increasing the protein coverage of LC–MS/MS analysed equine CSF. We aimed to identify which technique most effectively depleted HAPs and enriched LAPs relevant to neuropathology, and to evaluate the PreOmics ENRICH‐iST Kit as a viable tool for equine CSF proteomics and neurological biomarker discovery.

All CSF samples were collected immediately post‐mortem, minimising time‐dependent proteolytic and biochemical changes. While post‐mortem CSF may not fully reflect in vivo composition, using the same source material across all enrichment methods ensured consistent comparisons. The PreOmics ENRICH‐iST Kit identified more proteins compared to our native digest control and the ProteoMiner Small‐Capacity Enrichment Kit. The native digest provided a baseline representation of the equine CSF proteome, with a more limited protein coverage and stronger dominance of HAPs such as ALB and TF, highlighting the extent to which enrichment methods altered both proteome depth and the visibility of LAPs. Among the unique protein profiles, the PreOmics ENRICH‐iST Kit identified proteins such as PRDX1, PKM, METRNL, SEMA4D and LMTK3, involved in oxidative stress responses and neuronal energy metabolism (de Geus et al. 2025; Szeliga 2020), synaptic organisation, neuroinflammatory signalling and BBB regulation (Smith et al. 2015), as well as neurodegenerative diseases such as Alzheimer's disease (AD; Leshchyns'ka and Sytnyk 2016; Larose et al. 2024), Parkinson's disease (PD), Huntington's disease (HD) and multiple sclerosis (MS; de Geus et al. 2025; Berghoff et al. 2021; Szeliga 2020; Smith et al. 2015; Larose et al. 2024). ADCY5, known for its role in, ADCY5‐related dyskinesia (Kozon et al. 2024), was also enriched. The identification of these proteins, as well as the ability to capture complex broader proteomic profiles, highlighted the suitability of the PreOmics ENRICH‐iST Kit for studies focusing on equine neurological disease biomarker discovery using equine CSF.

HAPs depletion was a key determinant of LAPs visibility. The PreOmics ENRICH‐iST Kit, but not the ProteoMiner Small‐Capacity Enrichment Kit, substantially decreased the abundance of ALB and TF, along with α2M and CST3. The depletion of HAPs enhanced the identification of a wider range of proteins that would generally not be observed prior to HAPs depletion, further supporting the kit's suitability for in‐depth neurological proteomic studies requiring greater sensitivity. In contrast, samples processed with the ProteoMiner Small‐Capacity Enrichment Kit showed an apparent increase in ALB emPAI values, which likely reflects mass‐spectrometric sampling bias arising from differences in sample matrix composition following enrichment, rather than a true biological increase in ALB abundance.

Proteins uniquely found in enrichment‐processed samples, absent in native digest, were linked to neuroinflammation and tissue damage relevant to EHM, which currently lacks reliable biomarkers. These included FGB, generally found at low abundance under normal physiological conditions, with increased levels associated with neuroinflammation and neurological diseases such as MS, traumatic brain injury, AD, PD and HD (Wen and Zhang 2023) as well as FN1, known indicator of BBB disruption (Petersen et al. 2018; Bhattarai et al. 2025) and subsequent tissue remodelling. Other enriched neurobiology‐relevant LAPs were FBLN2, tied to impairment of myelin production by oligodendrocites (Ghorbani et al. 2024), and PRDX2, a marker of cellular response to oxidative stress (Lu et al. 2018) usually found at low basal levels in healthy CSF, while being the second most abundant protein in CSF of patients affected with traumatic brain injury and subarachnoid haemorrhage (Lu et al. 2018). It is important to note that the supporting evidence for these proteins is largely derived from human literature, as there is currently very limited information linking specific LAPs in equine CSF to neurological diseases. Here, using human disease associations provides translational context. While direct equivalence cannot be assumed, the shared mammalian CNS architecture and conserved protein pathways suggest potential relevance in horses. This approach allows our findings to serve as hypothesis‐generating, guiding future studies for validation in equine neurological disease models.

Further distinctions of each method's results were highlighted by the GO pathway analysis. The PreOmics ENRICH‐iST Kit not only identified the greatest number of significantly enriched pathways, but also provided greater statistical significance, due to the greater number of proteins identified. Among the pathways uniquely identified by the PreOmics ENRICH‐iST Kit, several were directly related to neuronal biology and CNS processes, reflecting core aspects of neural differentiation, connectivity, maturation, as well as synaptic structure and signal transduction. One notably relevant set of pathways uniquely identified by the PreOmics ENRICH‐iST Kit related to axon guidance and synaptic organisation. Axon guidance pathways are essential for maintaining neuronal connectivity and for axonal remodelling following injury, and their dysregulation has been implicated in a range of neuroinflammatory and neurodegenerative conditions (Kolodkin and Tessier‐Lavigne 2011; Van Battum et al. 2015). In the context of EHM, inflammatory signalling within the CNS, including increased interleukin‐6 expression (Zarski et al. 2021), may secondarily perturb neuronal connectivity and synaptic stability. Similarly, alterations in postsynaptic density organisation and neurotransmitter receptor regulation are associated with impaired synaptic transmission during neuroinflammation and CNS injury (O'Reilly and Tom 2020). The detection of proteins mapping to these pathways suggests that the PreOmics ENRICH‐iST Kit may enhance access to biomarkers linked to neuronal dysfunction and repair mechanisms relevant to diseases such as EHM, although targeted comparisons between healthy and EHV‐1‐infected horses would be required to confirm their disease specificity.

This study, however, had limitations that should be considered. First, the sample was derived from a healthy individual, limiting extrapolation to disease states. As such, the ability of enrichment methods to detect clinically relevant disease biomarkers remains to be validated in pathological contexts. Second, with only two technical replicates per method, the robustness of the comparative performance assessments was limited, precluding the use of formal statistical testing. Lastly, protein quantification relied solely on emPAI, which provides semi‐quantitative relative abundance and is primarily designed for within‐sample comparisons, while lacking independent experimental validation. A complementary approach to address this limitation would involve targeted validation using mass spectrometry‐based methods such as multiple reaction monitoring (MRM) or selected reaction monitoring (SRM), which are highly sensitive, quantitative, and species‐agnostic. While western blotting could in principle also be used to validate protein presence and abundance, the scarcity of validated antibodies for equine proteins makes this approach challenging, particularly for low‐abundance CSF proteins. Finally, cost considerations are relevant: the PreOmics ENRICH‐iST Kit offers a streamlined, high‐throughput protocol, but at higher expense, requiring careful balancing of performance, scalability, and financial feasibility.

Future studies could integrate MRM/SRM workflows, which would significantly enhance the confidence and reliability of here presented proteomic data. The development of hybrid workflows that combine the strengths of multiple enrichment methods, as well as validation in antemortem samples, could also provide a practical solution for overcoming the individual limitations of each approach.

## 5 Conclusion

Here, we compared two strategies—the ProteoMiner Small‐Capacity Enrichment Kit and PreOmics ENRICH‐iST Kit—to prepare equine CSF samples for proteomic LC–MS/MS analysis. Our results showed that PreOmics ENRICH‐iST Kit outperformed the ProteoMiner Small‐Capacity Enrichment Kit through better HAPs depletion and superior detection of a broader spectrum of proteins, including biologically relevant LAPs. PreOmics ENRICH‐iST uniquely identified several proteins with roles in neurodevelopment, neurophysiological and pathological processes. Together, these findings suggest that the PreOmics ENRICH‐iST Kit is a powerful tool for equine CSF proteomics and research on neuropathology in horses. Its ability to reliably detect LAPs in CSF might be particularly useful for identifying novel biomarkers of neurological disorders, including EHM.

## Author Contributions

Foti Federico performed the experiments, analysed and interpreted the data, and wrote both the manuscript and the supplement as the main author. Wilson Amie performed the experiments, obtained the CSF sample as well as owning the work as part of her PhD project. Chabronova Alzbeta supervised and assisted with the experiments, aided with data analysis, proofread the manuscript, and provided major contributions to its writing. Jensen Anders wrote the code to plot GO datasets and provided input on data interpretation. Peffers Jayne Mandy performed the mass spectrometry experiments and wrote the relevant methods section, provided a workspace, supervised the experiments, and contributed to proofreading and writing the manuscript. Lunn Paul David secured the funding for the project. All the authors read and approved the final manuscript.

## Funding

This study was supported by a research grant from the Horserace Betting Levy Board (HBLB), whose contribution made this work possible (Grant number vet/prj/811) and Anders Jensen Horserace Betting Levy Board (Prj812).

## Ethics Statement

The use of CSF in this study was approved by the Institute of Infection, Veterinary and Ecological Sciences Ethical Review Board at the University of Liverpool (RETH000553). No additional invasive procedures were performed on live animals, and all protocols adhered to institutional, national, and European regulations, including the Animals (Scientific Procedures) Act 1986.

## Conflicts of Interest

The authors declare no conflicts of interest.

## Supporting information




**Table S1**: Proteins identified by Trypsin/Lys‐C‐based native digestion.
**Table S2**: Proteins identified by ProteoMiner Small‐Capacity Kit.
**Table S3**: Proteins identified by PreOmics ENRICH‐iST Kit.
**Table S4**: Protein overlaps between techniques.
**Table S5**: Pathway overlaps between techniques.
**Table S6**: Pathways unique to native technique.
**Table S7**: Pathways unique to ProteoMiner technique.
**Table S8**: Pathways unique to PreOmics technique.

## Data Availability

The datasets generated and/or analysed during the current study are available in the PRIDE archive (accession: PXD063239), part of the ProteomeXchange Consortium (https://doi.org/10.6019/PXD063239).

## References

[vms370933-bib-0001] Ahmed, S. , P. van Zalm , E. A. Rudmann , et al. 2022. “Using CSF Proteomics to Investigate Herpesvirus Infections of the Central Nervous System.” Viruses 14, no. 12: 2757. 36560759 10.3390/v14122757 PMC9780940

[vms370933-bib-0002] Anderson, J. R. , M. M. Phelan , L. M. Rubio‐Martinez , et al. 2020. “Optimization of Synovial Fluid Collection and Processing for NMR Metabolomics and LC‐MS/MS Proteomics.” Journal of Proteome Research 19, no. 7: 2585–2597. 32227958 10.1021/acs.jproteome.0c00035 PMC7341532

[vms370933-bib-0003] Anderson, N. L. , and A. Ng . 2002. “The Human Plasma Proteome: History, Character, and Diagnostic Prospects.” Molecular & Cellular Proteomics 1, no. 11: 845–867. 12488461 10.1074/mcp.r200007-mcp200

[vms370933-bib-0004] Bardell, D. , P. I. Milner , K. Goljanek‐Whysall , and M. J. Peffers . 2019. “Differences in Plasma and Peritoneal Fluid Proteomes Identifies Potential Biomarkers Associated With Survival Following Strangulating Small Intestinal Disease.” Equine Veterinary Journal 51, no. 6: 727–732. 30854696 10.1111/evj.13094

[vms370933-bib-0005] Berghoff, M. , A. Hopfinger , R. Rajendran , T. Karrasch , A. Schmid , and A Schaffler . 2021. “Evidence of a Muscle‐Brain Axis by Quantification of the Neurotrophic Myokine METRNL (Meteorin‐Like Protein) in Human Cerebrospinal Fluid and Serum.” Journal of Clinical Medicine 10, no. 15: 3271. 34362056 10.3390/jcm10153271 PMC8347672

[vms370933-bib-0006] Bhattarai, P. , E. Yilmaz , E. O. Cakir , et al. 2025. “APOE‐Epsilon4‐Induced Fibronectin at the Blood‐Brain Barrier Is a Conserved Pathological Mediator of Disrupted Astrocyte‐Endothelia Interaction in Alzheimer's Disease.” bioRxiv.

[vms370933-bib-0007] Boschetti, E. , and P. G. Righetti . 2023. “Low‐Abundance Protein Enrichment for Medical Applications: The Involvement of Combinatorial Peptide Library Technique.” International Journal of Molecular Sciences 24, no. 12: 10329. 37373476 10.3390/ijms241210329 PMC10299117

[vms370933-bib-0008] Broccardo, C. J. , G. S. Hussey , L. Goehring , P. Lunn , and J. E. Prenni . 2014. “Proteomic Characterization of Equine Cerebrospinal Fluid.” Journal of Equine Veterinary Science 34, no. 3: 451–458.

[vms370933-bib-0009] Corporation B . 2023. “PreOmics Introduces New ENRICH‐iST Kits and Novel Workflow for Unbiased, Affordable Plasma and Serum Proteomics by LC‐MS.” https://veterinaryresearch.biomedcentral.com/submission‐guidelines/preparing‐your‐manuscript/short‐report.

[vms370933-bib-0010] de Geus, M. B. , P. Kivisakk , B. A. Trombetta , et al. 2025. “Immunoassay for Pyruvate Kinase M1/2 as an Alzheimer's Biomarker in CSF.” Open Life Sciences 20, no. 1: 20251101. 40667488 10.1515/biol-2025-1101 PMC12260348

[vms370933-bib-0011] Donnelly, C. G. , A. L. Johnson , S. Reed , and C. J. Finno . 2023. “Cerebrospinal Fluid and Serum Proteomic Profiles Accurately Distinguish Neuroaxonal Dystrophy From Cervical Vertebral Compressive Myelopathy in Horses.” Journal of Veterinary Internal Medicine 37, no. 2: 689–696. 36929645 10.1111/jvim.16660 PMC10061172

[vms370933-bib-0012] Frischknecht, R. , and C. I. Seidenbecher . 2012. “Brevican: A Key Proteoglycan in the Perisynaptic Extracellular Matrix of the Brain.” International Journal of Biochemistry & Cell Biology 44, no. 7: 1051–1054. 22537913 10.1016/j.biocel.2012.03.022

[vms370933-bib-0013] Gao, C. D. A. 2024. “ggVennDiagram: A ‘ggplot2’ Implement of Venn Diagram. R Package Version 1.5.2.” https://CRAN.R‐project.org/package=ggVennDiagram.

[vms370933-bib-0014] Ghorbani, S. , C. Li , and B. M. Lozinski , et al. 2024. “Fibulin‐2 Is an Extracellular Matrix Inhibitor of Oligodendrocytes Relevant to Multiple Sclerosis.” Journal of Clinical Investigation 134, no. 13: e176910. 38743490 10.1172/JCI176910 PMC11213512

[vms370933-bib-0015] Gutierrez‐Franco, A. , H. Eixarch , C. Costa , et al. 2017. “Semaphorin 7A as a Potential Therapeutic Target for Multiple Sclerosis.” Molecular Neurobiology 54, no. 6: 4820–4831. 27714632 10.1007/s12035-016-0154-2

[vms370933-bib-0016] Ishihama, Y. , Y. Oda , T. Tabata , et al. 2005. “Exponentially Modified Protein Abundance Index (emPAI) for Estimation of Absolute Protein Amount in Proteomics by the Number of Sequenced Peptides per Protein.” Molecular & Cellular Proteomics 4, no. 9: 1265–1272. 15958392 10.1074/mcp.M500061-MCP200

[vms370933-bib-0017] Jankovska, E. , M. Svitek , K. Holada , and J. Petrak . 2019. “Affinity Depletion Versus Relative Protein Enrichment: A Side‐by‐Side Comparison of Two Major Strategies for Increasing Human Cerebrospinal Fluid Proteome Coverage.” Clinical Proteomics 16: 9. 30890900 10.1186/s12014-019-9229-1 PMC6390343

[vms370933-bib-0018] Johnson, B. B. , M. V. Cosson , L. I. Tsansizi , et al. 2024. “Perlecan (HSPG2) Promotes Structural, Contractile, and Metabolic Development of Human Cardiomyocytes.” Cell Reports 43, no. 1: 113668. 38198277 10.1016/j.celrep.2023.113668

[vms370933-bib-0019] Kolodkin, A. L. , and M. Tessier‐Lavigne . 2011. “Mechanisms and Molecules of Neuronal Wiring: A Primer.” Cold Spring Harbor Perspectives in Biology 3, no. 6: a001727. 21123392 10.1101/cshperspect.a001727 PMC3098670

[vms370933-bib-0020] Kozon, K. , W. Lysikowska , J. Olszewski , et al. 2024. “ADCY5‐Related Dyskinesia—Case Series With Literature Review.” Neurologia Neurochirurgia Polska 58, no. 2: 161–166. 10.5603/pjnns.97024 38230756

[vms370933-bib-0021] Kroksveen, A. C. , J. A. Opsahl , T. T. Aye , R. J. Ulvik , and F. S. Berven . 2011. “Proteomics of Human Cerebrospinal Fluid: Discovery and Verification of Biomarker Candidates in Neurodegenerative Diseases Using Quantitative Proteomics.” Journal of Proteomics 74, no. 4: 371–388. 21111852 10.1016/j.jprot.2010.11.010

[vms370933-bib-0022] Larose, A. , C. C. J. Miller , and G. M. Morotz . 2024. “The Lemur Tail Kinase Family in Neuronal Function and Disfunction in Neurodegenerative Diseases.” Cellular and Molecular Life Sciences 81, no. 1: 447. 39520508 10.1007/s00018-024-05480-0 PMC11550312

[vms370933-bib-0023] Leshchyns'ka, I. , and V. Sytnyk . 2016. “Synaptic Cell Adhesion Molecules in Alzheimer's Disease.” Neural Plasticity 2016: 6427537. 27242933 10.1155/2016/6427537 PMC4868906

[vms370933-bib-0024] Li, J. , F. Dai , X. Kou , B. Wu , J. Xu , and S. He . 2023. “beta‐Actin: An Emerging Biomarker in Ischemic Stroke.” Cellular and Molecular Neurobiology 43, no. 2: 683–696. 35556192 10.1007/s10571-022-01225-4 PMC11415192

[vms370933-bib-0025] Li, L. 2015. “Dynamic Range Compression With ProteoMiner: Principles and Examples.” Methods in Molecular Biology 1295: 99–107. 25820717 10.1007/978-1-4939-2550-6_9

[vms370933-bib-0026] Lu, Y. , X. S. Zhang , Z. H. Zhang , et al. 2018. “Peroxiredoxin 2 Activates Microglia by Interacting With Toll‐Like Receptor 4 After Subarachnoid Hemorrhage.” Journal of Neuroinflammation 15, no. 1: 87. 29554978 10.1186/s12974-018-1118-4 PMC5859544

[vms370933-bib-0027] Lukasik, P. , M. Zaluski , and I. Gutowska . 2021. “Cyclin‐Dependent Kinases (CDK) and Their Role in Diseases Development – Review.” International Journal of Molecular Sciences 22, no. 6: 2935. 33805800 10.3390/ijms22062935 PMC7998717

[vms370933-bib-0028] Lunn, D. P. , B. A. Burgess , D. C. Dorman , et al. 2024. “Updated ACVIM Consensus Statement on Equine Herpesvirus‐1.” Journal of Veterinary Internal Medicine 38, no. 3: 1290–1299. 38497217 10.1111/jvim.17047 PMC11099706

[vms370933-bib-0029] Martins‐De‐Souza, D. , T. Wobrock , I. Zerr , et al. 2010. “Different Apolipoprotein E, Apolipoprotein A1 and Prostaglandin‐H2 D‐Isomerase Levels in Cerebrospinal Fluid of Schizophrenia Patients and Healthy Controls.” World Journal of Biological Psychiatry 11, no. 5: 719–728. 10.3109/15622971003758748 20446881

[vms370933-bib-0030] Mi, H. , A. Muruganujan , and P. D. Thomas . 2013. “PANTHER in 2013: Modeling the Evolution of Gene Function, and Other Gene Attributes, in the Context of Phylogenetic Trees.” Nucleic Acids Research 41: D377–D386. 23193289 10.1093/nar/gks1118 PMC3531194

[vms370933-bib-0031] Nunez Galindo, A. , M. Kussmann , and L. Dayon . 2015. “Proteomics of Cerebrospinal Fluid: Throughput and Robustness Using a Scalable Automated Analysis Pipeline for Biomarker Discovery.” Analytical Chemistry 87, no. 21: 10755–10761. 26452177 10.1021/acs.analchem.5b02748

[vms370933-bib-0032] Ogata, H. , R. Yamasaki , A. Hiwatashi , et al. 2015. “Characterization of IgG4 Anti‐Neurofascin 155 Antibody‐Positive Polyneuropathy.” Annals of Clinical and Translational Neurology 2, no. 10: 960–971. 26478896 10.1002/acn3.248 PMC4603379

[vms370933-bib-0033] O'Reilly, M. L. , and V. J. Tom . 2020. “Neuroimmune System as a Driving Force for Plasticity Following CNS Injury.” Frontiers in Cellular Neuroscience 14: 187. 32792908 10.3389/fncel.2020.00187 PMC7390932

[vms370933-bib-0034] Peffers, M. J. , C. T. Thorpe , J. A. Collins , et al. 2014. “Proteomic Analysis Reveals Age‐Related Changes in Tendon Matrix Composition, With Age‐ and Injury‐Specific Matrix Fragmentation.” Journal of Biological Chemistry 289, no. 37: 25867–25878. 25077967 10.1074/jbc.M114.566554 PMC4162187

[vms370933-bib-0035] Petersen, M. A. , J. K. Ryu , and K. Akassoglou . 2018. “Fibrinogen in Neurological Diseases: Mechanisms, Imaging and Therapeutics.” Nature Reviews Neuroscience 19, no. 5: 283–301. 29618808 10.1038/nrn.2018.13 PMC6743980

[vms370933-bib-0036] Martin Flores, N. , M. Podpolny , F. McLeod , et al. 2024. “Downregulation of Dickkopf‐3, a Wnt Antagonist Elevated in Alzheimer's Disease, Restores Synapse Integrity and Memory in a Disease Mouse Model.” Elife 12: RP89453. 38285009 10.7554/eLife.89453 PMC10945611

[vms370933-bib-0037] Seeliger, T. , S. Gingele , Y. E. Guzeloglu , et al. 2023. “Comparative Analysis of Albumin Quotient and Total CSF Protein in Immune‐Mediated Neuropathies: A Multicenter Study on Diagnostic Implications.” Frontiers in Neurology 14: 1330484. 38264088 10.3389/fneur.2023.1330484 PMC10803547

[vms370933-bib-0038] Smith, E. S. , A. Jonason , C. Reilly , et al. 2015. “SEMA4D Compromises Blood‐Brain Barrier, Activates Microglia, and Inhibits Remyelination in Neurodegenerative Disease.” Neurobiology of Disease 73: 254–268. 25461192 10.1016/j.nbd.2014.10.008

[vms370933-bib-0039] Szeliga, M. 2020. “Peroxiredoxins in Neurodegenerative Diseases.” Antioxidants (Basel) 9, no. 12: 1203. 33265993 10.3390/antiox9121203 PMC7761365

[vms370933-bib-0040] Team RC . “R: A Language and Environment for Statistical Computing.” 2021. https://www.R‐project.org/.

[vms370933-bib-0041] Van Battum, E. Y. , S. Brignani , and R. J. Pasterkamp . 2015. “Axon Guidance Proteins in Neurological Disorders.” Lancet Neurology 14, no. 5: 532–546. 25769423 10.1016/S1474-4422(14)70257-1

[vms370933-bib-0042] Wen, T. , and Z. Zhang . 2023. “Cellular Mechanisms of Fibrin (ogen): Insight From Neurodegenerative Diseases.” Frontiers in Neuroscience 17: 1197094. 37529232 10.3389/fnins.2023.1197094 PMC10390316

[vms370933-bib-0043] Wickham, H. 2016. ggplot2: Elegant Graphics for Data Analysis. Springer‐Verlag. https://ggplot2.tidyverse.org.

[vms370933-bib-0044] Wu, C. , J. Duan , T. Liu , R. D. Smith , and W. J. Qian . 2016. “Contributions of Immunoaffinity Chromatography to Deep Proteome Profiling of Human Biofluids.” Journal of Chromatography B, Analytical Technologies in the Biomedical and Life Sciences 1021: 57–68. 26868616 10.1016/j.jchromb.2016.01.015 PMC4862894

[vms370933-bib-0045] Xu, L. , A. Nirwane , and Y. Yao . 2019. “Basement Membrane and Blood‐Brain Barrier.” Stroke and Vascular Neurology 4, no. 2: 78–82. 31338215 10.1136/svn-2018-000198 PMC6613871

[vms370933-bib-0046] Zarski, L. M. , K. S. Giessler , S. I. Jacob , et al. 2021. “Identification of Host Factors Associated With the Development of Equine Herpesvirus Myeloencephalopathy by Transcriptomic Analysis of Peripheral Blood Mononuclear Cells From Horses.” Viruses 13, no. 3: 356. 33668216 10.3390/v13030356 PMC7995974

