# Supplementary material for: Evaluating Protein Enrichment Methods to Improve Biomarker Discovery in Equine Cerebrospinal Fluid
Source: Vet Med Sci. 2026 Apr 10;12(3):e70933. doi: 10.1002/vms3.70933 (PMC13067976; doi:10.1002/vms3.70933)
Supplement: Supplementary file 1 — Table S1: Proteins identified by Trypsin/Lys‐C‐based native digestion. Table S2: Proteins identified by ProteoMiner Small‐Capacity Kit. Table S3: Proteins identified by PreOmics ENRICH‐iST Kit. Table S4: Protein overlaps between techniques. Table S5: Pathway overlaps between techniques. Table S6: Pathways unique to native technique. Table S7: Pathways unique to ProteoMiner technique. Table S8: Pathways unique to PreOmics technique. [file VMS3-12-e70933-s001.docx]

# Supplementary Tables

## Table S1. Proteins Identified by Trypsin/Lys-C-Based Native Digestion

Total proteins: 382

| **Protein Name** | **Gene Name** | **Mean emPAI ± SD** |
| --- | --- | --- |
| 14-3-3 protein theta | YWHAQ | 0.305 ± 0.007 |
| Acetylcholinesterase (Cartwright blood group) | ACHE | 0.05 ± 0 |
| Activated leukocyte cell adhesion molecule | ALCAM | 0.06 ± 0 |
| ADAM metallopeptidase domain 22 | ADAM22 | 0.11 ± 0 |
| Adhesion G protein-coupled receptor L1 | ADGRL1 | 0.06 ± 0.014 |
| Adhesion G protein-coupled receptor V1 | ADGRV1 | 0.01 ± 0 |
| Adiponectin A | C1QB | 0.42 ± 0.141 |
| Adiponectin D | ADIPOQ | 0.12 ± 0 |
| AE binding protein 1 | AEBP1 | 0.14 ± 0.071 |
| Afamin | AFM | 1.99 ± 0.113 |
| Agrin | AGRN | 0.05 ± 0 |
| Albumin | ALB | 14.78 ± 0.537 |
| Alpha-1-antiproteinase 2 | N/A | 2.735 ± 0.205 |
| Alpha-1-antitrypsin | SPI2 | 3.28 ± 0.948 |
| Alpha-1-B glycoprotein | A1BG | 2.555 ± 0.163 |
| Alpha-2-glycoprotein 1, zinc-binding | AZGP1 | 0.71 ± 0.113 |
| Alpha-2-HS-glycoprotein | AHSG | 1.59 ± 0 |
| Alpha-2-macroglobulin | N/A | 0.26 ± 0 |
| Alpha-2-macroglobulin | A2M | 0.945 ± 0.134 |
| Alpha-amylase | LOC100049851 | 0.795 ± 0.007 |
| Alpha-L-fucosidase | FUCA1 | 0.07 ± 0 |
| Alpha-mannosidase | MAN2A2 | 0.095 ± 0.021 |
| Amine oxidase | AOC3 | 0.34 ± 0.042 |
| Amyloid beta like protein 1 | APLP1 | 1.495 ± 0.092 |
| Amyloid beta like protein 2 | APLP2 | 0.98 ± 0.141 |
| Amyloid beta precursor like protein 2 | LOC111767432 | 0.66 ± 0.057 |
| Amyloid-beta A4 protein | APP | 1.785 ± 0.007 |
| Anaphylatoxin-like domain-containing protein | N/A | 1.65 ± 0.707 |
| Angiotensin-converting enzyme | ACE | 0.05 ± 0 |
| Angiotensinogen | AGT | 2.355 ± 0.332 |
| Antithrombin-III | SERPINC1 | 3.955 ± 0.233 |
| Apolipoprotein A-II | APOA2 | 4.845 ± 1.294 |
| Apolipoprotein C-II | APOC2 | 0.645 ± 0.375 |
| Apolipoprotein C-III | APOC3 | 0.78 ± 0 |
| Apolipoprotein D | APOD | 1.315 ± 0.247 |
| Apolipoprotein E | APOA1 | 10.155 ± 1.011 |
| Apolipoprotein E | APOA4 | 5.805 ± 0.021 |
| Apolipoprotein E | APOE | 11.48 ± 0.905 |
| ArfGAP with SH3 domain, ankyrin repeat and PH domain 2 | ASAP2 | 0.03 ± 0 |
| Aspartate aminotransferase | GOT1 | 0.195 ± 0.148 |
| ATP binding cassette subfamily C member 13 | ABCC13 | 0.03 ± 0 |
| ATPase H+ transporting accessory protein 1 | ATP6AP1 | 0.24 ± 0 |
| ATP-dependent RNA helicase | DDX55 | 0.06 ± 0 |
| Attractin | ATRN | 0.02 ± 0 |
| Beta-1,3-N-acetylglucosaminyltransferase | LFNG | 0.1 ± 0 |
| Beta-1,4-galactosyltransferase | B4GALT1 | 0.08 ± 0 |
| Beta-1,4-glucuronyltransferase 1 | B4GAT1 | 1.485 ± 0.134 |
| Beta-2-glycoprotein 1 | APOH | 1.455 ± 0.163 |
| Beta-2-microglobulin | B2M | 1.49 ± 0.453 |
| Biotinidase | BTD | 0.06 ± 0 |
| BPI fold containing family A member 2 | BPIFA2 | 2.015 ± 0.007 |
| Brevican | BCAN | 0.855 ± 0.049 |
| Cache domain containing 1 | CACHD1 | 0.03 ± 0 |
| Cadherin 13 | CDH13 | 0.62 ± 0 |
| Cadherin 2 | CDH2 | 0.3 ± 0 |
| Cadherin EGF LAG seven-pass G-type receptor 2 | CELSR2 | 0.01 ± 0 |
| Calcium activated nucleotidase 1 | CANT1 | 0.215 ± 0.064 |
| Calcium voltage-gated channel auxiliary subunit alpha2delta 1 | CACNA2D1 | 0.11 ± 0.028 |
| calcium/calmodulin-dependent protein kinase | CAMK2A | 0.23 ± 0 |
| Calreticulin | CALR | 0.08 ± 0 |
| Calsyntenin 1 | CLSTN1 | 0.895 ± 0.049 |
| Calsyntenin 3 | CLSTN3 | 0.04 ± 0 |
| Carboxylic ester hydrolase | LOC100050764 | 0.21 ± 0 |
| Carboxylic ester hydrolase | LOC100050992 | 0.13 ± 0 |
| Carboxypeptidase B2 | CPB2 | 0.36 ± 0 |
| Carboxypeptidase E | CPE | 2.305 ± 0.007 |
| Carboxypeptidase M | CPM | 0.08 ± 0 |
| Cartilage acidic protein 1 | CRTAC1 | 1.305 ± 0.177 |
| Cathepsin B | CTSB | 0.3 ± 0 |
| Cathepsin D | CTSD | 0.735 ± 0.078 |
| Cathepsin F | CTSF | 0.17 ± 0.127 |
| Cathepsin V | CTSV | 1.22 ± 0.311 |
| C-C motif chemokine | LOC100630171 | 0.57 ± 0 |
| CD44 antigen | CD44 | 0.19 ± 0 |
| CD5 molecule like | CD5L | 0.27 ± 0 |
| Cell adhesion molecule 1 | CADM1 | 0.4 ± 0.099 |
| Cell adhesion molecule 3 | CADM3 | 0.18 ± 0 |
| Cell adhesion molecule L1 like | CHL1 | 0.915 ± 0.035 |
| Cellular communication network factor 3 | CCN3 | 0.58 ± 0.198 |
| Centromere protein H | CENPH | 0.15 ± 0 |
| Centrosomal protein of 162 kDa | CEP162 | 0.02 ± 0 |
| Ceramide kinase like | CERKL | 0.06 ± 0 |
| Ceruloplasmin | CP | 1.92 ± 0.311 |
| Cholecystokinin | CCK | 0.35 ± 0 |
| Chromogranin A | CHGA | 3.67 ± 0.014 |
| Chromogranin B | CHGB | 1.005 ± 0.106 |
| Chromosome 13 C16orf89 homolog | C13H16orf89 | 0.58 ± 0 |
| Chromosome 4 open reading frame 48 | C4orf48 | 1.81 ± 0 |
| Clusterin | CLU | 3.845 ± 0.262 |
| Coagulation factor X | F10 | 0.19 ± 0.057 |
| Coagulation factor XII | F12 | 0.24 ± 0 |
| Cochlin | COCH | 0.095 ± 0.049 |
| Coiled-coil domain containing 110 | CCDC110 | 0.04 ± 0 |
| Coiled-coil domain containing 138 | CCDC138 | 0.05 ± 0 |
| Collagen type I alpha 1 chain | COL1A1 | 0.28 ± 0.028 |
| Collagen type I alpha 2 chain | COL1A2 | 0.175 ± 0.021 |
| Collagen type VI alpha 1 chain | COL6A1 | 0.04 ± 0 |
| Collagen type XII alpha 1 chain | COL12A1 | 0.02 ± 0.014 |
| Collagen type XVIII alpha 1 chain | COL18A1 | 0.08 ± 0 |
| Complement C1q C chain | C1QC | 0.47 ± 0 |
| Complement C1r | C1R | 0.55 ± 0.057 |
| Complement C1s | C1S | 0.625 ± 0.106 |
| Complement C2 | C2 | 0.82 ± 0.057 |
| Complement C2 | CFB | 2.225 ± 0.007 |
| Complement C3 | LOC100060505 | 4.515 ± 0.092 |
| Complement C5 | C5 | 0.49 ± 0.042 |
| Complement C7 | C7 | 0.67 ± 0 |
| Complement C8 alpha chain | C8A | 0.4 ± 0 |
| Complement C8 beta chain | C8B | 0.215 ± 0.049 |
| Complement C8 gamma chain | C8G | 0.285 ± 0.148 |
| Complement C9 | C9 | 1.17 ± 0 |
| Complement component 4 binding protein alpha | C4BPA | 0.35 ± 0.127 |
| Complement component C6 | C6 | 0.11 ± 0 |
| Complement factor D | CFD | 1.045 ± 0.12 |
| Complement factor I | CFI | 1.3 ± 0.17 |
| Complement factor properdin | CFP | 0.07 ± 0 |
| Contactin 2 | CNTN2 | 0.49 ± 0 |
| Contactin-1 | CNTN1 | 1.225 ± 0.049 |
| Cryptochrome circadian regulator 1 | CRY1 | 0.06 ± 0 |
| C-type lectin domain containing 11A | CLEC11A | 0.23 ± 0 |
| C-type lectin domain family 3 member B | CLEC3B | 1.275 ± 0.205 |
| CutA divalent cation tolerance homolog | CUTA | 0.49 ± 0.269 |
| Cystatin C | CST3 | 2.3 ± 0 |
| Cystatin E/M | CST6 | 1.46 ± 0 |
| Cysteine rich secretory protein LCCL domain containing 2 | CRISPLD2 | 0.07 ± 0 |
| Cytokine like 1 | CYTL1 | 0.27 ± 0 |
| DDB1 and CUL4 associated factor 1 | DCAF1 | 0.02 ± 0 |
| Decorin | DCN | 0.35 ± 0 |
| Delta/notch like EGF repeat containing | DNER | 0.075 ± 0.035 |
| dipeptidyl-peptidase I | CTSC | 0.16 ± 0.042 |
| DNA topoisomerase I | TOP1MT | 0.03 ± 0 |
| Dystroglycan 1 | DAG1 | 0.31 ± 0.071 |
| ECRG4 augurin | ECRG4 | 0.2 ± 0 |
| Ectonucleotide pyrophosphatase/phosphodiesterase 2 | ENPP2 | 0.895 ± 0.049 |
| EGF containing fibulin extracellular matrix protein 1 | EFEMP1 | 1.2 ± 0.099 |
| EGF containing fibulin extracellular matrix protein 2 | EFEMP2 | 0.29 ± 0 |
| Exostosin like glycosyltransferase 2 | EXTL2 | 0.285 ± 0.092 |
| Extracellular matrix protein 1 | ECM1 | 1.075 ± 0.177 |
| FAT atypical cadherin 2 | FAT2 | 0.085 ± 0.007 |
| Fetuin B | FETUB | 3.245 ± 0.007 |
| Fibrinogen alpha chain | FGA | 1.59 ± 0.071 |
| Fibrinogen alpha chain (Fragment) | FGA | 5.33 ± 0 |
| Fibrinogen gamma chain | FGG | 2.465 ± 0.177 |
| Fibulin 5 | FBLN5 | 0.66 ± 0 |
| Fibulin-1 | FBLN1 | 0.69 ± 0.113 |
| folate gamma-glutamyl hydrolase | GGH | 0.32 ± 0.325 |
| Follistatin like 1 | FSTL1 | 0.1 ± 0.014 |
| Fructose-bisphosphate aldolase | ALDOB | 0.22 ± 0.014 |
| FYVE, RhoGEF and PH domain containing 3 | FGD3 | 0.13 ± 0.113 |
| G protein pathway suppressor 1 | GPS1 | 0.085 ± 0.049 |
| G protein-coupled receptor 37 | GPR37 | 0.065 ± 0.007 |
| G protein-coupled receptor 37 like 1 | GPR37L1 | 0.095 ± 0.035 |
| Galectin-3-binding protein | LGALS3BP | 0.125 ± 0.092 |
| Gastrin releasing peptide | GRP | 0.275 ± 0.007 |
| GDNF family receptor alpha-2 | GFRA2 | 0.215 ± 0.092 |
| Gelsolin | GSN | 0.865 ± 0.757 |
| Globin family profile domain-containing protein | LOC100068926 | 0.9 ± 1.004 |
| glutaminyl-peptide cyclotransferase | QPCT | 0.14 ± 0.071 |
| Glutathione peroxidase | GPX3 | 0.44 ± 0.495 |
| GM2 activator protein | GM2A | 1.49 ± 1.329 |
| Golgi membrane protein 1 | GOLM1 | 1.67 ± 1.089 |
| Guanine deaminase | GDA | 0.455 ± 0.417 |
| Haptoglobin | LOC100067869 | 3.325 ± 4.589 |
| Hedgehog protein | SHH | 3.34 ± 4.61 |
| Hematopoietic SH2 domain containing | HSH2D | 0.09 ± 0.014 |
| Hemopexin | HPX | 1.835 ± 2.454 |
| Heparan sulfate proteoglycan 2 | HSPG2 | 1.97 ± 2.772 |
| Histidine rich glycoprotein | HRG | 1.35 ± 1.895 |
| HtrA serine peptidase 1 | HTRA1 | 1.55 ± 2.008 |
| Hyaluronan binding protein 2 | HABP2 | 0.22 ± 0.127 |
| IF rod domain-containing protein | N/A | 0.32 ± 0.141 |
| IF rod domain-containing protein | KRT10B | 0.76 ± 0.82 |
| IF rod domain-containing protein | KRT2 | 0.675 ± 0.361 |
| Ig-like domain-containing protein | N/A | 1.52 ± 0.948 |
| ILEI/PANDER domain-containing protein | N/A | 3.055 ± 0.785 |
| Immunoglobulin superfamily member 8 | IGSF8 | 0.06 ± 0 |
| Immunoglobulin V-set domain-containing protein | N/A | 0.2 ± 0 |
| Inducible T cell costimulator ligand | ICOSLG | 0.27 ± 0 |
| Insulin like growth factor binding protein 4 | IGFBP4 | 0.14 ± 0 |
| Insulin like growth factor binding protein 6 | IGFBP6 | 0.795 ± 0.361 |
| Insulin like growth factor binding protein 7 | IGFBP7 | 0.45 ± 0 |
| Insulin-like growth factor II | IGF2 | 0.17 ± 0 |
| Insulin-like growth factor-binding protein 2 | IGFBP2 | 3.81 ± 0.014 |
| Integral membrane protein 2 | ITM2B | 0.49 ± 0 |
| Integrin subunit beta like 1 | ITGBL1 | 0.12 ± 0 |
| Inter-alpha-trypsin inhibitor heavy chain 1 | ITIH1 | 0.69 ± 0.085 |
| Inter-alpha-trypsin inhibitor heavy chain 4 | ITIH4 | 1.49 ± 0.141 |
| Inter-alpha-trypsin inhibitor heavy chain 5 | ITIH5 | 0.11 ± 0 |
| Intercellular adhesion molecule 5 | ICAM5 | 0.145 ± 0.092 |
| Interleukin 1 receptor accessory protein | IL1RAP | 0.325 ± 0.049 |
| Interleukin 12 receptor subunit beta 2 | IL12RB2 | 0.04 ± 0 |
| Intraflagellar transport 74 | IFT74 | 0.06 ± 0 |
| Joining chain of multimeric IgA and IgM | JCHAIN | 0.835 ± 0.007 |
| Kallikrein B1 | KLKB1 | 0.11 ± 0 |
| Kallikrein related peptidase 6 | KLK6 | 1.135 ± 0.007 |
| Kazal type serine peptidase inhibitor domain 1 | KAZALD1 | 0.12 ± 0 |
| Keratin 15 | KRT15 | 0.53 ± 0.085 |
| Keratin 6C | KRT85 | 0.89 ± 0.735 |
| Keratin 72 | KRT72 | 0.38 ± 0 |
| Keratin 80 | KRT80 | 0.17 ± 0 |
| Keratin, type II cytoskeletal 1 | KRT1 | 0.225 ± 0.049 |
| KIAA1549 like | KIAA1549L | 0.065 ± 0.021 |
| Kininogen 1 | KNG1 | 1.28 ± 0.17 |
| Lactotransferrin | LTF | 0.21 ± 0 |
| Latent transforming growth factor beta binding protein 4 | LTBP4 | 0.03 ± 0.014 |
| Lectin, mannose binding 2 | LMAN2 | 0.485 ± 0.092 |
| Leucine rich alpha-2-glycoprotein 1 | LRG1 | 1.655 ± 0.007 |
| Leucine rich repeat and Ig domain containing 1 | LINGO1 | 0.11 ± 0.085 |
| Limbic system associated membrane protein | LSAMP | 1.19 ± 0.297 |
| Lipocalin/cytosolic fatty-acid binding domain-containing protein | LOC100051562 | 1.65 ± 0.325 |
| Lipocalin/cytosolic fatty-acid binding domain-containing protein | LOC100068210 | 0.2 ± 0 |
| Lipopolysaccharide-binding protein | LBP | 0.195 ± 0.064 |
| L-lactate dehydrogenase | LDHA | 0.215 ± 0.163 |
| L-lactate dehydrogenase | LDHB | 0.3 ± 0.156 |
| Lumican | LUM | 0.79 ± 0.24 |
| Ly6/neurotoxin 1 | LYNX1 | 0.26 ± 0 |
| Lymphocyte antigen 6 family member H | LY6H | 0.14 ± 0 |
| Lysocardiolipin acyltransferase 1 | LCLAT1 | 0.09 ± 0 |
| Major prion protein | PRNP | 0.835 ± 0.177 |
| Maltase-glucoamylase | MGAM | 0.03 ± 0.014 |
| Mast/stem cell growth factor receptor Kit | KIT | 0.03 ± 0 |
| Matrix Gla protein | MGP | 0.79 ± 0 |
| Matrix metallopeptidase 17 | MMP17 | 0.05 ± 0 |
| Matrix metallopeptidase 2 | MMP2 | 0.99 ± 0.212 |
| MBL associated serine protease 1 | MASP1 | 0.1 ± 0 |
| Membrane cofactor protein | LOC100057176 | 0.305 ± 0.007 |
| Metalloproteinase inhibitor 1 | TIMP1 | 0.485 ± 0.163 |
| Microfibril associated protein 4 | MFAP4 | 0.545 ± 0.134 |
| Monocyte differentiation antigen CD14 | CD14 | 0.68 ± 0.113 |
| Mucin 4, cell surface associated | MUC4 | 0.03 ± 0 |
| Multiple inositol polyphosphate phosphatase 1 | MINPP1 | 0.65 ± 0 |
| Myocilin | MYOC | 0.11 ± 0.057 |
| N-acetyllactosaminide alpha-1,3-galactosyltransferase | LOC100067589 | 0.06 ± 0 |
| Neogenin 1 | NEO1 | 0.065 ± 0.021 |
| Neural cell adhesion molecule 1 | NCAM1 | 0.73 ± 0.042 |
| Neural cell adhesion molecule 2 | NCAM2 | 0.13 ± 0 |
| Neural EGFL like 2 | NELL2 | 1.335 ± 0.064 |
| Neural proliferation, differentiation and control 1 | NPDC1 | 0.11 ± 0 |
| Neurexin 1 | NRXN1 | 0.145 ± 0.035 |
| Neurexin 2 | NRXN2 | 0.135 ± 0.021 |
| Neurexin 3 | NRXN3 | 0.215 ± 0.049 |
| Neurexophilin | NXPH1 | 0.14 ± 0 |
| Neuritin 1 | NRN1 | 0.27 ± 0 |
| Neuroblastoma suppressor of tumorigenicity 1 | NBL1 | 0.75 ± 0 |
| Neurocan | NCAN | 0.375 ± 0.021 |
| Neuroendocrine protein 7B2 | SCG5 | 1.03 ± 0.226 |
| Neurofascin | NFASC | 0.135 ± 0.064 |
| Neuronal cell adhesion molecule | NRCAM | 1.33 ± 0.113 |
| Neuronal growth regulator 1 | NEGR1 | 1.15 ± 0 |
| Neuronal pentraxin 1 | NPTX1 | 1.485 ± 0.134 |
| Neuronal pentraxin 2 | NPTX2 | 0.125 ± 0.064 |
| Neuronal pentraxin receptor | NPTXR | 1.595 ± 0.134 |
| Neuronal vesicle trafficking associated 1 | NSG1 | 0.1 ± 0 |
| Neuropeptide Y | NPY | 0.4 ± 0 |
| Neurotrimin | NTM | 1.035 ± 0.134 |
| Nidogen 1 | NID1 | 0.105 ± 0.021 |
| non-specific serine/threonine protein kinase | MAPKAPK5 | 0.07 ± 0 |
| NPC intracellular cholesterol transporter 2 | NPC2 | 2.205 ± 0.007 |
| NTR domain-containing protein | LOC100060539 | 3.52 ± 0.679 |
| Nucleobindin 1 | NUCB1 | 0.375 ± 0.064 |
| Olfactomedin 1 | OLFM1 | 0.11 ± 0.057 |
| Olfactory receptor family 2 subfamily T member 68 | OR2T68 | 0.11 ± 0 |
| Oligodendrocyte myelin glycoprotein | OMG | 0.69 ± 0 |
| Opioid binding protein/cell adhesion molecule like | OPCML | 0.91 ± 0.396 |
| Ornithine decarboxylase 1 | ODC1 | 0.15 ± 0 |
| Osteoglycin | OGN | 1.53 ± 0.198 |
| Out at first protein homolog | POU2F3 | 0.09 ± 0 |
| Paraoxonase | PON1 | 0.76 ± 0.226 |
| Peptidase inhibitor 16 | PI16 | 0.255 ± 0.134 |
| Peptidylglycine alpha-amidating monooxygenase | PAM | 0.52 ± 0 |
| Phosphatidylethanolamine binding protein 1 | PEBP1 | 1.66 ± 0.325 |
| Phosphatidylethanolamine binding protein 4 | PEBP4 | 0.77 ± 0 |
| Phosphoinositide-3-kinase interacting protein 1 | PIK3IP1 | 0.22 ± 0 |
| Phospholipase D family member 3 | PLD3 | 0.31 ± 0 |
| Phospholipid transfer protein | PLTP | 1.035 ± 0.148 |
| Phospholipid-transporting ATPase | ATP11C | 0.03 ± 0 |
| Plasma retinol-binding protein | LOC111773844 | 0.565 ± 0.092 |
| Plasminogen | PLG | 2.57 ± 0.099 |
| Pleckstrin homology domain containing N1 | PLEKHN1 | 0.12 ± 0 |
| Plexin B1 | PLXNB1 | 0.02 ± 0 |
| Poly [ADP-ribose] polymerase | PARP4 | 0.02 ± 0 |
| Polyadenylate-binding protein | PABPC4 | 0.05 ± 0 |
| Potassium calcium-activated channel subfamily N member 4 | KCNN4 | 0.08 ± 0 |
| Procollagen C-endopeptidase enhancer | PCOLCE | 0.645 ± 0.092 |
| Proenkephalin-A | PENK | 0.535 ± 0.134 |
| Proprotein convertase subtilisin/kexin type 1 inhibitor | PCSK1N | 3.84 ± 0.481 |
| Proprotein convertase subtilisin/kexin type 2 | PCSK2 | 0.06 ± 0 |
| Prosaposin | PSAP | 0.555 ± 0.134 |
| Prostaglandin-H2 D-isomerase | PTGDS | 1.775 ± 0.007 |
| Protein AMBP | AMBP | 1.235 ± 0.148 |
| Protein C, inactivator of coagulation factors Va and VIIIa | PROC | 0.215 ± 0.049 |
| Protein S | PROS1 | 0.545 ± 0.021 |
| Protein tyrosine phosphatase receptor type N | PTPRN | 0.04 ± 0 |
| Protein Wnt | WNT8A | 0.09 ± 0 |
| Protein-L-isoaspartate O-methyltransferase | PCMT1 | 0.495 ± 0.12 |
| protein-tyrosine-phosphatase | PTPRD | 0.05 ± 0 |
| protein-tyrosine-phosphatase | PTPRG | 0.045 ± 0.035 |
| protein-tyrosine-phosphatase | PTPRS | 0.06 ± 0.014 |
| Prothrombin | F2 | 1.795 ± 0.106 |
| RAB3A interacting protein | RAB3IP | 0.08 ± 0 |
| receptor protein-tyrosine kinase | EPHA4 | 0.13 ± 0.028 |
| receptor protein-tyrosine kinase | FLT3 | 0.07 ± 0 |
| Reelin | RELN | 0.055 ± 0.007 |
| Renin receptor | ATP6AP2 | 1.625 ± 0.007 |
| Repulsive guidance molecule BMP co-receptor b | RGMB | 0.38 ± 0.071 |
| Reticulon 4 receptor | RTN4R | 0.12 ± 0.057 |
| Retinoic acid receptor responder protein 2 | RARRES2 | 0.79 ± 0 |
| Rho GTPase activating protein 44 | ARHGAP44 | 0.04 ± 0 |
| Ribonuclease pancreatic | RNASE1 | 1.1 ± 0 |
| Ribulose-phosphate 3-epimerase | RPE | 0.16 ± 0 |
| RNA uridylyltransferase | TUT4 | 0.02 ± 0 |
| Secreted phosphoprotein 1 | SPP1 | 1.78 ± 0.212 |
| Secretogranin-2 | SCG2 | 1.28 ± 0.071 |
| Secretogranin-3 | SCG3 | 1.685 ± 0.007 |
| Seizure related 6 homolog like | SEZ6L | 0.29 ± 0.028 |
| Seizure related 6 homolog like 2 | SEZ6L2 | 0.125 ± 0.064 |
| Selenoprotein P N-terminal domain-containing protein | N/A | 0.2 ± 0 |
| Semaphorin 3F | SEMA3F | 0.045 ± 0.007 |
| Semaphorin 7A | SEMA7A | 0.89 ± 0.071 |
| Serotransferrin | INHCA | 6.795 ± 0.757 |
| Serotransferrin | TF | 7.98 ± 0.863 |
| Serpin family A member 10 | SERPINA10 | 0.73 ± 0.085 |
| Serpin family A member 3 | SERPINA3 | 0.905 ± 0.191 |
| Serpin family A member 5 | SERPINA5 | 0.13 ± 0.071 |
| Serpin family A member 6 | SERPINA6 | 0.73 ± 0 |
| Serpin family D member 1 | SERPIND1 | 1.805 ± 0.134 |
| Serpin family F member 1 | SERPINF1 | 2.01 ± 0.184 |
| Serpin family F member 2 | SERPINF2 | 0.795 ± 0.092 |
| Serpin family G member 1 | SERPING1 | 1.13 ± 0.085 |
| Serpin family I member 1 | SERPINI1 | 0.785 ± 0.403 |
| Serum amyloid A protein | LOC102150143 | 2.645 ± 0.007 |
| Serum amyloid A protein | SAA1 | 4.17 ± 1.089 |
| SET and MYND domain containing 3 | SMYD3 | 0.23 ± 0 |
| Shisa family member 6 | SHISA6 | 0.07 ± 0 |
| SOGA family member 3 | SOGA3 | 0.04 ± 0 |
| Solute carrier family 34 member 1 | SLC34A1 | 0.11 ± 0 |
| Somatostatin | SST | 0.77 ± 0 |
| SPARC | SPARC | 1.08 ± 0 |
| SPARC like 1 | SPARCL1 | 1.525 ± 0.092 |
| SPARC related modular calcium binding 1 | SMOC1 | 0.07 ± 0 |
| Spermatosis associated 1 | SPATA1 | 0.08 ± 0 |
| Spondin-1 | SPON1 | 0.04 ± 0 |
| Stimulator of chondrosis 1 | SCRG1 | 1.6 ± 0 |
| Superoxide dismutase [Cu-Zn] | SOD1 | 2.525 ± 0.559 |
| Superoxide dismutase [Cu-Zn] | SOD3 | 0.36 ± 0.339 |
| Sushi domain-containing protein | N/A | 0.72 ± 0.042 |
| Sushi domain-containing protein | CFH | 1.71 ± 0.057 |
| Sushi domain-containing protein | LOC106783161 | 0.19 ± 0 |
| TAFA chemokine like family member 5 | TAFA5 | 0.18 ± 0 |
| TATA element modulatory factor 1 | TMF1 | 0.03 ± 0 |
| Tectonin beta-propeller repeat containing 2 | TECPR2 | 0.02 ± 0 |
| Tenascin R | TNR | 0.03 ± 0 |
| thioredoxin-disulfide reductase | HCFC2 | 0.06 ± 0 |
| Thy-1 cell surface antigen | THY1 | 1.27 ± 0 |
| Thymosin beta 4 X-linked | TMSB4X | 0.695 ± 0.403 |
| TIMP metallopeptidase inhibitor 2 | TIMP2 | 0.95 ± 0 |
| Transcobalamin 2 | TCN2 | 0.46 ± 0.255 |
| Transferrin | INHCA | 1.8 ± 0.099 |
| Transforming growth factor beta induced | TGFBI | 0.175 ± 0.092 |
| Transmembrane protein 132A | TMEM132A | 0.225 ± 0.064 |
| Transmembrane serine protease 13 | TMPRSS13 | 0.06 ± 0 |
| Transthyretin | TTR | 1.52 ± 0 |
| Triosephosphate isomerase | TPI1 | 0.13 ± 0 |
| Ubiquitin-60S ribosomal protein L40 | UBA52 | 3.365 ± 0.007 |
| ubiquitinyl hydrolase 1 | VCPIP1 | 0.03 ± 0 |
| Uncharacterized protein | LOC100059239 | 2.145 ± 0.007 |
| Unconventional myosin-VI | MYO6 | 0.04 ± 0.014 |
| URI1 prefoldin like chaperone | URI1 | 0.07 ± 0 |
| UTP20 small subunit processome component | UTP20 | 0.01 ± 0 |
| Vanin 1 | VNN1 | 0.435 ± 0.064 |
| Vasoactive intestinal peptide | VIP | 0.34 ± 0.184 |
| VGF nerve growth factor inducible | VGF | 1.585 ± 0.007 |
| Vitamin D-binding protein | GC | 2.865 ± 0.007 |
| Vitronectin | VTN | 0.735 ± 0.163 |
| Voltage-dependent R-type calcium channel subunit alpha | CACNA1E | 0.01 ± 0 |
| V-set and transmembrane domain containing 2A | VSTM2A | 0.77 ± 0 |
| V-set and transmembrane domain containing 2B | VSTM2B | 0.205 ± 0.106 |
| WAP domain-containing protein | LOC102148710 | 0.22 ± 0 |
| WAP, follistatin/kazal, immunoglobulin, kunitz and netrin domain containing 2 | WFIKKN2 | 0.19 ± 0.099 |
| Yip1 domain family member 3 | YIPF3 | 0.1 ± 0 |
| Z-DNA binding protein 1 | ZBP1 | 0.08 ± 0 |
| Zinc finger protein 543 | ZNF543 | 0.06 ± 0 |

## Table S2. Proteins Identified by ProteoMiner™ Small-Capacity Kit

Total proteins: 301

| **Protein Name** | **Gene Name** | **Mean emPAI ± SD** |
| --- | --- | --- |
| 6-phosphogluconate dehydrogenase, decarboxylating | PGD | 0.07 ± 0 |
| Abhydrolase domain containing 14A | ABHD14A | 0.28 ± 0 |
| Actin alpha 2, smooth muscle | ACTA2 | 0.285 ± 0.148 |
| Actin beta | ACTB | 0.385 ± 0.262 |
| ADAM metallopeptidase domain 22 | ADAM22 | 0.15 ± 0 |
| Adiponectin A | C1QB | 0.52 ± 0 |
| ADP-ribosylarginine hydrolase | ADPRH | 0.14 ± 0.071 |
| AE binding protein 1 | AEBP1 | 0.42 ± 0.057 |
| Afamin | AFM | 2.175 ± 0.346 |
| Albumin | ALB | 16.75 ± 0.834 |
| Alpha-1-antiproteinase 2 | N/A | 2.485 ± 0.573 |
| Alpha-1-antitrypsin | SPI2 | 3.45 ± 0.735 |
| Alpha-1-B glycoprotein | A1BG | 0.675 ± 0.078 |
| Alpha-2-HS-glycoprotein | AHSG | 1.855 ± 0.007 |
| Alpha-2-macroglobulin | N/A | 0.365 ± 0.148 |
| Alpha-2-macroglobulin | A2M | 0.35 ± 0.127 |
| Alpha-amylase | LOC100049851 | 0.3 ± 0 |
| Alpha-mannosidase | MAN2A2 | 0.125 ± 0.064 |
| Amyloid beta like protein 1 | APLP1 | 2.6 ± 0.297 |
| Amyloid beta precursor like protein 2 | LOC111767432 | 1.275 ± 0.148 |
| Amyloid-beta A4 protein | APP | 3.16 ± 0.156 |
| Anaphylatoxin-like domain-containing protein | N/A | 2.155 ± 0.007 |
| Angiotensinogen | AGT | 1.83 ± 0.438 |
| Antithrombin-III | SERPINC1 | 1.375 ± 0.474 |
| Apolipoprotein A-II | APOA2 | 3.94 ± 0.014 |
| Apolipoprotein C-II | APOC2 | 0.92 ± 0 |
| Apolipoprotein C-III | APOC3 | 1.085 ± 0.417 |
| Apolipoprotein D | APOD | 1.525 ± 0.53 |
| Apolipoprotein E | APOA1 | 21.39 ± 4.101 |
| Apolipoprotein E | APOA4 | 9.965 ± 3.896 |
| Apolipoprotein E | APOE | 15.455 ± 0.134 |
| ArfGAP with SH3 domain, ankyrin repeat and PH domain 2 | ASAP2 | 0.035 ± 0.007 |
| ATPase H+ transporting accessory protein 1 | ATP6AP1 | 0.55 ± 0 |
| Beta-1,3-N-acetylglucosaminyltransferase | LFNG | 0.28 ± 0.255 |
| Beta-1,4-galactosyltransferase | B4GALT1 | 0.12 ± 0.057 |
| Beta-1,4-glucuronyltransferase 1 | B4GAT1 | 1.925 ± 0.177 |
| Beta-2-glycoprotein 1 | APOH | 1.345 ± 0.007 |
| Beta-2-microglobulin | B2M | 0.68 ± 0 |
| BPI fold containing family A member 2 | BPIFA2 | 2.73 ± 0.354 |
| Brevican | BCAN | 0.85 ± 0.042 |
| Cache domain containing 1 | CACHD1 | 0.03 ± 0 |
| Cadherin 1 | CDH1 | 0.04 ± 0 |
| Cadherin 13 | CDH13 | 0.305 ± 0.049 |
| Cadherin 2 | CDH2 | 0.305 ± 0.007 |
| Cadherin EGF LAG seven-pass G-type receptor 2 | CELSR2 | 0.015 ± 0.007 |
| Calreticulin | CALR | 0.32 ± 0.071 |
| Calsyntenin 1 | CLSTN1 | 0.93 ± 0 |
| Carboxylic ester hydrolase | LOC100050764 | 0.13 ± 0 |
| Carboxylic ester hydrolase | LOC100050992 | 0.13 ± 0 |
| Carboxypeptidase E | CPE | 2.69 ± 0.17 |
| Cartilage acidic protein 1 | CRTAC1 | 1.21 ± 0.509 |
| Cathepsin B | CTSB | 0.245 ± 0.078 |
| Cathepsin D | CTSD | 1.05 ± 0.198 |
| Cathepsin F | CTSF | 0.47 ± 0.156 |
| C-C motif chemokine | LOC100630171 | 0.57 ± 0 |
| CD5 molecule like | CD5L | 0.33 ± 0.071 |
| Cell adhesion molecule 1 | CADM1 | 0.215 ± 0.163 |
| Cell adhesion molecule 3 | CADM3 | 0.15 ± 0.127 |
| Cell adhesion molecule L1 like | CHL1 | 0.835 ± 0.007 |
| Cellular communication network factor 3 | CCN3 | 0.725 ± 0.007 |
| Ceruloplasmin | CP | 1.745 ± 0.049 |
| Cholecystokinin | CCK | 0.355 ± 0.007 |
| Chordin like 1 | CHRDL1 | 0.15 ± 0.113 |
| Chromogranin A | CHGA | 4.855 ± 0.035 |
| Chromogranin B | CHGB | 0.765 ± 0.049 |
| Chromosome 13 C16orf89 homolog | C13H16orf89 | 0.735 ± 0.219 |
| Chromosome 4 open reading frame 48 | C4orf48 | 0.705 ± 0.417 |
| Clusterin | CLU | 5.785 ± 0.035 |
| Coagulation factor X | F10 | 0.15 ± 0.113 |
| Coagulation factor XII | F12 | 0.55 ± 0.113 |
| Coiled-coil domain containing 126 | CCDC126 | 0.27 ± 0 |
| Collagen type I alpha 1 chain | COL1A1 | 0.095 ± 0.064 |
| Collagen type I alpha 2 chain | COL1A2 | 0.03 ± 0 |
| Collagen type VI alpha 1 chain | COL6A1 | 0.17 ± 0.141 |
| Collagen type XII alpha 1 chain | COL12A1 | 0.105 ± 0.035 |
| Collagen type XVIII alpha 1 chain | COL18A1 | 0.195 ± 0.021 |
| Complement C1q C chain | C1QC | 0.655 ± 0.092 |
| Complement C1r | C1R | 0.555 ± 0.148 |
| Complement C1s | C1S | 1.075 ± 0.148 |
| Complement C2 | C2 | 0.545 ± 0.106 |
| Complement C2 | CFB | 0.935 ± 0.361 |
| Complement C3 | LOC100060505 | 4.915 ± 0.46 |
| Complement C5 | C5 | 0.755 ± 0.049 |
| Complement C7 | C7 | 0.555 ± 0.078 |
| Complement C8 alpha chain | C8A | 0.705 ± 0.064 |
| Complement C8 beta chain | C8B | 0.185 ± 0.092 |
| Complement C8 gamma chain | C8G | 0.41 ± 0.325 |
| Complement C9 | C9 | 1.12 ± 0.269 |
| Complement component 4 binding protein alpha | C4BPA | 0.66 ± 0.113 |
| Complement component C6 | C6 | 0.11 ± 0.057 |
| Complement factor D | CFD | 1.445 ± 0.431 |
| Complement factor I | CFI | 1.5 ± 0.283 |
| Contactin 2 | CNTN2 | 0.105 ± 0.106 |
| Contactin-1 | CNTN1 | 1.03 ± 0.226 |
| C-type lectin domain containing 11A | CLEC11A | 0.45 ± 0.113 |
| C-type lectin domain family 3 member B | CLEC3B | 1.28 ± 0.198 |
| Cyclin dependent kinase 1 | CDK1 | 0.15 ± 0 |
| Cystatin C | CST3 | 3.775 ± 0.785 |
| Cystatin E/M | CST6 | 1.775 ± 0.431 |
| Cysteine rich secretory protein LCCL domain containing 2 | CRISPLD2 | 0.07 ± 0 |
| Cytokine like 1 | CYTL1 | 1.575 ± 0.007 |
| Decorin | DCN | 1.04 ± 0.283 |
| Dickkopf WNT signaling pathway inhibitor 3 | DKK3 | 2.425 ± 0.163 |
| Dystroglycan 1 | DAG1 | 0.475 ± 0.078 |
| ECRG4 augurin | ECRG4 | 0.2 ± 0 |
| EGF containing fibulin extracellular matrix protein 1 | EFEMP1 | 1.535 ± 0.191 |
| EGF containing fibulin extracellular matrix protein 2 | EFEMP2 | 0.97 ± 0.085 |
| Extracellular matrix protein 1 | ECM1 | 1.645 ± 0.007 |
| Extracellular matrix protein 2 | ECM2 | 0.075 ± 0.035 |
| Family with sequence similarity 91 member A1 | FAM91A1 | 0.04 ± 0 |
| FAT atypical cadherin 2 | FAT2 | 0.03 ± 0.014 |
| Fetuin B | FETUB | 2.91 ± 0.481 |
| Fibrinogen alpha chain | FGA | 2.005 ± 0.064 |
| Fibrinogen alpha chain (Fragment) | FGA | 5.33 ± 0 |
| Fibrinogen beta chain | FGB | 4.685 ± 1.039 |
| Fibrinogen gamma chain | FGG | 4.405 ± 1.322 |
| Fibromodulin | FMOD | 0.09 ± 0 |
| Fibronectin | FN1 | 1.42 ± 0.014 |
| Fibulin 2 | FBLN2 | 0.155 ± 0.049 |
| Fibulin 5 | FBLN5 | 0.77 ± 0 |
| Fibulin-1 | FBLN1 | 1.21 ± 0.085 |
| Follistatin like 1 | FSTL1 | 0.43 ± 0.099 |
| Follistatin like 5 | FSTL5 | 0.08 ± 0 |
| FYVE, RhoGEF and PH domain containing 1 | FGD1 | 0.04 ± 0 |
| G protein-coupled receptor 37 | GPR37 | 0.06 ± 0 |
| Galectin-3-binding protein | LGALS3BP | 0.435 ± 0.233 |
| Gelsolin | GSN | 1.835 ± 0.148 |
| glutaminyl-peptide cyclotransferase | QPCT | 0.37 ± 0.085 |
| Glutathione peroxidase | GPX3 | 0.94 ± 0.198 |
| GM2 activator protein | GM2A | 1.93 ± 0.721 |
| Golgin subfamily A conserved domain-containing protein | GOLGA2 | 0.03 ± 0 |
| Haptoglobin | LOC100067869 | 5.61 ± 0.41 |
| Hemopexin | HPX | 1.58 ± 0.269 |
| Heparan sulfate proteoglycan 2 | HSPG2 | 0.01 ± 0 |
| Hepcidin antimicrobial peptide | Hamp | 0.455 ± 0.007 |
| Histidine rich glycoprotein | HRG | 3.275 ± 0.431 |
| HtrA serine peptidase 1 | HTRA1 | 0.14 ± 0.099 |
| Hyaluronan binding protein 2 | HABP2 | 0.345 ± 0.049 |
| Iduronate 2-sulfatase | IDS | 0.06 ± 0 |
| IF rod domain-containing protein | N/A | 0.165 ± 0.078 |
| IF rod domain-containing protein | KRT2 | 0.38 ± 0.057 |
| Ig-like domain-containing protein | N/A | 1.155 ± 0.106 |
| Immunoglobulin V-set domain-containing protein | N/A | 0.145 ± 0.078 |
| Insulin like growth factor binding protein 6 | IGFBP6 | 2.17 ± 0.014 |
| Insulin like growth factor binding protein 7 | IGFBP7 | 1.36 ± 1.004 |
| Insulin-like growth factor II | IGF2 | 0.225 ± 0.205 |
| Insulin-like growth factor-binding protein 2 | IGFBP2 | 3.355 ± 0.021 |
| Insulin-like growth factor-binding protein 4 | IGFBP4 | 0.48 ± 0.269 |
| Integral membrane protein 2 | ITM2B | 0.22 ± 0 |
| Integrin subunit beta like 1 | ITGBL1 | 0.42 ± 0.113 |
| Inter-alpha-trypsin inhibitor heavy chain 1 | ITIH1 | 0.93 ± 0.156 |
| Inter-alpha-trypsin inhibitor heavy chain 2 | ITIH2 | 1.195 ± 0.12 |
| Inter-alpha-trypsin inhibitor heavy chain 4 | ITIH4 | 1.85 ± 0.085 |
| Intercellular adhesion molecule 5 | ICAM5 | 0.19 ± 0.099 |
| Interferon gamma receptor 1 | IFNGR1 | 0.07 ± 0 |
| Interleukin 1 receptor accessory protein | IL1RAP | 0.11 ± 0.085 |
| Intraflagellar transport 74 | IFT74 | 0.06 ± 0 |
| Joining chain of multimeric IgA and IgM | JCHAIN | 0.84 ± 0 |
| Kallikrein B1 | KLKB1 | 0.175 ± 0.007 |
| Kallikrein related peptidase 6 | KLK6 | 0.82 ± 0.141 |
| Keratin 10A | KRT10A | 1.685 ± 0.007 |
| Keratin 15 | KRT15 | 0.42 ± 0.071 |
| Keratin 3 | KRT3 | 0.53 ± 0.127 |
| Keratin 6C | KRT85 | 0.47 ± 0.255 |
| Keratin 72 | KRT72 | 0.295 ± 0.12 |
| Keratin 77 | KRT77 | 0.2 ± 0 |
| Keratin, type II cytoskeletal 1 | KRT1 | 0.19 ± 0 |
| KIAA1549 like | KIAA1549L | 0.03 ± 0 |
| Kininogen 1 | KNG1 | 1.495 ± 0.46 |
| Leucine rich alpha-2-glycoprotein 1 | LRG1 | 0.16 ± 0.085 |
| Leucine rich repeat and Ig domain containing 2 | LINGO2 | 0.06 ± 0 |
| Limbic system associated membrane protein | LSAMP | 0.57 ± 0.325 |
| Lipocalin/cytosolic fatty-acid binding domain-containing protein | LOC100051562 | 0.19 ± 0 |
| Lipopolysaccharide-binding protein | LBP | 0.24 ± 0 |
| Lumican | LUM | 1.075 ± 0.417 |
| Ly6/neurotoxin 1 | LYNX1 | 0.335 ± 0.106 |
| Lymphocyte antigen 6 family member H | LY6H | 0.14 ± 0 |
| Major prion protein | PRNP | 0.715 ± 0.007 |
| Matrix Gla protein | MGP | 1.1 ± 0.424 |
| Matrix metallopeptidase 2 | MMP2 | 1.04 ± 0 |
| MBL associated serine protease 1 | MASP1 | 0.16 ± 0.085 |
| Metalloproteinase inhibitor 1 | TIMP1 | 0.27 ± 0.141 |
| Microfibril associated protein 4 | MFAP4 | 0.46 ± 0.255 |
| Monocyte differentiation antigen CD14 | CD14 | 0.6 ± 0 |
| Multiple EGF like domains 10 | MEGF10 | 0.045 ± 0.021 |
| Multiple inositol polyphosphate phosphatase 1 | MINPP1 | 0.805 ± 0.134 |
| Neogenin 1 | NEO1 | 0.06 ± 0.057 |
| Neural cell adhesion molecule 1 | NCAM1 | 0.84 ± 0.042 |
| Neural EGFL like 2 | NELL2 | 1.535 ± 0.064 |
| Neural proliferation, differentiation and control 1 | NPDC1 | 0.11 ± 0 |
| Neurexin 1 | NRXN1 | 0.1 ± 0.071 |
| Neurexin 2 | NRXN2 | 0.07 ± 0.042 |
| Neurexin 3 | NRXN3 | 0.16 ± 0.099 |
| Neuritin 1 | NRN1 | 0.27 ± 0 |
| Neuroblastoma suppressor of tumorigenicity 1 | NBL1 | 0.755 ± 0.007 |
| Neurocan | NCAN | 0.47 ± 0.057 |
| Neurofascin | NFASC | 0.08 ± 0.014 |
| Neuronal cell adhesion molecule | NRCAM | 0.94 ± 0.042 |
| Neuronal growth regulator 1 | NEGR1 | 0.47 ± 0.198 |
| Neuronal pentraxin 1 | NPTX1 | 2.3 ± 0.382 |
| Neuronal pentraxin 2 | NPTX2 | 0.375 ± 0.007 |
| Neuronal pentraxin receptor | NPTXR | 2.12 ± 0.325 |
| Nidogen 1 | NID1 | 0.185 ± 0.092 |
| Nidogen 2 | NID2 | 0.03 ± 0 |
| non-specific serine/threonine protein kinase | MAPKAPK5 | 0.07 ± 0 |
| NPC intracellular cholesterol transporter 2 | NPC2 | 2.22 ± 0.014 |
| NTR domain-containing protein | LOC100060539 | 3.08 ± 0.382 |
| Nucleobindin 1 | NUCB1 | 0.83 ± 0.085 |
| Olfactomedin 1 | OLFM1 | 0.19 ± 0.057 |
| Olfactomedin 3 | OLFML3 | 0.65 ± 0.156 |
| Olfactory receptor family 2 subfamily T member 68 | OR2T68 | 0.11 ± 0 |
| Oligodendrocyte myelin glycoprotein | OMG | 0.405 ± 0.078 |
| Osteoglycin | OGN | 1.985 ± 0.007 |
| Osteomodulin | OMD | 0.26 ± 0 |
| OTU deubiquitinase with linear linkage specificity | OTULIN | 0.1 ± 0 |
| Out at first protein homolog | POU2F3 | 0.65 ± 0.099 |
| Paraoxonase | PON1 | 1.22 ± 0.141 |
| Peptidylglycine alpha-amidating monooxygenase | PAM | 0.84 ± 0 |
| Peroxiredoxin 2 | PRDX2 | 0.53 ± 0.184 |
| Phospholipase D family member 3 | PLD3 | 0.365 ± 0.064 |
| Plasma retinol-binding protein | LOC111773844 | 0.57 ± 0.085 |
| Plasminogen | PLG | 3.145 ± 0.46 |
| Platelet derived growth factor subunit B | PDGFB | 0.14 ± 0 |
| Pleckstrin homology, MyTH4 and FERM domain containing H2 | PLEKHH2 | 0.02 ± 0 |
| Plexin B2 | PLXNB2 | 0.02 ± 0 |
| Prenylcysteine oxidase 1 | PCYOX1 | 0.105 ± 0.049 |
| Procollagen C-endopeptidase enhancer | PCOLCE | 1.24 ± 0.127 |
| Proenkephalin-A | PENK | 0.36 ± 0.113 |
| Proprotein convertase subtilisin/kexin type 1 inhibitor | PCSK1N | 2 ± 0.014 |
| Proprotein convertase subtilisin/kexin type 2 | PCSK2 | 0.09 ± 0.042 |
| Prosaposin | PSAP | 1.575 ± 0.233 |
| Prostaglandin-H2 D-isomerase | PTGDS | 0.825 ± 0.219 |
| Protein AMBP | AMBP | 1.485 ± 0.502 |
| Protein C, inactivator of coagulation factors Va and VIIIa | PROC | 0.4 ± 0.113 |
| Protein S | PROS1 | 0.435 ± 0.177 |
| Protein tyrosine phosphatase receptor type N | PTPRN | 0.04 ± 0 |
| Protein-L-isoaspartate O-methyltransferase | PCMT1 | 0.675 ± 0.134 |
| protein-tyrosine-phosphatase | PTPRS | 0.045 ± 0.007 |
| Prothrombin | F2 | 2.965 ± 0.276 |
| receptor protein-tyrosine kinase | EPHA4 | 0.055 ± 0.021 |
| Reelin | RELN | 0.08 ± 0.028 |
| Renin receptor | ATP6AP2 | 2.355 ± 0.219 |
| Repulsive guidance molecule BMP co-receptor b | RGMB | 0.24 ± 0.127 |
| Retinoic acid receptor responder protein 2 | RARRES2 | 0.795 ± 0.007 |
| Ribonuclease A family member 4 | RNASE4 | 0.365 ± 0.191 |
| RNA uridylyltransferase | TUT4 | 0.02 ± 0 |
| Secreted phosphoprotein 1 | SPP1 | 2.1 ± 0.226 |
| Secretogranin-2 | SCG2 | 1.395 ± 0.092 |
| Secretogranin-3 | SCG3 | 3.43 ± 0.467 |
| Seizure related 6 homolog | SEZ6 | 0.055 ± 0.021 |
| Seizure related 6 homolog like | SEZ6L | 0.38 ± 0.099 |
| Seizure related 6 homolog like 2 | SEZ6L2 | 0.125 ± 0.064 |
| Selenoprotein P N-terminal domain-containing protein | N/A | 0.205 ± 0.007 |
| Semaphorin 3F | SEMA3F | 0.05 ± 0 |
| Semaphorin 7A | SEMA7A | 0.295 ± 0.092 |
| Serotransferrin | INHCA | 4.47 ± 0.849 |
| Serotransferrin | TF | 5.345 ± 0.46 |
| Serpin family A member 10 | SERPINA10 | 0.8 ± 0.184 |
| Serpin family A member 3 | SERPINA3 | 1.28 ± 0.113 |
| Serpin family A member 6 | SERPINA6 | 0.54 ± 0.085 |
| Serpin family D member 1 | SERPIND1 | 1.545 ± 0.007 |
| Serpin family F member 1 | SERPINF1 | 2.025 ± 0.163 |
| Serpin family F member 2 | SERPINF2 | 0.8 ± 0.085 |
| Serpin family G member 1 | SERPING1 | 0.895 ± 0.078 |
| Serpin family I member 1 | SERPINI1 | 0.77 ± 0 |
| Serum amyloid A protein | LOC102150143 | 3.74 ± 0.014 |
| Serum amyloid A protein | SAA1 | 4.955 ± 0.021 |
| SLIT and NTRK like family member 1 | SLITRK1 | 0.185 ± 0.035 |
| SOGA family member 3 | SOGA3 | 0.04 ± 0 |
| Somatostatin | SST | 0.77 ± 0 |
| SPARC | SPARC | 1.585 ± 0.389 |
| SPARC like 1 | SPARCL1 | 1.655 ± 0.53 |
| Spermatosis associated 1 | SPATA1 | 0.12 ± 0.057 |
| Spondin-1 | SPON1 | 0.09 ± 0 |
| Stimulator of chondrosis 1 | SCRG1 | 2.1 ± 0.707 |
| Superoxide dismutase [Cu-Zn] | SOD3 | 0.6 ± 0 |
| Sushi domain-containing protein | N/A | 0.88 ± 0 |
| Sushi domain-containing protein | CFH | 2.49 ± 0.269 |
| Sushi domain-containing protein | LOC106783161 | 0.43 ± 0.339 |
| Sushi domain-containing protein | LOC111771285 | 0.62 ± 0 |
| SWI/SNF related, matrix associated, actin dependent regulator of chromatin subfamily c member 1 | SMARCC1 | 0.13 ± 0 |
| Thy-1 cell surface antigen | THY1 | 0.23 ± 0 |
| TIMP metallopeptidase inhibitor 2 | TIMP2 | 0.75 ± 0 |
| Titin | TTN | 0 ± 0 |
| Transcobalamin 2 | TCN2 | 0.79 ± 0 |
| Transferrin | INHCA | 2.435 ± 0.573 |
| Transforming growth factor beta induced | TGFBI | 0.335 ± 0.064 |
| Transmembrane serine protease 13 | TMPRSS13 | 0.06 ± 0 |
| Transthyretin | TTR | 2.18 ± 0.014 |
| Tripeptidyl-peptidase 1 | TPP1 | 0.095 ± 0.049 |
| Uncharacterized protein | N/A | 0.345 ± 0.049 |
| Uncharacterized protein | LOC100059239 | 2.415 ± 0.247 |
| Vasoactive intestinal peptide | VIP | 0.21 ± 0 |
| VGF nerve growth factor inducible | VGF | 1.14 ± 0.255 |
| Vitamin D-binding protein | GC | 3.925 ± 0.262 |
| Vitronectin | VTN | 0.995 ± 0.191 |
| Voltage-dependent R-type calcium channel subunit alpha | CACNA1E | 0.01 ± 0 |
| V-set and transmembrane domain containing 2A | VSTM2A | 0.15 ± 0 |
| WAP domain-containing protein | LOC102148710 | 0.36 ± 0.198 |
| WAP, follistatin/kazal, immunoglobulin, kunitz and netrin domain containing 2 | WFIKKN2 | 0.355 ± 0.332 |
| Z-DNA binding protein 1 | ZBP1 | 0.08 ± 0 |

## Table S3. Proteins Identified by PreOmics® ENRICH-iST Kit

Total proteins: 487

| **Protein Name** | **Gene Name** | **Mean emPAI ± SD** |
| --- | --- | --- |
| 3~(2~), 5~-bisphosphate nucleotidase 2 | BPNT2 | 0.21 ± 0 |
| 45 kDa calcium-binding protein | SDF4 | 0.08 ± 0 |
| 5~-3~ exoribonuclease | XRN2 | 0.04 ± 0 |
| 78 kDa glucose-regulated protein | HSPA5 | 0.37 ± 0 |
| ABI family member 3 binding protein | ABI3BP | 0.07 ± 0 |
| Actin beta | ACTB | 1.06 ± 0 |
| Activated leukocyte cell adhesion molecule | ALCAM | 0.13 ± 0 |
| ADAM like decysin 1 | ADAMDEC1 | 0.07 ± 0 |
| ADAM metallopeptidase domain 15 | ADAM15 | 0.04 ± 0 |
| ADAM metallopeptidase domain 22 | ADAM22 | 0.11 ± 0 |
| ADAM metallopeptidase with thrombospondin type 1 motif 3 | ADAMTS3 | 0.03 ± 0 |
| Adenylate cyclase type 5 | ADCY5 | 0.03 ± 0 |
| Adiponectin D | ADIPOQ | 0.25 ± 0 |
| ADP-ribosylarginine hydrolase | ADPRH | 0.19 ± 0 |
| AE binding protein 1 | AEBP1 | 0.26 ± 0 |
| Afamin | AFM | 1.12 ± 0 |
| AHNAK nucleoprotein | AHNAK | 0.015 ± 0.007 |
| A-kinase anchoring protein 9 | AKAP9 | 0.02 ± 0 |
| alanine transaminase | GPT2 | 0.07 ± 0 |
| Albumin | ALB | 7.14 ± 0 |
| Alpha-1,6-mannosyl-glycoprotein 2-beta-N-acetylglucosaminyltransferase | MGAT2 | 0.08 ± 0 |
| Alpha-1-antitrypsin | SPI2 | 1.63 ± 0 |
| Alpha-1-B glycoprotein | A1BG | 0.15 ± 0 |
| Alpha-2-HS-glycoprotein | AHSG | 1.37 ± 0 |
| Alpha-2-macroglobulin | N/A | 0.08 ± 0 |
| Alpha-2-macroglobulin | A2M | 0.12 ± 0 |
| Alpha-2-macroglobulin like 1 | A2ML1 | 0.05 ± 0 |
| Alpha-fetoprotein | AFP | 0.06 ± 0 |
| Alpha-mannosidase | MAN2A2 | 0.24 ± 0 |
| Alpha-mannosidase | MAN2B1 | 0.07 ± 0 |
| Alsin Rho guanine nucleotide exchange factor ALS2 | ALS2 | 0.02 ± 0 |
| Amyloid beta like protein 1 | APLP1 | 1.88 ± 0 |
| Amyloid beta precursor like protein 2 | LOC111767432 | 0.47 ± 0 |
| Amyloid-beta A4 protein | APP | 1.37 ± 0.071 |
| Anaphylatoxin-like domain-containing protein | N/A | 1.16 ± 0 |
| Angiotensinogen | AGT | 1.53 ± 0 |
| Annexin | ANXA1 | 0.11 ± 0 |
| Annexin | ANXA2 | 1.53 ± 0 |
| Antithrombin-III | SERPINC1 | 2.37 ± 0 |
| Apolipoprotein A-II | APOA2 | 1.61 ± 0 |
| Apolipoprotein B | APOB | 0.01 ± 0 |
| Apolipoprotein C-II | APOC2 | 1.66 ± 0 |
| Apolipoprotein C-III | APOC3 | 3.27 ± 0 |
| Apolipoprotein D | APOD | 1.705 ± 0.29 |
| Apolipoprotein E | APOA1 | 12.59 ± 0 |
| Apolipoprotein E | APOA4 | 5.035 ± 0.375 |
| Apolipoprotein E | APOE | 20.7 ± 1.655 |
| Arachidonate 12-lipoxygenase, 12R type | ALOX12B | 0.05 ± 0 |
| ArfGAP with SH3 domain, ankyrin repeat and PH domain 2 | ASAP2 | 0.04 ± 0 |
| Arginase | ARG1 | 0.83 ± 0 |
| ATPase H+ transporting accessory protein 1 | ATP6AP1 | 0.08 ± 0 |
| Beta-1,4-galactosyltransferase | B4GALT1 | 0.08 ± 0 |
| Beta-1,4-glucuronyltransferase 1 | B4GAT1 | 0.75 ± 0 |
| Beta-1,4-N-acetyl-galactosaminyltransferase 1 | B4GALNT1 | 0.06 ± 0 |
| Beta-2-glycoprotein 1 | APOH | 0.94 ± 0 |
| Beta-2-microglobulin | B2M | 0.3 ± 0 |
| Bleomycin hydrolase | BLMH | 0.25 ± 0 |
| Bone morphotic protein 3 | BMP3 | 0.07 ± 0 |
| BPI fold containing family A member 2 | BPIFA2 | 1.3 ± 0 |
| Brain abundant membrane attached signal protein 1 | BASP1 | 0.25 ± 0 |
| Brevican | BCAN | 0.485 ± 0.035 |
| C1q and TNF related 5 | C1QTNF5 | 0.16 ± 0 |
| Cache domain containing 1 | CACHD1 | 0.03 ± 0 |
| Cadherin 13 | CDH13 | 0.1 ± 0 |
| Cadherin 2 | CDH2 | 0.16 ± 0 |
| Cadherin 4 | CDH4 | 0.04 ± 0 |
| Calcium activated nucleotidase 1 | CANT1 | 0.48 ± 0 |
| Calcium voltage-gated channel auxiliary subunit alpha2delta 1 | CACNA2D1 | 0.06 ± 0 |
| calcium/calmodulin-dependent protein kinase | CAMK2A | 0.07 ± 0 |
| Calpain 1 | CAPN1 | 0.04 ± 0 |
| Calpain small subunit 1 | CAPNS1 | 0.16 ± 0 |
| Calreticulin | CALR | 0.08 ± 0 |
| Calsyntenin 1 | CLSTN1 | 0.53 ± 0 |
| Carbonic anhydrase | CA2 | 0.14 ± 0 |
| Carboxylic ester hydrolase | LOC100050764 | 0.13 ± 0 |
| Carboxylic ester hydrolase | LOC100050992 | 0.13 ± 0 |
| Carboxypeptidase B2 | CPB2 | 0.47 ± 0 |
| Carboxypeptidase E | CPE | 0.825 ± 0.092 |
| Carboxypeptidase M | CPM | 0.08 ± 0 |
| Carboxypeptidase N subunit 1 | CPN1 | 0.15 ± 0 |
| Carboxypeptidase N subunit 2 | CPN2 | 0.06 ± 0 |
| Carboxypeptidase Q | CPQ | 0.07 ± 0 |
| Cartilage acidic protein 1 | CRTAC1 | 0.48 ± 0 |
| Cartilage oligomeric matrix protein | COMP | 0.05 ± 0 |
| Catalase | CAT | 0.12 ± 0 |
| Cathepsin D | CTSD | 0.68 ± 0 |
| Cathepsin F | CTSF | 0.47 ± 0 |
| Cathepsin H | LOC111775633 | 0.09 ± 0 |
| Cathepsin S | CTSS | 0.22 ± 0 |
| Cathepsin V | CTSV | 0.765 ± 0.106 |
| Cell adhesion molecule 3 | CADM3 | 0.12 ± 0 |
| Cell adhesion molecule L1 like | CHL1 | 0.28 ± 0 |
| Cellular communication network factor 6 | CCN6 | 0.13 ± 0 |
| Ceramide kinase like | CERKL | 0.06 ± 0 |
| Ceruloplasmin | CP | 0.55 ± 0 |
| Chitinase 3 like 1 | CHI3L1 | 0.22 ± 0.071 |
| Cholecystokinin | CCK | 0.36 ± 0 |
| Chromogranin A | CHGA | 1.81 ± 0 |
| Chromogranin B | CHGB | 0.68 ± 0 |
| Chromosome 13 C16orf89 homolog | C13H16orf89 | 0.44 ± 0 |
| Clusterin | CLU | 4.46 ± 0 |
| Coagulation factor IX | F9 | 0.08 ± 0 |
| Coagulation factor X | F10 | 0.41 ± 0 |
| Coagulation factor XII | F12 | 0.18 ± 0 |
| Collagen type I alpha 1 chain | COL1A1 | 0.23 ± 0 |
| Collagen type I alpha 2 chain | COL1A2 | 0.23 ± 0 |
| Collagen type III alpha 1 chain | COL3A1 | 0.105 ± 0.021 |
| Collagen type VI alpha 1 chain | COL6A1 | 0.27 ± 0 |
| Collagen type VI alpha 2 chain | COL6A2 | 0.04 ± 0 |
| Collagen type VI alpha 3 chain | COL6A3 | 0.04 ± 0 |
| Collagen type XII alpha 1 chain | COL12A1 | 0.22 ± 0 |
| Collagen type XIV alpha 1 chain | COL14A1 | 0.02 ± 0 |
| Collagen type XVIII alpha 1 chain | COL18A1 | 0.06 ± 0 |
| Complement C1q C chain | C1QC | 0.1 ± 0 |
| Complement C1r | C1R | 0.45 ± 0 |
| Complement C1s | C1S | 0.47 ± 0 |
| Complement C2 | C2 | 0.66 ± 0.057 |
| Complement C2 | CFB | 1.1 ± 0 |
| Complement C3 | LOC100060505 | 3.455 ± 0.064 |
| Complement C5 | C5 | 0.605 ± 0.064 |
| Complement C7 | C7 | 0.365 ± 0.035 |
| Complement C8 alpha chain | C8A | 0.57 ± 0 |
| Complement C8 beta chain | C8B | 0.18 ± 0 |
| Complement C8 gamma chain | C8G | 0.65 ± 0 |
| Complement C9 | C9 | 1.385 ± 0.106 |
| Complement component 4 binding protein alpha | C4BPA | 0.1 ± 0 |
| Complement component C6 | C6 | 0.23 ± 0.057 |
| Complement factor D | CFD | 0.465 ± 0.092 |
| Complement factor I | CFI | 0.44 ± 0 |
| Complement factor properdin | CFP | 0.07 ± 0 |
| Contactin 2 | CNTN2 | 0.74 ± 0.042 |
| Contactin 4 | CNTN4 | 0.03 ± 0 |
| Contactin-1 | CNTN1 | 1.91 ± 0.071 |
| Corneodesmosin | CDSN | 0.07 ± 0 |
| creatine kinase | LOC100056283 | 0.555 ± 0.092 |
| C-type lectin domain containing 11A | CLEC11A | 0.37 ± 0 |
| C-type lectin domain containing 16A | CLEC16A | 0.04 ± 0 |
| C-type lectin domain family 3 member B | CLEC3B | 1.75 ± 0 |
| CutA divalent cation tolerance homolog | CUTA | 0.3 ± 0 |
| Cystatin A | CSTA | 0.37 ± 0 |
| Cystatin C | CST3 | 1.05 ± 0 |
| Cystatin E/M | CST6 | 0.97 ± 0 |
| Cytokine like 1 | CYTL1 | 0.27 ± 0 |
| DDB1 and CUL4 associated factor 1 | DCAF1 | 0.02 ± 0 |
| Decorin | DCN | 0.75 ± 0.127 |
| Desmocollin 1 | DSC1 | 0.13 ± 0 |
| Desmoglein 1 | DSG1 | 0.2 ± 0 |
| Desmoplakin | DSP | 0.58 ± 0 |
| Dihydrolipoamide dehydrogenase | DLD | 0.12 ± 0.057 |
| dipeptidyl-peptidase I | CTSC | 0.06 ± 0 |
| Discs large MAGUK scaffold protein 5 | DLG5 | 0.02 ± 0 |
| DLC1 Rho GTPase activating protein | DLC1 | 0.03 ± 0 |
| Dystroglycan 1 | DAG1 | 0.12 ± 0 |
| Ectonucleotide pyrophosphatase/phosphodiesterase 2 | ENPP2 | 0.61 ± 0 |
| Ectopic P-granules 5 autophagy tethering factor | EPG5 | 0.01 ± 0 |
| EF-hand domain-containing protein | LOC100061699 | 0.42 ± 0 |
| EGF containing fibulin extracellular matrix protein 1 | EFEMP1 | 0.29 ± 0 |
| EGF containing fibulin extracellular matrix protein 2 | EFEMP2 | 0.14 ± 0 |
| Elongation factor 1-alpha 1 | EEF1A1 | 0.08 ± 0 |
| Exostosin like glycosyltransferase 2 | EXTL2 | 0.35 ± 0 |
| Extracellular matrix protein 1 | ECM1 | 0.96 ± 0 |
| Extracellular matrix protein 2 | ECM2 | 0.05 ± 0 |
| Family with sequence similarity 174 member A | FAM174A | 0.2 ± 0 |
| Family with sequence similarity 91 member A1 | FAM91A1 | 0.04 ± 0 |
| FAT atypical cadherin 1 | FAT1 | 0.01 ± 0 |
| FAT atypical cadherin 2 | FAT2 | 0.265 ± 0.007 |
| Fatty acid binding protein 5 | FABP5 | 0.89 ± 0 |
| Fetuin B | FETUB | 1.375 ± 0.148 |
| Fibrinogen alpha chain | FGA | 1.38 ± 0 |
| Fibrinogen alpha chain (Fragment) | FGA | 5.33 ± 0 |
| Fibrinogen beta chain | FGB | 3.63 ± 0 |
| Fibrinogen gamma chain | FGG | 2.36 ± 0 |
| Fibrinogen like 1 | FGL1 | 0.17 ± 0.085 |
| Fibroblast growth factor receptor | FGFR1 | 0.08 ± 0 |
| Fibronectin | FN1 | 0.75 ± 0 |
| Fibrous sheath interacting protein 2 | FSIP2 | 0.01 ± 0 |
| Fibrous sheath interacting protein 2 like | FSIP2L | 0.02 ± 0 |
| Fibulin 2 | FBLN2 | 0.06 ± 0 |
| Fibulin 5 | FBLN5 | 0.29 ± 0 |
| Fibulin-1 | FBLN1 | 0.365 ± 0.049 |
| Filamin A | FLNA | 0.01 ± 0 |
| folate gamma-glutamyl hydrolase | GGH | 0.39 ± 0 |
| Follistatin like 1 | FSTL1 | 0.36 ± 0 |
| Follistatin like 4 | FSTL4 | 0.13 ± 0 |
| Follistatin like 5 | FSTL5 | 0.04 ± 0 |
| Fructose-bisphosphate aldolase | ALDOA | 0.97 ± 0 |
| G protein pathway suppressor 1 | GPS1 | 0.09 ± 0.042 |
| G protein-coupled receptor 37 | GPR37 | 0.12 ± 0 |
| Galectin-3-binding protein | LGALS3BP | 0.06 ± 0 |
| Gasdermin A | GSDMA | 0.16 ± 0 |
| GDNF family receptor alpha-2 | GFRA2 | 0.15 ± 0 |
| Gelsolin | GSN | 1.47 ± 0.071 |
| General vesicular transport factor p115 | USO1 | 0.04 ± 0 |
| Glucosylceramidase | GBA1 | 0.07 ± 0 |
| Glutathione peroxidase | GPX3 | 0.675 ± 0.177 |
| Glyceraldehyde-3-phosphate dehydrogenase | GAPDH | 0.69 ± 0 |
| Glycine receptor alpha 2 | GLRA2 | 0.08 ± 0 |
| GM2 activator protein | GM2A | 0.865 ± 0.233 |
| Golgi membrane protein 1 | GOLM1 | 1.43 ± 0 |
| GRIP and coiled-coil domain containing 2 | GCC2 | 0.02 ± 0 |
| Guanine deaminase | GDA | 0.08 ± 0 |
| H3.3 histone A | H3-3A | 0.08 ± 0 |
| Haptoglobin | LOC100067869 | 1.17 ± 0 |
| Heat shock protein 90 beta family member 1 | HSP90B1 | 0.04 ± 0 |
| Heat shock protein beta-1 | HSPB1 | 0.18 ± 0 |
| Heat shock protein family A (Hsp70) member 8 | HSPA8 | 0.23 ± 0 |
| Hedgehog protein | SHH | 0.08 ± 0 |
| Hematopoietic SH2 domain containing | HSH2D | 0.1 ± 0 |
| Hemopexin | HPX | 2.58 ± 0.184 |
| Heparan sulfate proteoglycan 2 | HSPG2 | 0.01 ± 0 |
| High mobility group AT-hook 1 | HMGA1 | 0.37 ± 0 |
| Histidine ammonia-lyase | HAL | 0.25 ± 0 |
| Histidine rich glycoprotein | HRG | 1.82 ± 0 |
| Histone H2B | H2BC7 | 0.7 ± 0 |
| Histone H4 | H4C13 | 0.38 ± 0 |
| HtrA serine peptidase 1 | HTRA1 | 0.14 ± 0 |
| Hyaluronan binding protein 2 | HABP2 | 0.21 ± 0 |
| IF rod domain-containing protein | N/A | 0.7 ± 0 |
| IF rod domain-containing protein | KRT10B | 2.27 ± 0 |
| IF rod domain-containing protein | KRT2 | 0.69 ± 0 |
| Ig-like domain-containing protein | N/A | 1.54 ± 0 |
| ILEI/PANDER domain-containing protein | N/A | 1.66 ± 0 |
| Immunoglobulin superfamily member 8 | IGSF8 | 0.26 ± 0 |
| Immunoglobulin V-set domain-containing protein | N/A | 0.1 ± 0 |
| Insulin like growth factor binding protein 4 | IGFBP4 | 0.15 ± 0 |
| Insulin like growth factor binding protein 7 | IGFBP7 | 0.65 ± 0 |
| Insulin like growth factor binding protein acid labile subunit | IGFALS | 0.12 ± 0 |
| Insulin-like growth factor-binding protein 2 | IGFBP2 | 1.87 ± 0 |
| Integrin subunit beta like 1 | ITGBL1 | 0.06 ± 0 |
| Inter-alpha-trypsin inhibitor heavy chain 1 | ITIH1 | 0.66 ± 0.042 |
| Inter-alpha-trypsin inhibitor heavy chain 2 | ITIH2 | 0.77 ± 0 |
| Inter-alpha-trypsin inhibitor heavy chain 4 | ITIH4 | 1.4 ± 0 |
| Inter-alpha-trypsin inhibitor heavy chain 5 | ITIH5 | 0.245 ± 0.035 |
| Intercellular adhesion molecule 5 | ICAM5 | 0.06 ± 0.028 |
| Interferon induced protein with tetratricopeptide repeats 2 | IFIT2 | 0.07 ± 0 |
| Interleukin 1 receptor accessory protein | IL1RAP | 0.05 ± 0 |
| Interleukin 12 receptor subunit beta 2 | IL12RB2 | 0.04 ± 0 |
| Intraflagellar transport 140 | IFT140 | 0.02 ± 0 |
| Intraflagellar transport 74 | IFT74 | 0.06 ± 0 |
| Joining chain of multimeric IgA and IgM | JCHAIN | 0.365 ± 0.191 |
| Junction plakoglobin | JUP | 0.515 ± 0.035 |
| Kallikrein B1 | KLKB1 | 0.06 ± 0 |
| Kallikrein related peptidase 6 | KLK6 | 0.55 ± 0 |
| Kazal-like domain-containing protein | SPINK2 | 0.46 ± 0 |
| Keratin 15 | KRT15 | 1.36 ± 0 |
| Keratin 24 | KRT24 | 0.32 ± 0 |
| Keratin 27 | KRT27 | 0.69 ± 0 |
| Keratin 3 | KRT3 | 1.55 ± 0.113 |
| Keratin 4 | KRT8 | 0.48 ± 0 |
| Keratin 42 | KRT42 | 1.12 ± 0 |
| Keratin 6C | KRT85 | 3.38 ± 0 |
| Keratin 72 | KRT72 | 0.62 ± 0.071 |
| Keratin 75 | KRT75 | 1.29 ± 0 |
| Keratin 77 | KRT77 | 0.62 ± 0 |
| Keratin 78 | KRT78 | 0.39 ± 0 |
| Keratin 80 | KRT80 | 0.6 ± 0 |
| Keratin, type II cytoskeletal 1 | KRT1 | 0.545 ± 0.064 |
| KIAA1549 like | KIAA1549L | 0.11 ± 0 |
| Kinesin-like protein | KIF5C | 0.055 ± 0.021 |
| Kininogen 1 | KNG1 | 0.96 ± 0 |
| L1 cell adhesion molecule | L1CAM | 0.03 ± 0 |
| Lactotransferrin | LTF | 0.075 ± 0.035 |
| Lamin A/C | LMNA | 0.11 ± 0 |
| Laminin subunit alpha 2 | LAMA2 | 0.02 ± 0 |
| Laminin subunit beta 1 | LAMB1 | 0.02 ± 0 |
| Laminin subunit gamma 1 | LAMC1 | 0.02 ± 0 |
| Latent transforming growth factor beta binding protein 1 | LTBP1 | 0.02 ± 0 |
| Latent transforming growth factor beta binding protein 4 | LTBP4 | 0.04 ± 0 |
| LDL receptor related protein 1 | LRP1 | 0.01 ± 0 |
| Lectin, mannose binding 2 | LMAN2 | 0.1 ± 0 |
| LEM domain nuclear envelope protein 2 | LEMD2 | 0.06 ± 0 |
| Lemur tyrosine kinase 3 | LMTK3 | 0.02 ± 0 |
| Leucine rich alpha-2-glycoprotein 1 | LRG1 | 1.09 ± 0.141 |
| Leucine rich repeat containing 4B | LRRC4B | 0.23 ± 0 |
| Leukocyte cell derived chemotaxin 2 | LECT2 | 0.25 ± 0 |
| LFNG O-fucosylpeptide 3-beta-N-acetylglucosaminyltransferase | LFNG | 0.3 ± 0 |
| Limbic system associated membrane protein | LSAMP | 0.8 ± 0 |
| Lipocalin/cytosolic fatty-acid binding domain-containing protein | LOC100068210 | 0.2 ± 0 |
| Lipopolysaccharide-binding protein | LBP | 0.24 ± 0 |
| L-lactate dehydrogenase | LDHA | 0.1 ± 0 |
| L-lactate dehydrogenase | LDHB | 0.14 ± 0.071 |
| Lumican | LUM | 0.34 ± 0 |
| Ly6/neurotoxin 1 | LYNX1 | 0.12 ± 0 |
| Lymphocyte cytosolic protein 1 | LCP1 | 0.17 ± 0 |
| Major prion protein | PRNP | 1.25 ± 0 |
| Malate dehydrogenase | MDH2 | 0.11 ± 0 |
| Mannose receptor C type 2 | MRC2 | 0.02 ± 0 |
| Matrix Gla protein | MGP | 0.8 ± 0 |
| Matrix metallopeptidase 2 | MMP2 | 0.89 ± 0.071 |
| Matrix remodeling-associated protein 8 | MXRA8 | 0.47 ± 0 |
| Meteorin like, glial cell differentiation regulator | METRNL | 0.37 ± 0 |
| Milk fat globule EGF and factor V/VIII domain containing | HAPLN3 | 0.15 ± 0 |
| mitogen-activated protein kinase kinase kinase | MAP3K15 | 0.05 ± 0 |
| Monocyte differentiation antigen CD14 | CD14 | 0.21 ± 0 |
| Multiple inositol polyphosphate phosphatase 1 | MINPP1 | 0.15 ± 0 |
| Myocilin | MYOC | 0.23 ± 0 |
| Myosin heavy chain 9 | MYH9 | 0.02 ± 0 |
| N-acetylglucosamine-1-phosphate transferase subunit gamma | GNPTG | 0.11 ± 0 |
| Neogenin 1 | NEO1 | 0.27 ± 0 |
| Netrin G1 | NTNG1 | 0.08 ± 0 |
| Neural cell adhesion molecule 1 | NCAM1 | 0.48 ± 0.028 |
| Neural cell adhesion molecule 2 | NCAM2 | 0.23 ± 0 |
| Neural EGFL like 2 | NELL2 | 0.08 ± 0 |
| Neurexin 1 | NRXN1 | 0.215 ± 0.021 |
| Neurexin 2 | NRXN2 | 0.17 ± 0 |
| Neurexin 3 | NRXN3 | 0.215 ± 0.021 |
| Neurexophilin | NXPH1 | 0.14 ± 0 |
| Neuroblastoma suppressor of tumorigenicity 1 | NBL1 | 0.46 ± 0 |
| Neurocan | NCAN | 0.14 ± 0 |
| Neuroendocrine protein 7B2 | SCG5 | 0.41 ± 0 |
| Neurofascin | NFASC | 0.39 ± 0 |
| Neuronal cell adhesion molecule | NRCAM | 0.705 ± 0.035 |
| Neuronal pentraxin 1 | NPTX1 | 1.4 ± 0 |
| Neuronal pentraxin 2 | NPTX2 | 0.27 ± 0 |
| Neuronal pentraxin receptor | NPTXR | 1.9 ± 0 |
| Neurotrimin | NTM | 0.63 ± 0 |
| Nidogen 1 | NID1 | 0.28 ± 0 |
| Nidogen 2 | NID2 | 0.14 ± 0 |
| NME/NM23 nucleoside diphosphate kinase 1 | NME2 | 0.46 ± 0 |
| non-specific serine/threonine protein kinase | ULK2 | 0.03 ± 0 |
| NPC intracellular cholesterol transporter 2 | NPC2 | 0.22 ± 0 |
| NTR domain-containing protein | LOC100060539 | 2.195 ± 0.163 |
| Nucleobindin 1 | NUCB1 | 0.53 ± 0 |
| Nucleophosmin 1 | NPM1 | 0.08 ± 0 |
| Olfactomedin 1 | OLFM1 | 0.15 ± 0 |
| Olfactomedin 3 | OLFML3 | 0.54 ± 0 |
| Oligodendrocyte myelin glycoprotein | OMG | 0.765 ± 0.092 |
| Opioid binding protein/cell adhesion molecule like | OPCML | 0.22 ± 0 |
| Osteoglycin | OGN | 1.4 ± 0 |
| Out at first protein homolog | POU2F3 | 0.31 ± 0 |
| Palmitoyl-protein thioesterase 1 | PPT1 | 0.24 ± 0 |
| Paraoxonase | PON1 | 0.21 ± 0 |
| PATJ crumbs cell polarity complex component | PATJ | 0.02 ± 0 |
| Pentraxin family member | CRP | 0.16 ± 0 |
| Peptidylglycine alpha-amidating monooxygenase | PAM | 0.36 ± 0 |
| Peroxiredoxin 2 | PRDX2 | 0.4 ± 0 |
| Peroxiredoxin-1 | N/A | 0.4 ± 0 |
| Peroxiredoxin-1 | PRDX1 | 2.23 ± 0 |
| Phosphatidylethanolamine binding protein 1 | PEBP1 | 1.04 ± 0 |
| Phosphatidylethanolamine binding protein 4 | PEBP4 | 0.54 ± 0 |
| Phosphoglycerate kinase | PGK1 | 0.09 ± 0 |
| Phospholipase D family member 3 | PLD3 | 0.275 ± 0.064 |
| Phospholipid transfer protein | PLTP | 0.7 ± 0 |
| Phospholipid-transporting ATPase | ATP11C | 0.03 ± 0 |
| phosphopyruvate hydratase | ENO1 | 0.22 ± 0 |
| Plakophilin 1 | PKP1 | 0.16 ± 0 |
| Plasma retinol-binding protein | LOC111773844 | 0.18 ± 0 |
| Plasminogen | PLG | 0.725 ± 0.049 |
| Platelet-derived growth factor (PDGF) family profile domain-containing protein | PDGFA | 0.06 ± 0 |
| Plectin | PLEC | 0.02 ± 0 |
| Plexin B2 | PLXNB2 | 0.02 ± 0 |
| POF1B actin binding protein | POF1B | 0.06 ± 0 |
| Polypeptide N-acetylgalactosaminyltransferase | GALNT10 | 0.12 ± 0 |
| Polypeptide N-acetylgalactosaminyltransferase | GALNT2 | 0.13 ± 0 |
| porphobilinogen synthase | ALAD | 0.09 ± 0 |
| Prenylcysteine oxidase 1 | PCYOX1 | 0.07 ± 0 |
| Pro-adrenomedullin | ADM | 0.16 ± 0 |
| Procollagen C-endopeptidase enhancer | PCOLCE | 0.71 ± 0 |
| procollagen-lysine 5-dioxygenase | PLOD1 | 0.09 ± 0 |
| procollagen-lysine 5-dioxygenase | PLOD3 | 0.05 ± 0 |
| Proline and arginine rich end leucine rich repeat protein | PRELP | 0.09 ± 0 |
| Prolylcarboxypeptidase | PRCP | 0.07 ± 0 |
| Proprotein convertase subtilisin/kexin type 1 inhibitor | PCSK1N | 2.96 ± 0 |
| Prosaposin | PSAP | 0.37 ± 0 |
| Prostaglandin-H2 D-isomerase | PTGDS | 1.35 ± 0 |
| Proteasome 20S subunit beta 1 | PSMB1 | 0.2 ± 0 |
| Proteasome subunit alpha type | PSMA1 | 0.28 ± 0 |
| Proteasome subunit alpha type | PSMA2 | 0.16 ± 0 |
| Proteasome subunit alpha type | PSMA3 | 0.39 ± 0.127 |
| Proteasome subunit alpha type | PSMA5 | 0.76 ± 0 |
| Proteasome subunit alpha type | PSMA7 | 0.31 ± 0 |
| Proteasome subunit beta | PSMB6 | 0.16 ± 0 |
| Protein AMBP | AMBP | 0.94 ± 0 |
| Protein C, inactivator of coagulation factors Va and VIIIa | PROC | 0.32 ± 0 |
| protein deglycase | PARK7 | 0.18 ± 0 |
| Protein disulfide-isomerase | PDIA3 | 0.07 ± 0 |
| Protein O-linked-mannose beta-1,2-N-acetylglucosaminyltransferase | POMGNT1 | 0.05 ± 0 |
| Protein S | PROS1 | 0.31 ± 0 |
| Protein S100 | S100A11 | 0.37 ± 0 |
| Protein S100 | S100A16 | 0.37 ± 0 |
| Protein S100 | S100A2 | 1.13 ± 0 |
| Protein tyrosine phosphatase receptor type N | PTPRN | 0.04 ± 0 |
| Protein tyrosine phosphatase receptor type N2 | PTPRN2 | 0.03 ± 0 |
| Protein-L-isoaspartate O-methyltransferase | PCMT1 | 0.675 ± 0.134 |
| protein-tyrosine-phosphatase | PTPRD | 0.12 ± 0.014 |
| protein-tyrosine-phosphatase | PTPRF | 0.03 ± 0.014 |
| protein-tyrosine-phosphatase | PTPRS | 0.07 ± 0 |
| Prothrombin | F2 | 0.655 ± 0.064 |
| Protocadherin 17 | PCDH17 | 0.03 ± 0 |
| Protocadherin 7 | PCDH7 | 0.03 ± 0 |
| Pyruvate kinase | PKM | 0.92 ± 0 |
| RAD50 double strand break repair protein | RAD50 | 0.04 ± 0.014 |
| receptor protein-tyrosine kinase | EPHA4 | 0.11 ± 0 |
| Reelin | RELN | 0.06 ± 0 |
| Renin receptor | ATP6AP2 | 0.97 ± 0 |
| Repulsive guidance molecule BMP co-receptor b | RGMB | 0.195 ± 0.064 |
| Reticulon 4 receptor | RTN4R | 0.16 ± 0 |
| Reticulon 4 receptor like 2 | RTN4RL2 | 0.18 ± 0 |
| Retinoic acid receptor responder protein 2 | RARRES2 | 0.8 ± 0 |
| Ribonuclease T2 | N/A | 0.31 ± 0.17 |
| Ring finger protein 214 | RNF214 | 0.04 ± 0 |
| RNA-binding protein FXR1 | FXR1 | 0.06 ± 0 |
| S100 calcium binding protein A14 | S100A14 | 0.89 ± 0 |
| Secreted frizzled related protein 4 | SFRP4 | 0.2 ± 0 |
| Secreted phosphoprotein 1 | SPP1 | 1.56 ± 0 |
| Secretogranin-2 | SCG2 | 0.805 ± 0.064 |
| Secretogranin-3 | SCG3 | 1.425 ± 0.12 |
| Seizure related 6 homolog like | SEZ6L | 0.09 ± 0.028 |
| Semaphorin 3F | SEMA3F | 0.05 ± 0 |
| Semaphorin 4D | SEMA4D | 0.04 ± 0 |
| Semaphorin 7A | SEMA7A | 1.02 ± 0 |
| Serotransferrin | TF | 2.515 ± 0.12 |
| Serpin domain-containing protein | LOC100057505 | 0.09 ± 0 |
| Serpin family A member 10 | SERPINA10 | 0.495 ± 0.078 |
| Serpin family A member 3 | SERPINA3 | 0.65 ± 0 |
| Serpin family A member 5 | SERPINA5 | 0.39 ± 0 |
| Serpin family A member 6 | SERPINA6 | 0.08 ± 0 |
| Serpin family B member 12 | SERPINB12 | 0.08 ± 0 |
| Serpin family B member 13 | SERPINB13 | 0.09 ± 0 |
| Serpin family D member 1 | SERPIND1 | 1.09 ± 0 |
| Serpin family F member 1 | SERPINF1 | 1.795 ± 0.163 |
| Serpin family F member 2 | SERPINF2 | 0.74 ± 0 |
| Serpin family G member 1 | SERPING1 | 1.07 ± 0 |
| Serum amyloid A protein | LOC100629928 | 1.87 ± 0 |
| Serum amyloid A protein | LOC102150143 | 1.85 ± 0 |
| Serum amyloid A protein | SAA1 | 2.29 ± 0 |
| Shisa family member 6 | SHISA6 | 0.07 ± 0 |
| Solute carrier family 12 member 7 | SLC12A7 | 0.03 ± 0 |
| Solute carrier family 34 member 1 | SLC34A1 | 0.11 ± 0 |
| Solute carrier family 39 member 10 | SLC39A10 | 0.04 ± 0 |
| Somatostatin | SST | 0.33 ± 0 |
| Sortilin related VPS10 domain containing receptor 1 | SORCS1 | 0.03 ± 0 |
| SPARC | SPARC | 0.11 ± 0 |
| SPARC like 1 | SPARCL1 | 0.88 ± 0 |
| SPARC related modular calcium binding 1 | SMOC1 | 0.15 ± 0 |
| Spectrin repeat containing nuclear envelope protein 1 | SYNE1 | 0 ± 0 |
| Spermatosis associated 1 | SPATA1 | 0.08 ± 0 |
| Spondin 2 | SPON2 | 0.1 ± 0 |
| Spondin-1 | SPON1 | 0.165 ± 0.035 |
| ST3 beta-galactoside alpha-2,3-sialyltransferase 6 | ST3GAL6 | 0.07 ± 0 |
| STAM binding protein like 1 | STAMBPL1 | 0.07 ± 0 |
| Stimulator of chondrosis 1 | SCRG1 | 0.9 ± 0 |
| Sulfhydryl oxidase | QSOX1 | 0.15 ± 0 |
| Sulfhydryl oxidase | QSOX2 | 0.1 ± 0 |
| Superoxide dismutase [Cu-Zn] | SOD3 | 0.51 ± 0.127 |
| Sushi domain-containing protein | N/A | 0.47 ± 0 |
| Sushi domain-containing protein | CFH | 0.97 ± 0 |
| Sushi domain-containing protein | LOC111771285 | 0.38 ± 0 |
| Sushi, nidogen and EGF like domains 1 | SNED1 | 0.02 ± 0 |
| TATA element modulatory factor 1 | TMF1 | 0.03 ± 0 |
| Tectonin beta-propeller repeat containing 2 | TECPR2 | 0.03 ± 0 |
| Thy-1 cell surface antigen | THY1 | 0.23 ± 0 |
| Thymosin beta 4 X-linked | TMSB4X | 0.98 ± 0 |
| Transcobalamin 2 | TCN2 | 0.46 ± 0.085 |
| Transferrin | INHCA | 0.47 ± 0 |
| Transforming growth factor beta induced | TGFBI | 0.5 ± 0 |
| Transglutaminase 1 | TGM1 | 0.11 ± 0.028 |
| Transglutaminase 3 | TGM3 | 0.05 ± 0 |
| Transketolase | TKT | 0.06 ± 0 |
| Transmembrane protein 132A | TMEM132A | 0.21 ± 0 |
| Transmembrane protein 132D | TMEM132D | 0.17 ± 0 |
| Transthyretin | TTR | 7.06 ± 0 |
| Triosephosphate isomerase | TPI1 | 0.28 ± 0 |
| Tudor domain containing 15 | TDRD15 | 0.02 ± 0 |
| Tyrosine 3-monooxygenase/tryptophan 5-monooxygenase activation protein zeta | YWHAZ | 0.3 ± 0 |
| Ubiquitin-60S ribosomal protein L40 | UBA52 | 2.43 ± 0 |
| Uncharacterized protein | N/A | 0.5 ± 0 |
| Uncharacterized protein | LOC100059239 | 1.39 ± 0 |
| Unconventional myosin-VI | MYO6 | 0.03 ± 0 |
| URI1 prefoldin like chaperone | URI1 | 0.07 ± 0 |
| Uteroglobin | SCGB1A1 | 0.44 ± 0 |
| Uveal autoantigen with coiled-coil domains and ankyrin repeats | UACA | 0.03 ± 0 |
| VGF nerve growth factor inducible | VGF | 0.86 ± 0 |
| Vinculin | VCL | 0.115 ± 0.021 |
| Vitamin D-binding protein | GC | 0.77 ± 0 |
| Vitronectin | VTN | 0.41 ± 0 |
| Voltage dependent anion channel 2 | VDAC2 | 0.12 ± 0 |
| Voltage-dependent R-type calcium channel subunit alpha | CACNA1E | 0.01 ± 0 |
| von Willebrand factor C domain containing 2 | VWC2 | 0.11 ± 0 |
| V-set and transmembrane domain containing 2A | VSTM2A | 0.78 ± 0 |
| V-set and transmembrane domain containing 2B | VSTM2B | 0.28 ± 0 |
| WAP domain-containing protein | LOC102148710 | 0.22 ± 0 |
| WAP, follistatin/kazal, immunoglobulin, kunitz and netrin domain containing 2 | WFIKKN2 | 0.06 ± 0 |
| Xyloside xylosyltransferase 1 | XXYLT1 | 0.27 ± 0 |
| Z-DNA binding protein 1 | ZBP1 | 0.08 ± 0 |
| Zinc finger and BTB domain containing 32 | ZBTB32 | 0.07 ± 0 |
| Zinc finger protein 543 | ZNF543 | 0.06 ± 0 |

## Table S4. Protein Overlaps Between Techniques

| **Overlap** | **Number of Proteins** | **Protein Name** | **Gene Name** |
| --- | --- | --- | --- |
| Native + ProteoMiner + PreOmics | 206 | ADAM metallopeptidase domain 22 | ADAM22 |
|  |  | AE binding protein 1 | AEBP1 |
|  |  | ATPase H+ transporting accessory protein 1 | ATP6AP1 |
|  |  | Afamin | AFM |
|  |  | Albumin | ALB |
|  |  | Alpha-1-B glycoprotein | A1BG |
|  |  | Alpha-1-antitrypsin | SPI2 |
|  |  | Alpha-2-HS-glycoprotein | AHSG |
|  |  | Alpha-2-macroglobulin | N/A |
|  |  | Alpha-mannosidase | MAN2A2 |
|  |  | Amyloid beta like protein 1 | APLP1 |
|  |  | Amyloid beta precursor like protein 2 | LOC111767432 |
|  |  | Amyloid-beta A4 protein | APP |
|  |  | Anaphylatoxin-like domain-containing protein | N/A |
|  |  | Angiotensinogen | AGT |
|  |  | Antithrombin-III | SERPINC1 |
|  |  | Apolipoprotein A-II | APOA2 |
|  |  | Apolipoprotein C-II | APOC2 |
|  |  | Apolipoprotein C-III | APOC3 |
|  |  | Apolipoprotein D | APOD |
|  |  | Apolipoprotein E | APOA1 |
|  |  | ArfGAP with SH3 domain, ankyrin repeat and PH domain 2 | ASAP2 |
|  |  | BPI fold containing family A member 2 | BPIFA2 |
|  |  | Beta-1,4-galactosyltransferase | B4GALT1 |
|  |  | Beta-1,4-glucuronyltransferase 1 | B4GAT1 |
|  |  | Beta-2-glycoprotein 1 | APOH |
|  |  | Beta-2-microglobulin | B2M |
|  |  | Brevican | BCAN |
|  |  | C-type lectin domain containing 11A | CLEC11A |
|  |  | C-type lectin domain family 3 member B | CLEC3B |
|  |  | Cache domain containing 1 | CACHD1 |
|  |  | Cadherin 13 | CDH13 |
|  |  | Cadherin 2 | CDH2 |
|  |  | Calreticulin | CALR |
|  |  | Calsyntenin 1 | CLSTN1 |
|  |  | Carboxylic ester hydrolase | LOC100050764 |
|  |  | Carboxypeptidase E | CPE |
|  |  | Cartilage acidic protein 1 | CRTAC1 |
|  |  | Cathepsin D | CTSD |
|  |  | Cathepsin F | CTSF |
|  |  | Cell adhesion molecule 3 | CADM3 |
|  |  | Cell adhesion molecule L1 like | CHL1 |
|  |  | Ceruloplasmin | CP |
|  |  | Cholecystokinin | CCK |
|  |  | Chromogranin A | CHGA |
|  |  | Chromogranin B | CHGB |
|  |  | Chromosome 13 C16orf89 homolog | C13H16orf89 |
|  |  | Clusterin | CLU |
|  |  | Coagulation factor X | F10 |
|  |  | Coagulation factor XII | F12 |
|  |  | Collagen type I alpha 1 chain | COL1A1 |
|  |  | Collagen type I alpha 2 chain | COL1A2 |
|  |  | Collagen type VI alpha 1 chain | COL6A1 |
|  |  | Collagen type XII alpha 1 chain | COL12A1 |
|  |  | Collagen type XVIII alpha 1 chain | COL18A1 |
|  |  | Complement C1q C chain | C1QC |
|  |  | Complement C1r | C1R |
|  |  | Complement C1s | C1S |
|  |  | Complement C2 | C2 |
|  |  | Complement C3 | LOC100060505 |
|  |  | Complement C5 | C5 |
|  |  | Complement C7 | C7 |
|  |  | Complement C8 alpha chain | C8A |
|  |  | Complement C8 beta chain | C8B |
|  |  | Complement C8 gamma chain | C8G |
|  |  | Complement C9 | C9 |
|  |  | Complement component 4 binding protein alpha | C4BPA |
|  |  | Complement component C6 | C6 |
|  |  | Complement factor D | CFD |
|  |  | Complement factor I | CFI |
|  |  | Contactin 2 | CNTN2 |
|  |  | Contactin-1 | CNTN1 |
|  |  | Cystatin C | CST3 |
|  |  | Cystatin E/M | CST6 |
|  |  | Cytokine like 1 | CYTL1 |
|  |  | Decorin | DCN |
|  |  | Dystroglycan 1 | DAG1 |
|  |  | EGF containing fibulin extracellular matrix protein 1 | EFEMP1 |
|  |  | EGF containing fibulin extracellular matrix protein 2 | EFEMP2 |
|  |  | Extracellular matrix protein 1 | ECM1 |
|  |  | FAT atypical cadherin 2 | FAT2 |
|  |  | Fetuin B | FETUB |
|  |  | Fibrinogen alpha chain | FGA |
|  |  | Fibrinogen alpha chain (Fragment) | FGA |
|  |  | Fibrinogen gamma chain | FGG |
|  |  | Fibulin 5 | FBLN5 |
|  |  | Fibulin-1 | FBLN1 |
|  |  | Follistatin like 1 | FSTL1 |
|  |  | G protein-coupled receptor 37 | GPR37 |
|  |  | GM2 activator protein | GM2A |
|  |  | Galectin-3-binding protein | LGALS3BP |
|  |  | Gelsolin | GSN |
|  |  | Glutathione peroxidase | GPX3 |
|  |  | Haptoglobin | LOC100067869 |
|  |  | Hemopexin | HPX |
|  |  | Heparan sulfate proteoglycan 2 | HSPG2 |
|  |  | Histidine rich glycoprotein | HRG |
|  |  | HtrA serine peptidase 1 | HTRA1 |
|  |  | Hyaluronan binding protein 2 | HABP2 |
|  |  | IF rod domain-containing protein | N/A |
|  |  | Ig-like domain-containing protein | N/A |
|  |  | Immunoglobulin V-set domain-containing protein | N/A |
|  |  | Insulin like growth factor binding protein 7 | IGFBP7 |
|  |  | Insulin-like growth factor-binding protein 2 | IGFBP2 |
|  |  | Integrin subunit beta like 1 | ITGBL1 |
|  |  | Inter-alpha-trypsin inhibitor heavy chain 1 | ITIH1 |
|  |  | Inter-alpha-trypsin inhibitor heavy chain 4 | ITIH4 |
|  |  | Intercellular adhesion molecule 5 | ICAM5 |
|  |  | Interleukin 1 receptor accessory protein | IL1RAP |
|  |  | Intraflagellar transport 74 | IFT74 |
|  |  | Joining chain of multimeric IgA and IgM | JCHAIN |
|  |  | KIAA1549 like | KIAA1549L |
|  |  | Kallikrein B1 | KLKB1 |
|  |  | Kallikrein related peptidase 6 | KLK6 |
|  |  | Keratin 15 | KRT15 |
|  |  | Keratin 6C | KRT85 |
|  |  | Keratin 72 | KRT72 |
|  |  | Keratin, type II cytoskeletal 1 | KRT1 |
|  |  | Kininogen 1 | KNG1 |
|  |  | Leucine rich alpha-2-glycoprotein 1 | LRG1 |
|  |  | Limbic system associated membrane protein | LSAMP |
|  |  | Lipocalin/cytosolic fatty-acid binding domain-containing protein | LOC100051562 |
|  |  | Lipopolysaccharide-binding protein | LBP |
|  |  | Lumican | LUM |
|  |  | Ly6/neurotoxin 1 | LYNX1 |
|  |  | Major prion protein | PRNP |
|  |  | Matrix Gla protein | MGP |
|  |  | Matrix metallopeptidase 2 | MMP2 |
|  |  | Monocyte differentiation antigen CD14 | CD14 |
|  |  | Multiple inositol polyphosphate phosphatase 1 | MINPP1 |
|  |  | NPC intracellular cholesterol transporter 2 | NPC2 |
|  |  | NTR domain-containing protein | LOC100060539 |
|  |  | Neogenin 1 | NEO1 |
|  |  | Neural EGFL like 2 | NELL2 |
|  |  | Neural cell adhesion molecule 1 | NCAM1 |
|  |  | Neurexin 1 | NRXN1 |
|  |  | Neurexin 2 | NRXN2 |
|  |  | Neurexin 3 | NRXN3 |
|  |  | Neuroblastoma suppressor of tumorigenicity 1 | NBL1 |
|  |  | Neurocan | NCAN |
|  |  | Neurofascin | NFASC |
|  |  | Neuronal cell adhesion molecule | NRCAM |
|  |  | Neuronal pentraxin 1 | NPTX1 |
|  |  | Neuronal pentraxin 2 | NPTX2 |
|  |  | Neuronal pentraxin receptor | NPTXR |
|  |  | Nidogen 1 | NID1 |
|  |  | Nucleobindin 1 | NUCB1 |
|  |  | Paraoxonase | PON1 |
|  |  | Peptidylglycine alpha-amidating monooxygenase | PAM |
|  |  | Phospholipase D family member 3 | PLD3 |
|  |  | Plasma retinol-binding protein | LOC111773844 |
|  |  | Plasminogen | PLG |
|  |  | Procollagen C-endopeptidase enhancer | PCOLCE |
|  |  | Proprotein convertase subtilisin/kexin type 1 inhibitor | PCSK1N |
|  |  | Prosaposin | PSAP |
|  |  | Prostaglandin-H2 D-isomerase | PTGDS |
|  |  | Protein AMBP | AMBP |
|  |  | Protein C, inactivator of coagulation factors Va and VIIIa | PROC |
|  |  | Protein S | PROS1 |
|  |  | Protein tyrosine phosphatase receptor type N | PTPRN |
|  |  | Prothrombin | F2 |
|  |  | Reelin | RELN |
|  |  | Renin receptor | ATP6AP2 |
|  |  | Repulsive guidance molecule BMP co-receptor b | RGMB |
|  |  | Retinoic acid receptor responder protein 2 | RARRES2 |
|  |  | SPARC | SPARC |
|  |  | SPARC like 1 | SPARCL1 |
|  |  | Secreted phosphoprotein 1 | SPP1 |
|  |  | Secretogranin-2 | SCG2 |
|  |  | Secretogranin-3 | SCG3 |
|  |  | Seizure related 6 homolog like | SEZ6L |
|  |  | Semaphorin 3F | SEMA3F |
|  |  | Semaphorin 7A | SEMA7A |
|  |  | Serotransferrin | INHCA |
|  |  | Serpin family A member 10 | SERPINA10 |
|  |  | Serpin family A member 3 | SERPINA3 |
|  |  | Serpin family A member 6 | SERPINA6 |
|  |  | Serpin family D member 1 | SERPIND1 |
|  |  | Serpin family F member 1 | SERPINF1 |
|  |  | Serpin family F member 2 | SERPINF2 |
|  |  | Serpin family G member 1 | SERPING1 |
|  |  | Serum amyloid A protein | LOC102150143 |
|  |  | Somatostatin | SST |
|  |  | Spermatosis associated 1 | SPATA1 |
|  |  | Spondin-1 | SPON1 |
|  |  | Stimulator of chondrosis 1 | SCRG1 |
|  |  | Superoxide dismutase [Cu-Zn] | SOD1 |
|  |  | Sushi domain-containing protein | N/A |
|  |  | Thy-1 cell surface antigen | THY1 |
|  |  | Transcobalamin 2 | TCN2 |
|  |  | Transferrin | INHCA |
|  |  | Transforming growth factor beta induced | TGFBI |
|  |  | Transthyretin | TTR |
|  |  | Uncharacterized protein | LOC100059239 |
|  |  | V-set and transmembrane domain containing 2A | VSTM2A |
|  |  | VGF nerve growth factor inducible | VGF |
|  |  | Vitamin D-binding protein | GC |
|  |  | Vitronectin | VTN |
|  |  | Voltage-dependent R-type calcium channel subunit alpha | CACNA1E |
|  |  | WAP domain-containing protein | LOC102148710 |
|  |  | WAP, follistatin/kazal, immunoglobulin, kunitz and netrin domain containing 2 | WFIKKN2 |
|  |  | Z-DNA binding protein 1 | ZBP1 |
|  |  | non-specific serine/threonine protein kinase | MAPKAPK5 |
|  |  | protein-tyrosine-phosphatase | PTPRD |
|  |  | receptor protein-tyrosine kinase | EPHA4 |
|  |  |  | ODC1 |
| Native + ProteoMiner | 33 | Adiponectin A | C1QB |
|  |  | Alpha-1-antiproteinase 2 | N/A |
|  |  | Alpha-amylase | LOC100049851 |
|  |  | Beta-1,3-N-acetylglucosaminyltransferase | LFNG |
|  |  | C-C motif chemokine | LOC100630171 |
|  |  | CD5 molecule like | CD5L |
|  |  | Cadherin EGF LAG seven-pass G-type receptor 2 | CELSR2 |
|  |  | Cathepsin B | CTSB |
|  |  | Cell adhesion molecule 1 | CADM1 |
|  |  | Cellular communication network factor 3 | CCN3 |
|  |  | Chromosome 4 open reading frame 48 | C4orf48 |
|  |  | Cysteine rich secretory protein LCCL domain containing 2 | CRISPLD2 |
|  |  | ECRG4 augurin | ECRG4 |
|  |  | Insulin like growth factor binding protein 6 | IGFBP6 |
|  |  | Insulin-like growth factor II | IGF2 |
|  |  | Integral membrane protein 2 | ITM2B |
|  |  | Lymphocyte antigen 6 family member H | LY6H |
|  |  | MBL associated serine protease 1 | MASP1 |
|  |  | Metalloproteinase inhibitor 1 | TIMP1 |
|  |  | Microfibril associated protein 4 | MFAP4 |
|  |  | Neural proliferation, differentiation and control 1 | NPDC1 |
|  |  | Neuritin 1 | NRN1 |
|  |  | Neuronal growth regulator 1 | NEGR1 |
|  |  | Proenkephalin-A | PENK |
|  |  | Proprotein convertase subtilisin/kexin type 2 | PCSK2 |
|  |  | RNA uridylyltransferase | TUT4 |
|  |  | Seizure related 6 homolog like 2 | SEZ6L2 |
|  |  | Selenoprotein P N-terminal domain-containing protein | N/A |
|  |  | Serpin family I member 1 | SERPINI1 |
|  |  | TIMP metallopeptidase inhibitor 2 | TIMP2 |
|  |  | Transmembrane serine protease 13 | TMPRSS13 |
|  |  | Vasoactive intestinal peptide | VIP |
|  |  | glutaminyl-peptide cyclotransferase | QPCT |
| Native + PreOmics | 57 | Activated leukocyte cell adhesion molecule | ALCAM |
|  |  | Adiponectin D | ADIPOQ |
|  |  | Calcium activated nucleotidase 1 | CANT1 |
|  |  | Calcium voltage-gated channel auxiliary subunit alpha2delta 1 | CACNA2D1 |
|  |  | Carboxypeptidase B2 | CPB2 |
|  |  | Carboxypeptidase M | CPM |
|  |  | Cathepsin V | CTSV |
|  |  | Ceramide kinase like | CERKL |
|  |  | Complement factor properdin | CFP |
|  |  | CutA divalent cation tolerance homolog | CUTA |
|  |  | DDB1 and CUL4 associated factor 1 | DCAF1 |
|  |  | Ectonucleotide pyrophosphatase/phosphodiesterase 2 | ENPP2 |
|  |  | Exostosin like glycosyltransferase 2 | EXTL2 |
|  |  | Fructose-bisphosphate aldolase | ALDOB |
|  |  | G protein pathway suppressor 1 | GPS1 |
|  |  | GDNF family receptor alpha-2 | GFRA2 |
|  |  | Golgi membrane protein 1 | GOLM1 |
|  |  | Guanine deaminase | GDA |
|  |  | Hedgehog protein | SHH |
|  |  | Hematopoietic SH2 domain containing | HSH2D |
|  |  | ILEI/PANDER domain-containing protein | N/A |
|  |  | Immunoglobulin superfamily member 8 | IGSF8 |
|  |  | Insulin like growth factor binding protein 4 | IGFBP4 |
|  |  | Inter-alpha-trypsin inhibitor heavy chain 5 | ITIH5 |
|  |  | Interleukin 12 receptor subunit beta 2 | IL12RB2 |
|  |  | Keratin 80 | KRT80 |
|  |  | L-lactate dehydrogenase | LDHA |
|  |  | Lactotransferrin | LTF |
|  |  | Latent transforming growth factor beta binding protein 4 | LTBP4 |
|  |  | Lectin, mannose binding 2 | LMAN2 |
|  |  | Myocilin | MYOC |
|  |  | Neural cell adhesion molecule 2 | NCAM2 |
|  |  | Neurexophilin | NXPH1 |
|  |  | Neuroendocrine protein 7B2 | SCG5 |
|  |  | Neurotrimin | NTM |
|  |  | Phosphatidylethanolamine binding protein 1 | PEBP1 |
|  |  | Phosphatidylethanolamine binding protein 4 | PEBP4 |
|  |  | Phospholipid transfer protein | PLTP |
|  |  | Phospholipid-transporting ATPase | ATP11C |
|  |  | Reticulon 4 receptor | RTN4R |
|  |  | SPARC related modular calcium binding 1 | SMOC1 |
|  |  | Serpin family A member 5 | SERPINA5 |
|  |  | Shisa family member 6 | SHISA6 |
|  |  | Solute carrier family 34 member 1 | SLC34A1 |
|  |  | TATA element modulatory factor 1 | TMF1 |
|  |  | Tectonin beta-propeller repeat containing 2 | TECPR2 |
|  |  | Thymosin beta 4 X-linked | TMSB4X |
|  |  | Transmembrane protein 132A | TMEM132A |
|  |  | Triosephosphate isomerase | TPI1 |
|  |  | URI1 prefoldin like chaperone | URI1 |
|  |  | Ubiquitin-60S ribosomal protein L40 | UBA52 |
|  |  | Unconventional myosin-VI | MYO6 |
|  |  | V-set and transmembrane domain containing 2B | VSTM2B |
|  |  | Zinc finger protein 543 | ZNF543 |
|  |  | calcium/calmodulin-dependent protein kinase | CAMK2A |
|  |  | dipeptidyl-peptidase I | CTSC |
|  |  | folate gamma-glutamyl hydrolase | GGH |
| ProteoMiner + PreOmics | 16 | ADP-ribosylarginine hydrolase | ADPRH |
|  |  | Actin beta | ACTB |
|  |  | Extracellular matrix protein 2 | ECM2 |
|  |  | Family with sequence similarity 91 member A1 | FAM91A1 |
|  |  | Fibrinogen beta chain | FGB |
|  |  | Fibronectin | FN1 |
|  |  | Fibulin 2 | FBLN2 |
|  |  | Follistatin like 5 | FSTL5 |
|  |  | Inter-alpha-trypsin inhibitor heavy chain 2 | ITIH2 |
|  |  | Keratin 3 | KRT3 |
|  |  | Keratin 77 | KRT77 |
|  |  | Nidogen 2 | NID2 |
|  |  | Olfactomedin like 3 | OLFML3 |
|  |  | Peroxiredoxin 2 | PRDX2 |
|  |  | Plexin B2 | PLXNB2 |
|  |  | Prenylcysteine oxidase 1 | PCYOX1 |
| Native only | 61 | 14-3-3 protein theta | YWHAQ |
|  |  | ATP binding cassette subfamily C member 13 | ABCC13 |
|  |  | ATP-dependent RNA helicase | DDX55 |
|  |  | Acetylcholinesterase (Cartwright blood group) | ACHE |
|  |  | Adhesion G protein-coupled receptor L1 | ADGRL1 |
|  |  | Adhesion G protein-coupled receptor V1 | ADGRV1 |
|  |  | Agrin | AGRN |
|  |  | Alpha-2-glycoprotein 1, zinc-binding | AZGP1 |
|  |  | Alpha-L-fucosidase | FUCA1 |
|  |  | Amine oxidase | AOC3 |
|  |  | Amyloid beta like protein 2 | APLP2 |
|  |  | Angiotensin-converting enzyme | ACE |
|  |  | Aspartate aminotransferase | GOT1 |
|  |  | Attractin | ATRN |
|  |  | Biotinidase | BTD |
|  |  | CD44 antigen | CD44 |
|  |  | Calsyntenin 3 | CLSTN3 |
|  |  | Centromere protein H | CENPH |
|  |  | Centrosomal protein of 162 kDa | CEP162 |
|  |  | Cochlin | COCH |
|  |  | Coiled-coil domain containing 110 | CCDC110 |
|  |  | Coiled-coil domain containing 138 | CCDC138 |
|  |  | Cryptochrome circadian regulator 1 | CRY1 |
|  |  | DNA topoisomerase I | TOP1MT |
|  |  | Delta/notch like EGF repeat containing | DNER |
|  |  | FYVE, RhoGEF and PH domain containing 3 | FGD3 |
|  |  | G protein-coupled receptor 37 like 1 | GPR37L1 |
|  |  | Gastrin releasing peptide | GRP |
|  |  | Globin family profile domain-containing protein | LOC100068926 |
|  |  | Inducible T cell costimulator ligand | ICOSLG |
|  |  | Kazal type serine peptidase inhibitor domain 1 | KAZALD1 |
|  |  | Leucine rich repeat and Ig domain containing 1 | LINGO1 |
|  |  | Lysocardiolipin acyltransferase 1 | LCLAT1 |
|  |  | Maltase-glucoamylase | MGAM |
|  |  | Mast/stem cell growth factor receptor Kit | KIT |
|  |  | Matrix metallopeptidase 17 | MMP17 |
|  |  | Membrane cofactor protein | LOC100057176 |
|  |  | Mucin 4, cell surface associated | MUC4 |
|  |  | N-acetyllactosaminide alpha-1,3-galactosyltransferase | LOC100067589 |
|  |  | Neuronal vesicle trafficking associated 1 | NSG1 |
|  |  | Neuropeptide Y | NPY |
|  |  | Peptidase inhibitor 16 | PI16 |
|  |  | Phosphoinositide-3-kinase interacting protein 1 | PIK3IP1 |
|  |  | Pleckstrin homology domain containing N1 | PLEKHN1 |
|  |  | Plexin B1 | PLXNB1 |
|  |  | Poly [ADP-ribose] polymerase | PARP4 |
|  |  | Polyadenylate-binding protein | PABPC4 |
|  |  | Potassium calcium-activated channel subfamily N member 4 | KCNN4 |
|  |  | Protein Wnt | WNT8A |
|  |  | RAB3A interacting protein | RAB3IP |
|  |  | Rho GTPase activating protein 44 | ARHGAP44 |
|  |  | Ribonuclease pancreatic | RNASE1 |
|  |  | Ribulose-phosphate 3-epimerase | RPE |
|  |  | SET and MYND domain containing 3 | SMYD3 |
|  |  | TAFA chemokine like family member 5 | TAFA5 |
|  |  | Tenascin R | TNR |
|  |  | UTP20 small subunit processome component | UTP20 |
|  |  | Vanin 1 | VNN1 |
|  |  | Yip1 domain family member 3 | YIPF3 |
|  |  | thioredoxin-disulfide reductase | HCFC2 |
|  |  | ubiquitinyl hydrolase 1 | VCPIP1 |
| ProteoMiner only | 26 | 6-phosphogluconate dehydrogenase, decarboxylating | PGD |
|  |  | Abhydrolase domain containing 14A | ABHD14A |
|  |  | Actin alpha 2, smooth muscle | ACTA2 |
|  |  | Cadherin 1 | CDH1 |
|  |  | Chordin like 1 | CHRDL1 |
|  |  | Coiled-coil domain containing 126 | CCDC126 |
|  |  | Cyclin dependent kinase 1 | CDK1 |
|  |  | Dickkopf WNT signaling pathway inhibitor 3 | DKK3 |
|  |  | FYVE, RhoGEF and PH domain containing 1 | FGD1 |
|  |  | Fibromodulin | FMOD |
|  |  | Golgin subfamily A conserved domain-containing protein | GOLGA2 |
|  |  | Hepcidin antimicrobial peptide | Hamp |
|  |  | Iduronate 2-sulfatase | IDS |
|  |  | Insulin-like growth factor-binding protein 4 | IGFBP4 |
|  |  | Interferon gamma receptor 1 | IFNGR1 |
|  |  | Keratin 10A | KRT10A |
|  |  | Leucine rich repeat and Ig domain containing 2 | LINGO2 |
|  |  | Multiple EGF like domains 10 | MEGF10 |
|  |  | Platelet derived growth factor subunit B | PDGFB |
|  |  | Pleckstrin homology, MyTH4 and FERM domain containing H2 | PLEKHH2 |
|  |  | Ribonuclease A family member 4 | RNASE4 |
|  |  | SLIT and NTRK like family member 1 | SLITRK1 |
|  |  | SWI/SNF related, matrix associated, actin dependent regulator of chromatin subfamily c member 1 | SMARCC1 |
|  |  | Seizure related 6 homolog | SEZ6 |
|  |  | Titin | TTN |
|  |  | Tripeptidyl-peptidase 1 | TPP1 |
| PreOmics only | 173 | 3~(2~), 5~-bisphosphate nucleotidase 2 | BPNT2 |
|  |  | 45 kDa calcium-binding protein | SDF4 |
|  |  | 5~-3~ exoribonuclease | XRN2 |
|  |  | 78 kDa glucose-regulated protein | HSPA5 |
|  |  | A-kinase anchoring protein 9 | AKAP9 |
|  |  | ABI family member 3 binding protein | ABI3BP |
|  |  | ADAM like decysin 1 | ADAMDEC1 |
|  |  | ADAM metallopeptidase domain 15 | ADAM15 |
|  |  | ADAM metallopeptidase with thrombospondin type 1 motif 3 | ADAMTS3 |
|  |  | AHNAK nucleoprotein | AHNAK |
|  |  | Adenylate cyclase type 5 | ADCY5 |
|  |  | Alpha-1,6-mannosyl-glycoprotein 2-beta-N-acetylglucosaminyltransferase | MGAT2 |
|  |  | Alpha-2-macroglobulin like 1 | A2ML1 |
|  |  | Alpha-fetoprotein | AFP |
|  |  | Alsin Rho guanine nucleotide exchange factor ALS2 | ALS2 |
|  |  | Annexin | ANXA1 |
|  |  | Apolipoprotein B | APOB |
|  |  | Arachidonate 12-lipoxygenase, 12R type | ALOX12B |
|  |  | Arginase | ARG1 |
|  |  | Beta-1,4-N-acetyl-galactosaminyltransferase 1 | B4GALNT1 |
|  |  | Bleomycin hydrolase | BLMH |
|  |  | Bone morphotic protein 3 | BMP3 |
|  |  | Brain abundant membrane attached signal protein 1 | BASP1 |
|  |  | C-type lectin domain containing 16A | CLEC16A |
|  |  | C1q and TNF related 5 | C1QTNF5 |
|  |  | Cadherin 4 | CDH4 |
|  |  | Calpain 1 | CAPN1 |
|  |  | Calpain small subunit 1 | CAPNS1 |
|  |  | Carbonic anhydrase | CA2 |
|  |  | Carboxypeptidase N subunit 1 | CPN1 |
|  |  | Carboxypeptidase N subunit 2 | CPN2 |
|  |  | Carboxypeptidase Q | CPQ |
|  |  | Cartilage oligomeric matrix protein | COMP |
|  |  | Catalase | CAT |
|  |  | Cathepsin H | LOC111775633 |
|  |  | Cathepsin S | CTSS |
|  |  | Cellular communication network factor 6 | CCN6 |
|  |  | Chitinase 3 like 1 | CHI3L1 |
|  |  | Coagulation factor IX | F9 |
|  |  | Collagen type III alpha 1 chain | COL3A1 |
|  |  | Collagen type VI alpha 2 chain | COL6A2 |
|  |  | Collagen type VI alpha 3 chain | COL6A3 |
|  |  | Collagen type XIV alpha 1 chain | COL14A1 |
|  |  | Contactin 4 | CNTN4 |
|  |  | Corneodesmosin | CDSN |
|  |  | Cystatin A | CSTA |
|  |  | DLC1 Rho GTPase activating protein | DLC1 |
|  |  | Desmocollin 1 | DSC1 |
|  |  | Desmoglein 1 | DSG1 |
|  |  | Desmoplakin | DSP |
|  |  | Dihydrolipoamide dehydrogenase | DLD |
|  |  | Discs large MAGUK scaffold protein 5 | DLG5 |
|  |  | EF-hand domain-containing protein | LOC100061699 |
|  |  | Ectopic P-granules 5 autophagy tethering factor | EPG5 |
|  |  | Elongation factor 1-alpha 1 | EEF1A1 |
|  |  | FAT atypical cadherin 1 | FAT1 |
|  |  | Family with sequence similarity 174 member A | FAM174A |
|  |  | Fatty acid binding protein 5 | FABP5 |
|  |  | Fibrinogen like 1 | FGL1 |
|  |  | Fibroblast growth factor receptor | FGFR1 |
|  |  | Fibrous sheath interacting protein 2 | FSIP2 |
|  |  | Fibrous sheath interacting protein 2 like | FSIP2L |
|  |  | Filamin A | FLNA |
|  |  | Follistatin like 4 | FSTL4 |
|  |  | GRIP and coiled-coil domain containing 2 | GCC2 |
|  |  | Gasdermin A | GSDMA |
|  |  | General vesicular transport factor p115 | USO1 |
|  |  | Glucosylceramidase | GBA1 |
|  |  | Glyceraldehyde-3-phosphate dehydrogenase | GAPDH |
|  |  | Glycine receptor alpha 2 | GLRA2 |
|  |  | H3.3 histone A | H3-3A |
|  |  | Heat shock protein 90 beta family member 1 | HSP90B1 |
|  |  | Heat shock protein beta-1 | HSPB1 |
|  |  | Heat shock protein family A (Hsp70) member 8 | HSPA8 |
|  |  | High mobility group AT-hook 1 | HMGA1 |
|  |  | Histidine ammonia-lyase | HAL |
|  |  | Histone H2B | H2BC7 |
|  |  | Histone H4 | H4C13 |
|  |  | Insulin like growth factor binding protein acid labile subunit | IGFALS |
|  |  | Interferon induced protein with tetratricopeptide repeats 2 | IFIT2 |
|  |  | Intraflagellar transport 140 | IFT140 |
|  |  | Junction plakoglobin | JUP |
|  |  | Kazal-like domain-containing protein | SPINK2 |
|  |  | Keratin 24 | KRT24 |
|  |  | Keratin 27 | KRT27 |
|  |  | Keratin 4 | KRT8 |
|  |  | Keratin 42 | KRT42 |
|  |  | Keratin 75 | KRT75 |
|  |  | Keratin 78 | KRT78 |
|  |  | Kinesin-like protein | KIF5C |
|  |  | L1 cell adhesion molecule | L1CAM |
|  |  | LDL receptor related protein 1 | LRP1 |
|  |  | LEM domain nuclear envelope protein 2 | LEMD2 |
|  |  | Lamin A/C | LMNA |
|  |  | Laminin subunit alpha 2 | LAMA2 |
|  |  | Laminin subunit beta 1 | LAMB1 |
|  |  | Laminin subunit gamma 1 | LAMC1 |
|  |  | Latent transforming growth factor beta binding protein 1 | LTBP1 |
|  |  | Lemur tyrosine kinase 3 | LMTK3 |
|  |  | Leucine rich repeat containing 4B | LRRC4B |
|  |  | Leukocyte cell derived chemotaxin 2 | LECT2 |
|  |  | Lymphocyte cytosolic protein 1 | LCP1 |
|  |  | Malate dehydrogenase | MDH2 |
|  |  | Mannose receptor C type 2 | MRC2 |
|  |  | Matrix remodeling-associated protein 8 | MXRA8 |
|  |  | Meteorin like, glial cell differentiation regulator | METRNL |
|  |  | Milk fat globule EGF and factor V/VIII domain containing | HAPLN3 |
|  |  | Myosin heavy chain 9 | MYH9 |
|  |  | N-acetylglucosamine-1-phosphate transferase subunit gamma | GNPTG |
|  |  | NME/NM23 nucleoside diphosphate kinase 1 | NME2 |
|  |  | Netrin G1 | NTNG1 |
|  |  | Nucleophosmin 1 | NPM1 |
|  |  | PATJ crumbs cell polarity complex component | PATJ |
|  |  | Palmitoyl-protein thioesterase 1 | PPT1 |
|  |  | Pentraxin family member | CRP |
|  |  | Peroxiredoxin-1 | N/A |
|  |  | Phosphoglycerate kinase | PGK1 |
|  |  | Plakophilin 1 | PKP1 |
|  |  | Platelet-derived growth factor (PDGF) family profile domain-containing protein | PDGFA |
|  |  | Plectin | PLEC |
|  |  | Polypeptide N-acetylgalactosaminyltransferase | GALNT10 |
|  |  | Pro-adrenomedullin | ADM |
|  |  | Proline and arginine rich end leucine rich repeat protein | PRELP |
|  |  | Prolylcarboxypeptidase | PRCP |
|  |  | Proteasome 20S subunit beta 1 | PSMB1 |
|  |  | Proteasome subunit alpha type | PSMA1 |
|  |  | Proteasome subunit beta | PSMB6 |
|  |  | Protein S100 | S100A11 |
|  |  | Protein disulfide-isomerase | PDIA3 |
|  |  | Protein tyrosine phosphatase receptor type N2 | PTPRN2 |
|  |  | Protocadherin 17 | PCDH17 |
|  |  | Protocadherin 7 | PCDH7 |
|  |  | Pyruvate kinase | PKM |
|  |  | RAD50 double strand break repair protein | RAD50 |
|  |  | RNA-binding protein FXR1 | FXR1 |
|  |  | Reticulon 4 receptor like 2 | RTN4RL2 |
|  |  | Ribonuclease T2 | N/A |
|  |  | Ring finger protein 214 | RNF214 |
|  |  | S100 calcium binding protein A14 | S100A14 |
|  |  | ST3 beta-galactoside alpha-2,3-sialyltransferase 6 | ST3GAL6 |
|  |  | STAM binding protein like 1 | STAMBPL1 |
|  |  | Secreted frizzled related protein 4 | SFRP4 |
|  |  | Semaphorin 4D | SEMA4D |
|  |  | Serpin domain-containing protein | LOC100057505 |
|  |  | Serpin family B member 12 | SERPINB12 |
|  |  | Serpin family B member 13 | SERPINB13 |
|  |  | Solute carrier family 12 member 7 | SLC12A7 |
|  |  | Solute carrier family 39 member 10 | SLC39A10 |
|  |  | Sortilin related VPS10 domain containing receptor 1 | SORCS1 |
|  |  | Spectrin repeat containing nuclear envelope protein 1 | SYNE1 |
|  |  | Spondin 2 | SPON2 |
|  |  | Sulfhydryl oxidase | QSOX1 |
|  |  | Sushi, nidogen and EGF like domains 1 | SNED1 |
|  |  | Transglutaminase 1 | TGM1 |
|  |  | Transglutaminase 3 | TGM3 |
|  |  | Transketolase | TKT |
|  |  | Transmembrane protein 132D | TMEM132D |
|  |  | Tudor domain containing 15 | TDRD15 |
|  |  | Tyrosine 3-monooxygenase/tryptophan 5-monooxygenase activation protein zeta | YWHAZ |
|  |  | Uteroglobin | SCGB1A1 |
|  |  | Uveal autoantigen with coiled-coil domains and ankyrin repeats | UACA |
|  |  | Vinculin | VCL |
|  |  | Voltage dependent anion channel 2 | VDAC2 |
|  |  | Xyloside xylosyltransferase 1 | XXYLT1 |
|  |  | Zinc finger and BTB domain containing 32 | ZBTB32 |
|  |  | alanine transaminase | GPT2 |
|  |  | creatine kinase | LOC100056283 |
|  |  | mitogen-activated protein kinase kinase kinase | MAP3K15 |
|  |  | phosphopyruvate hydratase | ENO1 |
|  |  | porphobilinogen synthase | ALAD |
|  |  | procollagen-lysine 5-dioxygenase | PLOD1 |
|  |  | protein deglycase | PARK7 |
|  |  | von Willebrand factor C domain containing 2 | VWC2 |

## Table S5. Pathway Overlaps Between Techniques

| **Overlap** | **Number of Pathways** | **Pathway Name** |
| --- | --- | --- |
| Native + ProteoMiner + PreOmics | 224 | B cell mediated immunity (GO:0019724) |
|  |  | RNA biosynthetic process (GO:0032774) |
|  |  | RNA metabolic process (GO:0016070) |
|  |  | Unclassified (UNCLASSIFIED) |
|  |  | activation of immune response (GO:0002253) |
|  |  | acute inflammatory response (GO:0002526) |
|  |  | acute-phase response (GO:0006953) |
|  |  | adaptive immune response (GO:0002250) |
|  |  | adaptive immune response based on somatic recombination of immune receptors built from immunoglobulin superfamily domains (GO:0002460) |
|  |  | ameboidal-type cell migration (GO:0001667) |
|  |  | anatomical structure development (GO:0048856) |
|  |  | anatomical structure morphogenesis (GO:0009653) |
|  |  | animal organ development (GO:0048513) |
|  |  | axon development (GO:0061564) |
|  |  | axon guidance (GO:0007411) |
|  |  | axonal fasciculation (GO:0007413) |
|  |  | axonogenesis (GO:0007409) |
|  |  | biological process involved in interspecies interaction between organisms (GO:0044419) |
|  |  | biological regulation (GO:0065007) |
|  |  | biological_process (GO:0008150) |
|  |  | blood coagulation (GO:0007596) |
|  |  | blood coagulation, fibrin clot formation (GO:0072378) |
|  |  | blood vessel development (GO:0001568) |
|  |  | blood vessel diameter maintenance (GO:0097746) |
|  |  | blood vessel morphogenesis (GO:0048514) |
|  |  | cell adhesion (GO:0007155) |
|  |  | cell differentiation (GO:0030154) |
|  |  | cell junction organization (GO:0034330) |
|  |  | cell killing (GO:0001906) |
|  |  | cell migration (GO:0016477) |
|  |  | cell morphogenesis (GO:0000902) |
|  |  | cell morphogenesis involved in neuron differentiation (GO:0048667) |
|  |  | cell motility (GO:0048870) |
|  |  | cell projection morphogenesis (GO:0048858) |
|  |  | cell projection organization (GO:0030030) |
|  |  | cell recognition (GO:0008037) |
|  |  | cell-cell adhesion (GO:0098609) |
|  |  | cellular detoxification (GO:1990748) |
|  |  | cellular developmental process (GO:0048869) |
|  |  | cellular oxidant detoxification (GO:0098869) |
|  |  | cellular response to calcium ion starvation (GO:0072732) |
|  |  | cellular response to toxic substance (GO:0097237) |
|  |  | central nervous system development (GO:0007417) |
|  |  | cholesterol transport (GO:0030301) |
|  |  | chylomicron remodeling (GO:0034371) |
|  |  | coagulation (GO:0050817) |
|  |  | collagen metabolic process (GO:0032963) |
|  |  | complement activation (GO:0006956) |
|  |  | complement activation, GZMK pathway (GO:0160257) |
|  |  | complement activation, alternative pathway (GO:0006957) |
|  |  | complement activation, classical pathway (GO:0006958) |
|  |  | cytolysis by host of symbiont cells (GO:0051838) |
|  |  | defense response (GO:0006952) |
|  |  | defense response to other organism (GO:0098542) |
|  |  | defense response to symbiont (GO:0140546) |
|  |  | detoxification (GO:0098754) |
|  |  | developmental process (GO:0032502) |
|  |  | disruption of anatomical structure in another organism (GO:0141060) |
|  |  | disruption of cell in another organism (GO:0141061) |
|  |  | external encapsulating structure organization (GO:0045229) |
|  |  | extracellular matrix assembly (GO:0085029) |
|  |  | extracellular matrix organization (GO:0030198) |
|  |  | extracellular structure organization (GO:0043062) |
|  |  | fibrinolysis (GO:0042730) |
|  |  | generation of neurons (GO:0048699) |
|  |  | hemostasis (GO:0007599) |
|  |  | homophilic cell-cell adhesion (GO:0007156) |
|  |  | humoral immune response (GO:0006959) |
|  |  | humoral immune response mediated by circulating immunoglobulin (GO:0002455) |
|  |  | immune effector process (GO:0002252) |
|  |  | immune response (GO:0006955) |
|  |  | immune system process (GO:0002376) |
|  |  | immunoglobulin mediated immune response (GO:0016064) |
|  |  | inflammatory response (GO:0006954) |
|  |  | innate immune response (GO:0045087) |
|  |  | intermediate filament organization (GO:0045109) |
|  |  | killing of cells of another organism (GO:0031640) |
|  |  | leukocyte mediated immunity (GO:0002443) |
|  |  | lipoprotein metabolic process (GO:0042157) |
|  |  | locomotory behavior (GO:0007626) |
|  |  | lymphocyte mediated immunity (GO:0002449) |
|  |  | multicellular organism development (GO:0007275) |
|  |  | multicellular organismal process (GO:0032501) |
|  |  | negative regulation of amide metabolic process (GO:0034249) |
|  |  | negative regulation of amyloid precursor protein catabolic process (GO:1902992) |
|  |  | negative regulation of amyloid-beta formation (GO:1902430) |
|  |  | negative regulation of biological process (GO:0048519) |
|  |  | negative regulation of blood coagulation (GO:0030195) |
|  |  | negative regulation of cell adhesion (GO:0007162) |
|  |  | negative regulation of coagulation (GO:0050819) |
|  |  | negative regulation of complement activation (GO:0045916) |
|  |  | negative regulation of complement activation, lectin pathway (GO:0001869) |
|  |  | negative regulation of fibrinolysis (GO:0051918) |
|  |  | negative regulation of hemostasis (GO:1900047) |
|  |  | negative regulation of humoral immune response (GO:0002921) |
|  |  | negative regulation of membrane depolarization (GO:1904180) |
|  |  | negative regulation of mitochondrial depolarization (GO:0051902) |
|  |  | negative regulation of multicellular organismal process (GO:0051241) |
|  |  | negative regulation of protein metabolic process (GO:0051248) |
|  |  | negative regulation of proteolysis (GO:0045861) |
|  |  | negative regulation of response to external stimulus (GO:0032102) |
|  |  | negative regulation of response to stimulus (GO:0048585) |
|  |  | negative regulation of response to wounding (GO:1903035) |
|  |  | negative regulation of wound healing (GO:0061045) |
|  |  | nervous system development (GO:0007399) |
|  |  | neurogenesis (GO:0022008) |
|  |  | neuron development (GO:0048666) |
|  |  | neuron projection development (GO:0031175) |
|  |  | neuron projection fasciculation (GO:0106030) |
|  |  | neuron projection guidance (GO:0097485) |
|  |  | neuron projection morphogenesis (GO:0048812) |
|  |  | neuron recognition (GO:0008038) |
|  |  | nucleic acid biosynthetic process (GO:0141187) |
|  |  | nucleic acid metabolic process (GO:0090304) |
|  |  | nucleobase-containing compound biosynthetic process (GO:0034654) |
|  |  | nucleobase-containing compound metabolic process (GO:0006139) |
|  |  | ossification (GO:0001503) |
|  |  | plasma lipoprotein particle organization (GO:0071827) |
|  |  | plasma lipoprotein particle remodeling (GO:0034369) |
|  |  | plasma membrane bounded cell projection morphogenesis (GO:0120039) |
|  |  | plasma membrane bounded cell projection organization (GO:0120036) |
|  |  | plasminogen activation (GO:0031639) |
|  |  | positive regulation of MAPK cascade (GO:0043410) |
|  |  | positive regulation of biological process (GO:0048518) |
|  |  | positive regulation of blood coagulation (GO:0030194) |
|  |  | positive regulation of cell adhesion (GO:0045785) |
|  |  | positive regulation of cell communication (GO:0010647) |
|  |  | positive regulation of cell-substrate adhesion (GO:0010811) |
|  |  | positive regulation of cellular process (GO:0048522) |
|  |  | positive regulation of coagulation (GO:0050820) |
|  |  | positive regulation of endocytosis (GO:0045807) |
|  |  | positive regulation of fibrinolysis (GO:0051919) |
|  |  | positive regulation of hemostasis (GO:1900048) |
|  |  | positive regulation of heterotypic cell-cell adhesion (GO:0034116) |
|  |  | positive regulation of immune response (GO:0050778) |
|  |  | positive regulation of immune system process (GO:0002684) |
|  |  | positive regulation of intracellular signal transduction (GO:1902533) |
|  |  | positive regulation of multicellular organismal process (GO:0051240) |
|  |  | positive regulation of phospholipid efflux (GO:1902995) |
|  |  | positive regulation of protein maturation (GO:1903319) |
|  |  | positive regulation of protein processing (GO:0010954) |
|  |  | positive regulation of response to stimulus (GO:0048584) |
|  |  | positive regulation of response to wounding (GO:1903036) |
|  |  | positive regulation of signal transduction (GO:0009967) |
|  |  | positive regulation of signaling (GO:0023056) |
|  |  | positive regulation of wound healing (GO:0090303) |
|  |  | protein activation cascade (GO:0072376) |
|  |  | protein maturation (GO:0051604) |
|  |  | protein metabolic process (GO:0019538) |
|  |  | protein processing (GO:0016485) |
|  |  | protein-containing complex remodeling (GO:0034367) |
|  |  | protein-lipid complex remodeling (GO:0034368) |
|  |  | proteolysis (GO:0006508) |
|  |  | regulation of ERK1 and ERK2 cascade (GO:0070372) |
|  |  | regulation of MAPK cascade (GO:0043408) |
|  |  | regulation of amyloid precursor protein catabolic process (GO:1902991) |
|  |  | regulation of amyloid-beta formation (GO:1902003) |
|  |  | regulation of anatomical structure morphogenesis (GO:0022603) |
|  |  | regulation of anatomical structure size (GO:0090066) |
|  |  | regulation of biological quality (GO:0065008) |
|  |  | regulation of blood coagulation (GO:0030193) |
|  |  | regulation of blood vessel remodeling (GO:0060312) |
|  |  | regulation of body fluid levels (GO:0050878) |
|  |  | regulation of cell adhesion (GO:0030155) |
|  |  | regulation of cell communication (GO:0010646) |
|  |  | regulation of cell growth (GO:0001558) |
|  |  | regulation of cell migration (GO:0030334) |
|  |  | regulation of cell motility (GO:2000145) |
|  |  | regulation of cell population proliferation (GO:0042127) |
|  |  | regulation of cell-cell adhesion (GO:0022407) |
|  |  | regulation of cell-substrate adhesion (GO:0010810) |
|  |  | regulation of cellular component organization (GO:0051128) |
|  |  | regulation of coagulation (GO:0050818) |
|  |  | regulation of collagen fibril organization (GO:1904026) |
|  |  | regulation of complement activation (GO:0030449) |
|  |  | regulation of complement activation, lectin pathway (GO:0001868) |
|  |  | regulation of fibrinolysis (GO:0051917) |
|  |  | regulation of hemostasis (GO:1900046) |
|  |  | regulation of heterotypic cell-cell adhesion (GO:0034114) |
|  |  | regulation of hormone levels (GO:0010817) |
|  |  | regulation of humoral immune response (GO:0002920) |
|  |  | regulation of immune response (GO:0050776) |
|  |  | regulation of immune system process (GO:0002682) |
|  |  | regulation of locomotion (GO:0040012) |
|  |  | regulation of mitochondrial depolarization (GO:0051900) |
|  |  | regulation of multicellular organismal process (GO:0051239) |
|  |  | regulation of phospholipid efflux (GO:1902994) |
|  |  | regulation of protein maturation (GO:1903317) |
|  |  | regulation of protein metabolic process (GO:0051246) |
|  |  | regulation of protein processing (GO:0070613) |
|  |  | regulation of proteolysis (GO:0030162) |
|  |  | regulation of response to external stimulus (GO:0032101) |
|  |  | regulation of response to stimulus (GO:0048583) |
|  |  | regulation of response to stress (GO:0080134) |
|  |  | regulation of response to wounding (GO:1903034) |
|  |  | regulation of signal transduction (GO:0009966) |
|  |  | regulation of signaling (GO:0023051) |
|  |  | regulation of synapse organization (GO:0050807) |
|  |  | regulation of synapse structure or activity (GO:0050803) |
|  |  | regulation of tube diameter (GO:0035296) |
|  |  | regulation of tube size (GO:0035150) |
|  |  | regulation of wound healing (GO:0061041) |
|  |  | response to amyloid-beta (GO:1904645) |
|  |  | response to biotic stimulus (GO:0009607) |
|  |  | response to dietary excess (GO:0002021) |
|  |  | response to external biotic stimulus (GO:0043207) |
|  |  | response to external stimulus (GO:0009605) |
|  |  | response to other organism (GO:0051707) |
|  |  | response to oxidative stress (GO:0006979) |
|  |  | response to reactive oxygen species (GO:0000302) |
|  |  | response to stimulus (GO:0050896) |
|  |  | response to stress (GO:0006950) |
|  |  | response to toxic substance (GO:0009636) |
|  |  | response to wounding (GO:0009611) |
|  |  | reverse cholesterol transport (GO:0043691) |
|  |  | sterol transport (GO:0015918) |
|  |  | synapse organization (GO:0050808) |
|  |  | system development (GO:0048731) |
|  |  | tissue development (GO:0009888) |
|  |  | triglyceride-rich lipoprotein particle remodeling (GO:0034370) |
|  |  | vasculature development (GO:0001944) |
|  |  | very-low-density lipoprotein particle remodeling (GO:0034372) |
|  |  | wound healing (GO:0042060) |
|  |  | zymogen activation (GO:0031638) |
| Native + ProteoMiner | 14 | behavior (GO:0007610) |
|  |  | cell adhesion mediated by integrin (GO:0033627) |
|  |  | cellular response to oxygen radical (GO:0071450) |
|  |  | cellular response to superoxide (GO:0071451) |
|  |  | elastic fiber assembly (GO:0048251) |
|  |  | positive regulation of ERK1 and ERK2 cascade (GO:0070374) |
|  |  | positive regulation of cell population proliferation (GO:0008284) |
|  |  | positive regulation of cell-cell adhesion (GO:0022409) |
|  |  | regulation of activated T cell proliferation (GO:0046006) |
|  |  | regulation of biological process (GO:0050789) |
|  |  | regulation of collagen metabolic process (GO:0010712) |
|  |  | regulation of triglyceride metabolic process (GO:0090207) |
|  |  | removal of superoxide radicals (GO:0019430) |
|  |  | synapse pruning (GO:0098883) |
| Native + PreOmics | 60 | RNA processing (GO:0006396) |
|  |  | blood circulation (GO:0008015) |
|  |  | bone development (GO:0060348) |
|  |  | cell cycle (GO:0007049) |
|  |  | cellular process (GO:0009987) |
|  |  | cellular response to amyloid-beta (GO:1904646) |
|  |  | cellular response to chemical stimulus (GO:0070887) |
|  |  | cellular response to oxygen-containing compound (GO:1901701) |
|  |  | circulatory system process (GO:0003013) |
|  |  | detection of chemical stimulus (GO:0009593) |
|  |  | detection of chemical stimulus involved in sensory perception (GO:0050907) |
|  |  | detection of chemical stimulus involved in sensory perception of smell (GO:0050911) |
|  |  | detection of stimulus involved in sensory perception (GO:0050906) |
|  |  | endocrine process (GO:0050886) |
|  |  | epidermal cell differentiation (GO:0009913) |
|  |  | epidermis development (GO:0008544) |
|  |  | glucose metabolic process (GO:0006006) |
|  |  | hexose metabolic process (GO:0019318) |
|  |  | high-density lipoprotein particle remodeling (GO:0034375) |
|  |  | hyaluronan metabolic process (GO:0030212) |
|  |  | intermediate filament cytoskeleton organization (GO:0045104) |
|  |  | intermediate filament-based process (GO:0045103) |
|  |  | monosaccharide metabolic process (GO:0005996) |
|  |  | negative regulation of cell communication (GO:0010648) |
|  |  | negative regulation of cell motility (GO:2000146) |
|  |  | negative regulation of developmental process (GO:0051093) |
|  |  | negative regulation of locomotion (GO:0040013) |
|  |  | negative regulation of signal transduction (GO:0009968) |
|  |  | negative regulation of signaling (GO:0023057) |
|  |  | neuron differentiation (GO:0030182) |
|  |  | neuronal ion channel clustering (GO:0045161) |
|  |  | osteoblast differentiation (GO:0001649) |
|  |  | positive regulation of cholesterol efflux (GO:0010875) |
|  |  | positive regulation of cholesterol transport (GO:0032376) |
|  |  | positive regulation of lipid localization (GO:1905954) |
|  |  | positive regulation of lipid transport (GO:0032370) |
|  |  | positive regulation of sterol transport (GO:0032373) |
|  |  | positive regulation of transport (GO:0051050) |
|  |  | protein-lipid complex organization (GO:0071825) |
|  |  | regulation of cell junction assembly (GO:1901888) |
|  |  | regulation of cholesterol efflux (GO:0010874) |
|  |  | regulation of cholesterol transport (GO:0032374) |
|  |  | regulation of developmental process (GO:0050793) |
|  |  | regulation of growth (GO:0040008) |
|  |  | regulation of lipid localization (GO:1905952) |
|  |  | regulation of lipid transport (GO:0032368) |
|  |  | regulation of localization (GO:0032879) |
|  |  | regulation of mitochondrial membrane potential (GO:0051881) |
|  |  | regulation of nucleobase-containing compound metabolic process (GO:0019219) |
|  |  | regulation of plasma lipoprotein particle levels (GO:0097006) |
|  |  | regulation of postsynapse organization (GO:0099175) |
|  |  | regulation of sterol transport (GO:0032371) |
|  |  | regulation of synapse assembly (GO:0051963) |
|  |  | regulation of system process (GO:0044057) |
|  |  | regulation of transport (GO:0051049) |
|  |  | response to metal ion (GO:0010038) |
|  |  | sensory perception of chemical stimulus (GO:0007606) |
|  |  | sensory perception of smell (GO:0007608) |
|  |  | skeletal system development (GO:0001501) |
|  |  | skin development (GO:0043588) |
| ProteoMiner + PreOmics | 27 | DNA damage response (GO:0006974) |
|  |  | DNA metabolic process (GO:0006259) |
|  |  | anatomical structure formation involved in morphogenesis (GO:0048646) |
|  |  | angiogenesis (GO:0001525) |
|  |  | biological process involved in interaction with symbiont (GO:0051702) |
|  |  | blood coagulation, common pathway (GO:0072377) |
|  |  | cell-matrix adhesion (GO:0007160) |
|  |  | cell-substrate adhesion (GO:0031589) |
|  |  | cellular component organization (GO:0016043) |
|  |  | circulatory system development (GO:0072359) |
|  |  | collagen fibril organization (GO:0030199) |
|  |  | dendrite self-avoidance (GO:0070593) |
|  |  | developmental maturation (GO:0021700) |
|  |  | endoderm development (GO:0007492) |
|  |  | endoderm formation (GO:0001706) |
|  |  | endodermal cell differentiation (GO:0035987) |
|  |  | head development (GO:0060322) |
|  |  | homotypic cell-cell adhesion (GO:0034109) |
|  |  | induction of bacterial agglutination (GO:0043152) |
|  |  | integrin-mediated signaling pathway (GO:0007229) |
|  |  | platelet activation (GO:0030168) |
|  |  | positive regulation of cellular component organization (GO:0051130) |
|  |  | regulation of axonogenesis (GO:0050770) |
|  |  | regulation of neuron projection development (GO:0010975) |
|  |  | response to nutrient levels (GO:0031667) |
|  |  | tube morphogenesis (GO:0035239) |
|  |  | vascular process in circulatory system (GO:0003018) |
| Native only | 34 | carbohydrate metabolic process (GO:0005975) |
|  |  | cellular response to oxidative stress (GO:0034599) |
|  |  | cellular response to reactive oxygen species (GO:0034614) |
|  |  | glial cell development (GO:0021782) |
|  |  | glial cell differentiation (GO:0010001) |
|  |  | gliogenesis (GO:0042063) |
|  |  | hormone secretion (GO:0046879) |
|  |  | leukocyte degranulation (GO:0043299) |
|  |  | mast cell activation (GO:0045576) |
|  |  | mast cell activation involved in immune response (GO:0002279) |
|  |  | mast cell chemotaxis (GO:0002551) |
|  |  | mast cell degranulation (GO:0043303) |
|  |  | mast cell mediated immunity (GO:0002448) |
|  |  | mast cell migration (GO:0097531) |
|  |  | modulation of chemical synaptic transmission (GO:0050804) |
|  |  | myeloid leukocyte mediated immunity (GO:0002444) |
|  |  | polarity specification of anterior/posterior axis (GO:0009949) |
|  |  | positive regulation of activated T cell proliferation (GO:0042104) |
|  |  | positive regulation of cerebellar granule cell precursor proliferation (GO:0021940) |
|  |  | positive regulation of phagocytosis (GO:0050766) |
|  |  | positive regulation of smooth muscle cell differentiation (GO:0051152) |
|  |  | reactive oxygen species metabolic process (GO:0072593) |
|  |  | regulation of astrocyte differentiation (GO:0048710) |
|  |  | regulation of behavioral fear response (GO:2000822) |
|  |  | regulation of fear response (GO:1903365) |
|  |  | regulation of glial cell differentiation (GO:0045685) |
|  |  | regulation of membrane depolarization (GO:0003254) |
|  |  | regulation of phagocytosis (GO:0050764) |
|  |  | regulation of protein localization (GO:0032880) |
|  |  | regulation of synaptic plasticity (GO:0048167) |
|  |  | regulation of trans-synaptic signaling (GO:0099177) |
|  |  | response to oxygen radical (GO:0000305) |
|  |  | response to superoxide (GO:0000303) |
|  |  | specification of axis polarity (GO:0065001) |
| ProteoMiner only | 19 | AMPA glutamate receptor clustering (GO:0097113) |
|  |  | acylglycerol homeostasis (GO:0055090) |
|  |  | adult behavior (GO:0030534) |
|  |  | adult locomotory behavior (GO:0008344) |
|  |  | cell junction disassembly (GO:0150146) |
|  |  | glutamate receptor clustering (GO:0097688) |
|  |  | high-density lipoprotein particle assembly (GO:0034380) |
|  |  | locomotion (GO:0040011) |
|  |  | negative regulation of extrinsic apoptotic signaling pathway via death domain receptors (GO:1902042) |
|  |  | positive regulation of phospholipid transport (GO:2001140) |
|  |  | positive regulation of vasoconstriction (GO:0045907) |
|  |  | postsynapse organization (GO:0099173) |
|  |  | postsynaptic membrane organization (GO:0001941) |
|  |  | regeneration (GO:0031099) |
|  |  | regulation of insulin-like growth factor receptor signaling pathway (GO:0043567) |
|  |  | regulation of phospholipid transport (GO:2001138) |
|  |  | regulation of smooth muscle cell proliferation (GO:0048660) |
|  |  | synapse maturation (GO:0060074) |
|  |  | tissue regeneration (GO:0042246) |
| PreOmics only | 143 | ADP catabolic process (GO:0046032) |
|  |  | ADP metabolic process (GO:0046031) |
|  |  | ATP metabolic process (GO:0046034) |
|  |  | DNA repair (GO:0006281) |
|  |  | G protein-coupled receptor signaling pathway (GO:0007186) |
|  |  | adherens junction organization (GO:0034332) |
|  |  | aerobic respiration (GO:0009060) |
|  |  | animal organ morphogenesis (GO:0009887) |
|  |  | basement membrane organization (GO:0071711) |
|  |  | biomineral tissue development (GO:0031214) |
|  |  | bone trabecula morphogenesis (GO:0061430) |
|  |  | brain development (GO:0007420) |
|  |  | bundle of His cell to Purkinje myocyte communication (GO:0086069) |
|  |  | carbohydrate catabolic process (GO:0016052) |
|  |  | carbohydrate derivative catabolic process (GO:1901136) |
|  |  | carbohydrate derivative metabolic process (GO:1901135) |
|  |  | catabolic process (GO:0009056) |
|  |  | cell cycle process (GO:0022402) |
|  |  | cell development (GO:0048468) |
|  |  | cell junction assembly (GO:0034329) |
|  |  | cell-cell adhesion mediated by cadherin (GO:0044331) |
|  |  | cell-cell junction assembly (GO:0007043) |
|  |  | cell-cell junction organization (GO:0045216) |
|  |  | cellular component organization or biogenesis (GO:0071840) |
|  |  | cellular respiration (GO:0045333) |
|  |  | cellular response to amino acid stimulus (GO:0071230) |
|  |  | chaperone-mediated autophagy (GO:0061684) |
|  |  | cholesterol efflux (GO:0033344) |
|  |  | chromatin organization (GO:0006325) |
|  |  | collagen biosynthetic process (GO:0032964) |
|  |  | desmosome maintenance (GO:0002160) |
|  |  | desmosome organization (GO:0002934) |
|  |  | detection of stimulus (GO:0051606) |
|  |  | energy homeostasis (GO:0097009) |
|  |  | epithelial cell differentiation (GO:0030855) |
|  |  | epithelium development (GO:0060429) |
|  |  | establishment of protein localization to extracellular region (GO:0035592) |
|  |  | gluconeogenesis (GO:0006094) |
|  |  | glucose catabolic process (GO:0006007) |
|  |  | glycolytic process (GO:0006096) |
|  |  | homeostatic process (GO:0042592) |
|  |  | hydrogen peroxide catabolic process (GO:0042744) |
|  |  | hydroxylysine biosynthetic process (GO:0046947) |
|  |  | keratinization (GO:0031424) |
|  |  | keratinocyte differentiation (GO:0030216) |
|  |  | kinin cascade (GO:0002254) |
|  |  | lipoprotein biosynthetic process (GO:0042158) |
|  |  | lipoprotein catabolic process (GO:0042159) |
|  |  | macromolecule localization (GO:0033036) |
|  |  | macromolecule metabolic process (GO:0043170) |
|  |  | maintenance of location (GO:0051235) |
|  |  | mesenchymal cell differentiation (GO:0048762) |
|  |  | mesenchymal cell migration (GO:0090497) |
|  |  | metabolic process (GO:0008152) |
|  |  | motor neuron axon guidance (GO:0008045) |
|  |  | negative regulation of cell migration (GO:0030336) |
|  |  | negative regulation of cell population proliferation (GO:0008285) |
|  |  | negative regulation of cell projection organization (GO:0031345) |
|  |  | negative regulation of cell-cell adhesion (GO:0022408) |
|  |  | negative regulation of cell-substrate adhesion (GO:0010812) |
|  |  | negative regulation of cellular component organization (GO:0051129) |
|  |  | negative regulation of cellular process (GO:0048523) |
|  |  | negative regulation of cytokine production (GO:0001818) |
|  |  | negative regulation of defense response (GO:0031348) |
|  |  | negative regulation of endothelial cell migration (GO:0010596) |
|  |  | negative regulation of immune effector process (GO:0002698) |
|  |  | negative regulation of immune system process (GO:0002683) |
|  |  | negative regulation of long-term synaptic potentiation (GO:1900272) |
|  |  | neural crest cell development (GO:0014032) |
|  |  | neuroblast proliferation (GO:0007405) |
|  |  | nicotinamide nucleotide metabolic process (GO:0046496) |
|  |  | nucleoside diphosphate catabolic process (GO:0009134) |
|  |  | nucleoside diphosphate metabolic process (GO:0009132) |
|  |  | nucleoside phosphate catabolic process (GO:1901292) |
|  |  | nucleoside triphosphate metabolic process (GO:0009141) |
|  |  | nucleotide catabolic process (GO:0009166) |
|  |  | plasma kallikrein-kinin cascade (GO:0002353) |
|  |  | platelet aggregation (GO:0070527) |
|  |  | positive regulation of Rho protein signal transduction (GO:0035025) |
|  |  | positive regulation of cell differentiation (GO:0045597) |
|  |  | positive regulation of cell junction assembly (GO:1901890) |
|  |  | positive regulation of cell migration (GO:0030335) |
|  |  | positive regulation of cell motility (GO:2000147) |
|  |  | positive regulation of cell-matrix adhesion (GO:0001954) |
|  |  | positive regulation of developmental process (GO:0051094) |
|  |  | positive regulation of hormone secretion (GO:0046887) |
|  |  | positive regulation of keratinocyte apoptotic process (GO:1902174) |
|  |  | positive regulation of locomotion (GO:0040017) |
|  |  | positive regulation of nervous system development (GO:0051962) |
|  |  | positive regulation of programmed cell death (GO:0043068) |
|  |  | positive regulation of protein metabolic process (GO:0051247) |
|  |  | positive regulation of protein processing in phagocytic vesicle (GO:1903923) |
|  |  | positive regulation of proteolysis (GO:0045862) |
|  |  | protein localization to cell junction (GO:1902414) |
|  |  | protein localization to extracellular region (GO:0071692) |
|  |  | protein refolding (GO:0042026) |
|  |  | protein targeting to lysosome involved in chaperone-mediated autophagy (GO:0061740) |
|  |  | purine nucleoside diphosphate catabolic process (GO:0009137) |
|  |  | purine nucleoside diphosphate metabolic process (GO:0009135) |
|  |  | purine nucleoside triphosphate metabolic process (GO:0009144) |
|  |  | purine nucleotide catabolic process (GO:0006195) |
|  |  | purine ribonucleoside diphosphate catabolic process (GO:0009181) |
|  |  | purine ribonucleoside diphosphate metabolic process (GO:0009179) |
|  |  | purine ribonucleoside triphosphate metabolic process (GO:0009205) |
|  |  | purine ribonucleotide catabolic process (GO:0009154) |
|  |  | purine ribonucleotide metabolic process (GO:0009150) |
|  |  | purine-containing compound catabolic process (GO:0072523) |
|  |  | pyridine nucleotide catabolic process (GO:0019364) |
|  |  | pyridine-containing compound catabolic process (GO:0072526) |
|  |  | pyridine-containing compound metabolic process (GO:0072524) |
|  |  | pyruvate metabolic process (GO:0006090) |
|  |  | regulation of DNA-templated transcription (GO:0006355) |
|  |  | regulation of RNA biosynthetic process (GO:2001141) |
|  |  | regulation of RNA metabolic process (GO:0051252) |
|  |  | regulation of Rho protein signal transduction (GO:0035023) |
|  |  | regulation of apoptotic process (GO:0042981) |
|  |  | regulation of cell cycle (GO:0051726) |
|  |  | regulation of cell cycle process (GO:0010564) |
|  |  | regulation of cell differentiation (GO:0045595) |
|  |  | regulation of cell-matrix adhesion (GO:0001952) |
|  |  | regulation of cellular response to growth factor stimulus (GO:0090287) |
|  |  | regulation of defense response (GO:0031347) |
|  |  | regulation of endocytosis (GO:0030100) |
|  |  | regulation of endothelial cell migration (GO:0010594) |
|  |  | regulation of extent of cell growth (GO:0061387) |
|  |  | regulation of extracellular matrix organization (GO:1903053) |
|  |  | regulation of hormone secretion (GO:0046883) |
|  |  | regulation of multicellular organismal development (GO:2000026) |
|  |  | regulation of nervous system development (GO:0051960) |
|  |  | regulation of plasminogen activation (GO:0010755) |
|  |  | regulation of postsynaptic density organization (GO:1905874) |
|  |  | regulation of postsynaptic membrane neurotransmitter receptor levels (GO:0099072) |
|  |  | regulation of programmed cell death (GO:0043067) |
|  |  | regulation of protein catabolic process (GO:0042176) |
|  |  | regulation of protein processing in phagocytic vesicle (GO:1903921) |
|  |  | regulation of transcription by RNA polymerase II (GO:0006357) |
|  |  | ribonucleoside diphosphate catabolic process (GO:0009191) |
|  |  | ribonucleoside diphosphate metabolic process (GO:0009185) |
|  |  | ribonucleotide catabolic process (GO:0009261) |
|  |  | sensory perception (GO:0007600) |
|  |  | skin morphogenesis (GO:0043589) |
|  |  | supramolecular fiber organization (GO:0097435) |
|  |  | tube development (GO:0035295) |

## Table S6. Pathways Unique to Native Technique

Total pathways unique to Native: 34

| **Pathway Name** | **FDR** |
| --- | --- |
| myeloid leukocyte mediated immunity (GO:0002444) | 0.00233 |
| carbohydrate metabolic process (GO:0005975) | 0.00416 |
| mast cell degranulation (GO:0043303) | 0.00435 |
| mast cell activation involved in immune response (GO:0002279) | 0.00437 |
| cellular response to reactive oxygen species (GO:0034614) | 0.00534 |
| mast cell mediated immunity (GO:0002448) | 0.00548 |
| leukocyte degranulation (GO:0043299) | 0.00705 |
| glial cell differentiation (GO:0010001) | 0.00832 |
| response to superoxide (GO:0000303) | 0.00888 |
| response to oxygen radical (GO:0000305) | 0.00892 |
| mast cell activation (GO:0045576) | 0.00905 |
| positive regulation of activated T cell proliferation (GO:0042104) | 0.01080 |
| regulation of astrocyte differentiation (GO:0048710) | 0.01320 |
| mast cell migration (GO:0097531) | 0.01580 |
| regulation of synaptic plasticity (GO:0048167) | 0.01590 |
| polarity specification of anterior/posterior axis (GO:0009949) | 0.01600 |
| mast cell chemotaxis (GO:0002551) | 0.01630 |
| regulation of glial cell differentiation (GO:0045685) | 0.01780 |
| regulation of trans-synaptic signaling (GO:0099177) | 0.01990 |
| modulation of chemical synaptic transmission (GO:0050804) | 0.02000 |
| regulation of protein localization (GO:0032880) | 0.02700 |
| gliogenesis (GO:0042063) | 0.02720 |
| regulation of phagocytosis (GO:0050764) | 0.02950 |
| regulation of fear response (GO:1903365) | 0.03740 |
| positive regulation of cerebellar granule cell precursor proliferation (GO:0021940) | 0.03770 |
| specification of axis polarity (GO:0065001) | 0.03820 |
| regulation of behavioral fear response (GO:2000822) | 0.03850 |
| glial cell development (GO:0021782) | 0.03860 |
| hormone secretion (GO:0046879) | 0.04210 |
| positive regulation of phagocytosis (GO:0050766) | 0.04220 |
| positive regulation of smooth muscle cell differentiation (GO:0051152) | 0.04240 |
| cellular response to oxidative stress (GO:0034599) | 0.04630 |
| reactive oxygen species metabolic process (GO:0072593) | 0.04730 |
| regulation of membrane depolarization (GO:0003254) | 0.04780 |

## Table S7. Pathways Unique to ProteoMiner Technique

Total pathways unique to ProteoMiner: 19

| **Pathway Name** | **FDR** |
| --- | --- |
| synapse maturation (GO:0060074) | 0.000263 |
| adult behavior (GO:0030534) | 0.001300 |
| adult locomotory behavior (GO:0008344) | 0.005970 |
| regeneration (GO:0031099) | 0.018200 |
| regulation of smooth muscle cell proliferation (GO:0048660) | 0.023900 |
| locomotion (GO:0040011) | 0.024500 |
| AMPA glutamate receptor clustering (GO:0097113) | 0.028100 |
| glutamate receptor clustering (GO:0097688) | 0.028500 |
| acylglycerol homeostasis (GO:0055090) | 0.028500 |
| tissue regeneration (GO:0042246) | 0.031300 |
| positive regulation of vasoconstriction (GO:0045907) | 0.038500 |
| regulation of insulin-like growth factor receptor signaling pathway (GO:0043567) | 0.038700 |
| negative regulation of extrinsic apoptotic signaling pathway via death domain receptors (GO:1902042) | 0.038800 |
| postsynaptic membrane organization (GO:0001941) | 0.039200 |
| postsynapse organization (GO:0099173) | 0.045800 |
| high-density lipoprotein particle assembly (GO:0034380) | 0.048700 |
| cell junction disassembly (GO:0150146) | 0.048900 |
| regulation of phospholipid transport (GO:2001138) | 0.049000 |
| positive regulation of phospholipid transport (GO:2001140) | 0.049200 |

## Table S8. Pathways Unique to PreOmics Technique

Total pathways unique to PreOmics: 143

| **Pathway Name** | **FDR** |
| --- | --- |
| keratinocyte differentiation (GO:0030216) | 0.00000127 |
| epithelial cell differentiation (GO:0030855) | 0.00000453 |
| supramolecular fiber organization (GO:0097435) | 0.00001370 |
| pyruvate metabolic process (GO:0006090) | 0.00006590 |
| epithelium development (GO:0060429) | 0.00009540 |
| regulation of programmed cell death (GO:0043067) | 0.00021700 |
| detection of stimulus (GO:0051606) | 0.00026400 |
| cellular component organization or biogenesis (GO:0071840) | 0.00026800 |
| glycolytic process (GO:0006096) | 0.00039900 |
| carbohydrate derivative metabolic process (GO:1901135) | 0.00042200 |
| ADP catabolic process (GO:0046032) | 0.00046400 |
| regulation of multicellular organismal development (GO:2000026) | 0.00054000 |
| ADP metabolic process (GO:0046031) | 0.00061100 |
| purine nucleoside diphosphate catabolic process (GO:0009137) | 0.00070700 |
| purine ribonucleoside diphosphate catabolic process (GO:0009181) | 0.00071000 |
| keratinization (GO:0031424) | 0.00072000 |
| pyridine nucleotide catabolic process (GO:0019364) | 0.00080600 |
| ribonucleoside diphosphate catabolic process (GO:0009191) | 0.00089200 |
| pyridine-containing compound catabolic process (GO:0072526) | 0.00101000 |
| purine nucleoside diphosphate metabolic process (GO:0009135) | 0.00102000 |
| nucleoside diphosphate catabolic process (GO:0009134) | 0.00102000 |
| purine ribonucleoside diphosphate metabolic process (GO:0009179) | 0.00103000 |
| desmosome organization (GO:0002934) | 0.00165000 |
| regulation of apoptotic process (GO:0042981) | 0.00174000 |
| cell-cell junction organization (GO:0045216) | 0.00175000 |
| cell development (GO:0048468) | 0.00216000 |
| ribonucleoside diphosphate metabolic process (GO:0009185) | 0.00231000 |
| carbohydrate catabolic process (GO:0016052) | 0.00238000 |
| platelet aggregation (GO:0070527) | 0.00261000 |
| regulation of RNA metabolic process (GO:0051252) | 0.00294000 |
| positive regulation of developmental process (GO:0051094) | 0.00297000 |
| nucleoside diphosphate metabolic process (GO:0009132) | 0.00354000 |
| purine ribonucleotide catabolic process (GO:0009154) | 0.00355000 |
| negative regulation of cell-substrate adhesion (GO:0010812) | 0.00477000 |
| ribonucleotide catabolic process (GO:0009261) | 0.00484000 |
| lipoprotein biosynthetic process (GO:0042158) | 0.00520000 |
| negative regulation of long-term synaptic potentiation (GO:1900272) | 0.00522000 |
| adherens junction organization (GO:0034332) | 0.00541000 |
| negative regulation of cellular component organization (GO:0051129) | 0.00561000 |
| cell junction assembly (GO:0034329) | 0.00569000 |
| purine-containing compound catabolic process (GO:0072523) | 0.00614000 |
| positive regulation of cell differentiation (GO:0045597) | 0.00614000 |
| cell-cell junction assembly (GO:0007043) | 0.00626000 |
| brain development (GO:0007420) | 0.00646000 |
| regulation of cell cycle (GO:0051726) | 0.00680000 |
| catabolic process (GO:0009056) | 0.00682000 |
| purine nucleotide catabolic process (GO:0006195) | 0.00739000 |
| purine ribonucleoside triphosphate metabolic process (GO:0009205) | 0.00749000 |
| basement membrane organization (GO:0071711) | 0.00868000 |
| regulation of Rho protein signal transduction (GO:0035023) | 0.00920000 |
| purine nucleoside triphosphate metabolic process (GO:0009144) | 0.00966000 |
| ATP metabolic process (GO:0046034) | 0.01000000 |
| regulation of DNA-templated transcription (GO:0006355) | 0.01130000 |
| regulation of RNA biosynthetic process (GO:2001141) | 0.01130000 |
| positive regulation of locomotion (GO:0040017) | 0.01200000 |
| regulation of transcription by RNA polymerase II (GO:0006357) | 0.01210000 |
| DNA repair (GO:0006281) | 0.01210000 |
| energy homeostasis (GO:0097009) | 0.01230000 |
| biomineral tissue development (GO:0031214) | 0.01360000 |
| positive regulation of cell migration (GO:0030335) | 0.01360000 |
| negative regulation of defense response (GO:0031348) | 0.01370000 |
| hydrogen peroxide catabolic process (GO:0042744) | 0.01430000 |
| negative regulation of immune system process (GO:0002683) | 0.01430000 |
| nicotinamide nucleotide metabolic process (GO:0046496) | 0.01470000 |
| metabolic process (GO:0008152) | 0.01520000 |
| sensory perception (GO:0007600) | 0.01610000 |
| positive regulation of programmed cell death (GO:0043068) | 0.01620000 |
| regulation of hormone secretion (GO:0046883) | 0.01700000 |
| positive regulation of Rho protein signal transduction (GO:0035025) | 0.01740000 |
| mesenchymal cell migration (GO:0090497) | 0.01750000 |
| regulation of defense response (GO:0031347) | 0.01750000 |
| protein localization to cell junction (GO:1902414) | 0.01760000 |
| positive regulation of protein processing in phagocytic vesicle (GO:1903923) | 0.01770000 |
| regulation of protein processing in phagocytic vesicle (GO:1903921) | 0.01770000 |
| establishment of protein localization to extracellular region (GO:0035592) | 0.01770000 |
| hydroxylysine biosynthetic process (GO:0046947) | 0.01780000 |
| pyridine-containing compound metabolic process (GO:0072524) | 0.01790000 |
| carbohydrate derivative catabolic process (GO:1901136) | 0.01790000 |
| kinin cascade (GO:0002254) | 0.01800000 |
| desmosome maintenance (GO:0002160) | 0.01800000 |
| plasma kallikrein-kinin cascade (GO:0002353) | 0.01820000 |
| nucleotide catabolic process (GO:0009166) | 0.01820000 |
| mesenchymal cell differentiation (GO:0048762) | 0.01910000 |
| regulation of endothelial cell migration (GO:0010594) | 0.01910000 |
| positive regulation of cell motility (GO:2000147) | 0.01940000 |
| skin morphogenesis (GO:0043589) | 0.01970000 |
| cell-cell adhesion mediated by cadherin (GO:0044331) | 0.01970000 |
| negative regulation of cell migration (GO:0030336) | 0.01970000 |
| regulation of postsynaptic density organization (GO:1905874) | 0.01980000 |
| protein refolding (GO:0042026) | 0.02000000 |
| positive regulation of proteolysis (GO:0045862) | 0.02000000 |
| regulation of cell differentiation (GO:0045595) | 0.02000000 |
| G protein-coupled receptor signaling pathway (GO:0007186) | 0.02000000 |
| regulation of nervous system development (GO:0051960) | 0.02130000 |
| nucleoside triphosphate metabolic process (GO:0009141) | 0.02200000 |
| regulation of postsynaptic membrane neurotransmitter receptor levels (GO:0099072) | 0.02230000 |
| positive regulation of cell junction assembly (GO:1901890) | 0.02240000 |
| negative regulation of cell-cell adhesion (GO:0022408) | 0.02380000 |
| cellular respiration (GO:0045333) | 0.02380000 |
| regulation of cell-matrix adhesion (GO:0001952) | 0.02410000 |
| cholesterol efflux (GO:0033344) | 0.02420000 |
| protein localization to extracellular region (GO:0071692) | 0.02630000 |
| regulation of plasminogen activation (GO:0010755) | 0.02710000 |
| regulation of endocytosis (GO:0030100) | 0.02820000 |
| positive regulation of cell-matrix adhesion (GO:0001954) | 0.02840000 |
| animal organ morphogenesis (GO:0009887) | 0.02950000 |
| negative regulation of cellular process (GO:0048523) | 0.03020000 |
| negative regulation of endothelial cell migration (GO:0010596) | 0.03190000 |
| maintenance of location (GO:0051235) | 0.03220000 |
| aerobic respiration (GO:0009060) | 0.03260000 |
| motor neuron axon guidance (GO:0008045) | 0.03380000 |
| chromatin organization (GO:0006325) | 0.03520000 |
| cellular response to amino acid stimulus (GO:0071230) | 0.03550000 |
| bundle of His cell to Purkinje myocyte communication (GO:0086069) | 0.03560000 |
| bone trabecula morphogenesis (GO:0061430) | 0.03560000 |
| regulation of extracellular matrix organization (GO:1903053) | 0.03560000 |
| positive regulation of nervous system development (GO:0051962) | 0.03560000 |
| homeostatic process (GO:0042592) | 0.03560000 |
| glucose catabolic process (GO:0006007) | 0.03570000 |
| neuroblast proliferation (GO:0007405) | 0.03580000 |
| negative regulation of cell population proliferation (GO:0008285) | 0.03590000 |
| macromolecule metabolic process (GO:0043170) | 0.03610000 |
| negative regulation of cell projection organization (GO:0031345) | 0.03650000 |
| regulation of cell cycle process (GO:0010564) | 0.03800000 |
| macromolecule localization (GO:0033036) | 0.04050000 |
| tube development (GO:0035295) | 0.04120000 |
| lipoprotein catabolic process (GO:0042159) | 0.04250000 |
| chaperone-mediated autophagy (GO:0061684) | 0.04270000 |
| protein targeting to lysosome involved in chaperone-mediated autophagy (GO:0061740) | 0.04280000 |
| positive regulation of keratinocyte apoptotic process (GO:1902174) | 0.04290000 |
| negative regulation of immune effector process (GO:0002698) | 0.04370000 |
| positive regulation of hormone secretion (GO:0046887) | 0.04380000 |
| cell cycle process (GO:0022402) | 0.04400000 |
| gluconeogenesis (GO:0006094) | 0.04430000 |
| collagen biosynthetic process (GO:0032964) | 0.04510000 |
| nucleoside phosphate catabolic process (GO:1901292) | 0.04510000 |
| purine ribonucleotide metabolic process (GO:0009150) | 0.04680000 |
| regulation of extent of cell growth (GO:0061387) | 0.04690000 |
| neural crest cell development (GO:0014032) | 0.04690000 |
| regulation of protein catabolic process (GO:0042176) | 0.04690000 |
| regulation of cellular response to growth factor stimulus (GO:0090287) | 0.04850000 |
| positive regulation of protein metabolic process (GO:0051247) | 0.04890000 |
| negative regulation of cytokine production (GO:0001818) | 0.04900000 |
